# Synthesis of 6″-Modified Kanamycin A Derivatives and Evaluation of Their Antibacterial Properties

**DOI:** 10.3390/pharmaceutics15041177

**Published:** 2023-04-07

**Authors:** Kseniya Shapovalova, Georgy Zatonsky, Natalia Grammatikova, Ilya Osterman, Elizaveta Razumova, Andrey Shchekotikhin, Anna Tevyashova

**Affiliations:** 1 Gause Institute of New Antibiotics, 11 B. Pirogovskaya, 119021 Moscow, Russia; schapowalowa.kseniya2014@yandex.ru (K.S.); gzatonsk@gmail.com (G.Z.); ngrammatikova@yandex.ru (N.G.); shchekotikhin@mail.ru (A.S.); 2 Center of Life Sciences, Skolkovo Institute of Science and Technology, Bolshoy Boulevard 30, bld. 1, 121205 Moscow, Russia; osterman@yandex.ru; 3 Center for Translational Medicine, Sirius University of Science and Technology, Olympic Avenue 1, 354340 Sochi, Russia; 4 Department of Chemistry, Lomonosov Moscow State University, Leninskie Gory 1, 119991 Moscow, Russia; elizaveta_razumova@list.ru

**Keywords:** antimicrobial resistance, aminoglycosides, kanamycin, chemical modification, mode of action, translation inhibition

## Abstract

Aminoglycosides are one of the first classes of antibiotics to have been used clinically, and they are still being used today. They have a broad spectrum of antimicrobial activity, making them effective against many different types of bacteria. Despite their long history of use, aminoglycosides are still considered promising scaffolds for the development of new antibacterial agents, particularly as bacteria continue to develop resistances to existing antibiotics. We have synthesized a series of 6″-deoxykanamycin A analogues with additional protonatable groups (amino-, guanidino or pyridinium) and tested their biological activities. For the first time we have demonstrated the ability of the tetra-*N*-protected-6″-*O*-(2,4,6-triisopropylbenzenesulfonyl)kanamycin A to interact with a weak nucleophile, pyridine, resulting in the formation of the corresponding pyridinium derivative. Introducing small diamino-substituents at the 6″-position of kanamycin A did not significantly alter the antibacterial activity of the parent antibiotic, but further modification by acylation resulted in a complete loss of the antibacterial activity. However, introducing a guanidine residue led to a compound with improved activity against *S. aureus*. Moreover, most of the obtained 6″-modified kanamycin A derivatives were less influenced by the resistant mechanism associated with mutations of the elongation factor G than the parent kanamycin A. This suggests that modifying the 6″-position of kanamycin A with protonatable groups is a promising direction for the further development of new antibacterial agents with reduced resistances.

## 1. Introduction

The overuse and misuse of antibiotics, both in human medicine and agriculture, has led to a concerning increase in antimicrobial resistance (AMR) [1]. AMR is a significant public health threat that limits our ability to treat infections effectively, leading to longer hospital stays, higher healthcare costs and increased mortality rates. The 2017 WHO report has identified a list of twelve antibiotic-resistant “priority pathogens”, nine of which are Gram-negative bacteria [2]. These bacteria are particularly challenging to treat due to their unique outer membrane structures, which limit the effectiveness of many commonly used antibiotics. According to expert estimates, the problem of AMR may worsen as a consequence of the COVID-19 coronavirus infection pandemic, as most of those who became ill were given antimicrobials to prevent or treat bacterial complications [3,4].

**Figure 1:**
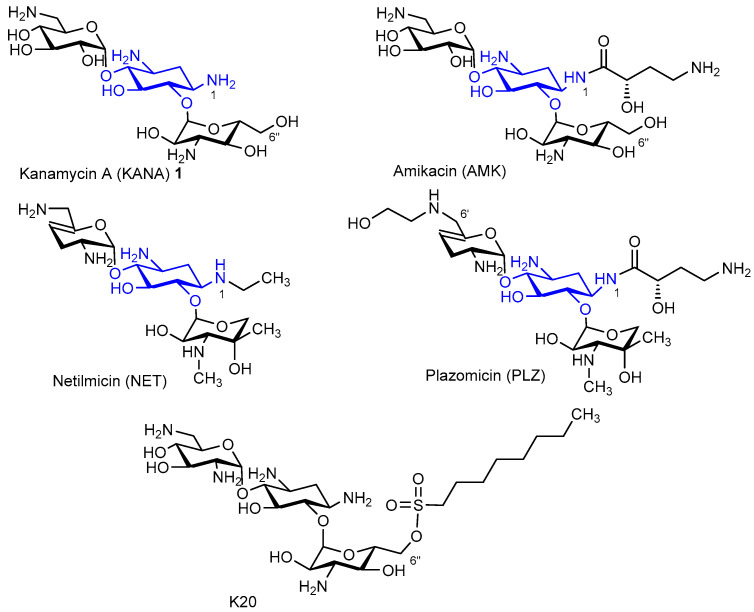
Structures of selected aminoglycoside antibiotics.

Aminoglycosides (AG) (Figure 1) are one of the first classes of antibiotics that were discovered, and they are still relevant today due to their broad-spectrum antimicrobial activity. They are active against gram-positive (G+) and gram-negative bacteria (G−), as well as mycobacteria [5]. AG are commonly used for the treatment of infections caused by G- bacteria, aerobic bacilli and staphylococci. They are often used in combination with other antibiotics such as β-lactams or vancomycin to improve their efficacy. Streptomycin, a member of the AG family, is still used in combination therapy to treat *Mycobacterium tuberculosis* [6]. Other AG such as kanamycin (KANA, **1**) and amikacin (Figure 1) are used as second-line drugs in the treatment of resistant *M. tuberculosis* infections [7].

The mechanism of action of aminoglycosides is based on inhibition of protein synthesis as a result of binding with the 16S rRNA of the bacterial ribosome 30S-subunit [8,9]. It was demonstrated that different classes of AG might bind to various sites of the 16S rRNA. Nevertheless, the common outcome is a change of a conformation of the A site to one that mimics the closed state induced by the interaction between cognate tRNA and mRNA. This leads to the inhibition of the proofreading capabilities of the ribosome and the promotion of mistranslation, ultimately inhibiting protein synthesis [10]. Additionally, aminoglycoside binding can also affect the translocation process catalyzed by the elongation factor G (EF-G) [11].

Aminoglycoside antibiotics have been widely used since the 1940s, but their effectiveness has been limited by the emergence of bacterial resistance. In addition to resistance, AG can also cause serious side effects, such as kidney damage, hearing loss and vestibular toxicity [12,13]. Since the 1970s, attempts have been made to obtain semi-synthetic aminoglycoside antibiotics; as a result, amikacin (AMK) and netilmicin (NET) were introduced into clinical practice in 1976 and 1981, respectively (Figure 1). More recently, plazomicin (PLZ, Figure 1) has been developed to overcome the resistance mechanisms that have evolved against older aminoglycosides. PLZ is a new generation aminoglycoside that has been designed to evade the most common aminoglycoside-modifying enzymes (AMEs) produced by bacteria. The FDA approved plazomicin in 2018 for the treatment of complicated urinary tract infections, including pyelonephritis, caused by certain gram-negative bacteria. Its approval marked an important step in the rejuvenation of the aminoglycoside class of antibiotics [14,15].

It was established that the major causes of antimicrobial resistance to AG are caused by a reduction in intracellular concentrations of antibiotics by bacterial efflux pumps or through reduced membrane permeability as well as the decreased affinity of the drug for its target bacterial ribosome, either by modifications of the drug or of the ribosome. The most clinically important mechanisms of resistance to AG are due to structural modifications of AGs by AMEs, which recatalyze regioselective modifications of the aminoglycoside molecule, such as *N*-acetylation, *O*-phosphorylation or an attachment of a nucleotide at one of the hydroxyl group of AG. Recently, it has also been demonstrated that some bacterial species, including *Pseudomonas aeruginosa*, *Escherichia coli* and *Acinetobacter baumannii*, quickly develop point mutations in the gene *fusA* (encoding the EF-G protein) when grown in sublethal concentrations of aminoglycosides such as kanamycin, tobramycin and gentamicin [16,17]. These mutations were reported as a novel mechanism of aminoglycoside resistance in the clinical strains of *P. aeruginosa* [18].

It is a valid approach to synthesize new derivatives of aminoglycosides to address the challenges posed by antimicrobial resistance and the limitations of existing antibiotics. Thus, we aimed to synthesize a series of new derivatives of AG in an attempt to find new compounds which possess activity against resistant strains of microorganisms (including strains with characterized mutations in the gene *fusA* (EF-G)) and/or with improved pharmacological properties.

**Figure 2:**
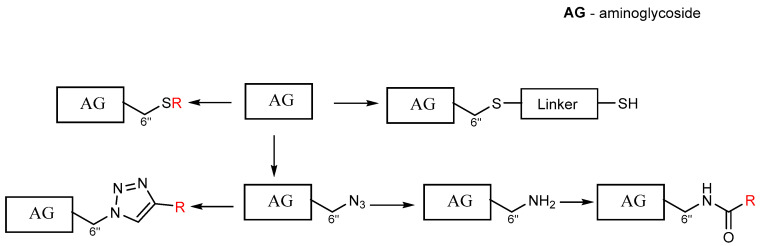
Types of semisynthetic AG derivatives obtained by the chemical transformation of the 6″-hydroxy group.

Kanamycin A, one of the first representatives of aminoglycosides and a starting compound for the semi-synthetic antibiotic amikacin [19], was chosen as a starting scaffold for chemical modification. The primary hydroxyl group in the 6″ position was selected for modification due to its availability and potential for selective transformation. Recently, comprehensive reviews have discussed various types of modifications of the aminoglycoside family of antibiotics [5,20]. Among those, different modifications of the primary hydroxyl group in the 6″ position of the antibiotic (AMK, KANA, kanamycin B and tobramycin) (Figure 2) were described, including the introduction of dithiol or azide groups, as well as an obtaining of the thioethers [20].

Furthermore, the replacement of hydroxyl moieties by amines on the cores of various AGs was performed to explore the importance of electrostatic interactions for the binding between RNA and AGs and to identify a common recognition pattern [21]. These results have demonstrated that the presence of several amino groups on an AG core, including the KANA series, correlates with an increase in inhibitory activity, as well as an increase in binding with the 16S rRNA of the bacterial ribosome 30S-subunit [20].

Interestingly, the modification of KANAs at the 6″-position also extended their potential application as antifungal drugs or fungicides for agriculture [22,23,24]. For example, the KANA derivative K20 (Figure 1) has been shown to exhibit antifungal activity against various fungi, including *Fusarium graminearum*, a pathogen that causes fusariosis in wheat [25,26].

Herein, we describe synthesis of a new series of 6″-modified KANA derivatives bearing additional groups that can be protonated at physiological pH. Varying the structure of the introduced fragment (alkyl amino-, guanidino- heterocyclic and hydroxyalkylamino) was carried out in order to established structure–antimicrobial-activity relationships, including an ability to overcome resistance of microorganisms caused by mutations in the gene *fusA* (EF-G).

## 2. Materials and Methods

### 2.1. Chemistry

Kanamycin A was a commercially available product of Abcr GmbH (Karlsruhe, Germany). All other solvents and reagents were commercially available products from Aldrich (Saint Louis, MO, USA) and Merck (Darmstadt, Germany). DMF (dried over CaCl_2_, then over P_2_O_5_) and pyridine (dried over KOH, then over CaCl_2_) were distilled before use.

The course of reactions, purification and extraction procedures, and the identities of the obtained compounds, were monitored by TLC and HPLC methods. TLC was performed on plates with 60F254 silica gel (Merck, Darmstadt, Germany). Kanamycin and its derivatives were visualized on chromatograms in iodine vapor or by a solution of ninhydrin in ethanol or 6N sulfuric acid solution followed by heating. UV-absorbing derivatives were also detected in UV light.

The preparative isolation and purification of the compounds were performed on silica gel columns (Kieselgel G60, 0.040–0.063 mm, Merck, Darmstadt, Germany). For neutralization, DOWEX 50 WX2 (100–200 mesh) ion-exchange resin (SERVA, Heidelberg, Germany) was used. All solutions were dried over sodium sulfate and evaporated in a vacuum at a temperature below 40 °C.

Analytical HPLC was performed on an LC-20AD chromatograph (Shimadzu, Kyoto, Japan) using a UV detector and a Kromasil-100-C18, 4.6250 mm column (Phenemenex, Torrance, CA, USA) at a flow rate of 1 mL/min in systems:

System (A1): A (0.01M H_3_PO_4_, pH 2.6) and B (MeCN), linear acetonitrile concentration gradient from 20→80% acetonitrile in 30 min, then from 80→20% in 3 min;

System (A2): A (HCOONH_4_ 0.2%, pH 4.5) and B (MeCN), linear acetonitrile concentration gradient from 40→90% for 30 min, then from 90→40% for 3 min; 

System (A3): A (0.01M H_3_PO_4_, pH 2.6) and B (MeCN), linear acetonitrile concentration gradient from 80→95% for 30 min, then from 95→80% for 3 min;

System (A4): A (HCOONH_4_ 0.2%, pH 4.5) and B (MeCN), linear acetonitrile concentration gradient from 20→90% for 30 min, then from 90→95% for 5 min, then from 95→95% for 5 min, then from 95→20% for 5 min;

System (A5): A (HCOONH_4_ 0.2%, pH 8.4) and B (MeCN), linear acetonitrile concentration gradient from 40→90% for 30 min, then from 90→40% for 3 min;

System (A6): A (HCOONH_4_ 0.2%, pH 8.4) and B (MeCN), linear acetonitrile concentration gradient from 10→60% for 30 min, then from 60→10% for 3 min.

The purities of the intermediates and final compounds were at least 90% by HPLC (for UV-absorbing compounds).

Elemental analysis was performed on the automated PerkinElmer 2400 CHN microanalyzer. High-resolution mass spectra (HR MS) were obtained by electrospray ionization (ESI) on a micrOTOF-Q II instrument (Bruker Daltonics GmbH, Bremen, Germany). The ^1^H, ^13^C and ^15^N NMR spectra were recorded in DMSO-d6 at 25 °C on a Bruker AV III 500 spectrometer (Bruker Biospin AG, Fällanden, Switzerland) at 500.2 MHz, 125.8 MHz and 50.7 MHz for ^1^H, ^13^C and ^15^N, respectively. Signal assignment in NMR spectra was performed using two-dimensional COSY, TOCSY, NOESY, HSQC, HMBC and ^1^H-^15^N edHSQC experiments. Standard pulse sequences were used. The ^1^H and ^13^C NMR spectra were referenced using either residual DMSO solvent signals (2.50 ppm for DMSO-d6 for ^1^H spectra and 49.5 ppm for DMSO-d6 for ^13^C spectra) or signals of internal DSS reference for D_2_O solutions. The ^1^H-^15^N edHSQC spectrum was externally referenced against CH_3_NO_2_ for ^15^N.

1,3,6′,3″-Tetra-*N*-Cbz-6″-*O*-(2,4,6-triisopropylbenzenesulfonyl)kanamycin A (**2**)

1st step

The solution of benzylchloroformate (2.40 mL, 16.7 mmol) in acetone (10 mL) was added dropwise at 0 °C to the mixture kanamycin A monosulfate (2.0 g, 3.43 mmol) and saturated water solution of Na_2_CO_3_ (26.7 mL). The reaction mixture was stirred at 0 °C for 2 h, then at rt for 8 h. The resulting precipitate was filtered off and then suspended in 1M HCl (70 mL) and stirred for 30 min. The precipitate was filtered off, washed with H_2_O and dried in a vacuum over P_2_O_5_. The target 1,3,6′,3″-tetra-*N*-Cbz-kanamycin A was obtained as a white solid (3.44 g, 98% yield) and used without the additional purification at the next stage.

HRMS (ESI): calculated for [C_50_H_61_N_4_O_19_]^+^ = 1021.3930 [C_50_H_64_N_5_O_19_]^+^ = 1038.4196 [C_50_H_60_N_4_O_19_Na]^+^ = 1043.3749, found [M+H]^+^ = 1021.4185 [M+NH_4_]^+^ = 1038.4467 [M+Na]^+^ = 1043.4044, R_f_ (CHCl_3_:MeOH 10:1) = 0.5, R_t_ (system A2) = 13.41 min.

^1^H NMR (δ, ppm, J/Hz): 1.46–3.56 (m, 7H, cHex), 3.07–4.93 (m, 7H, CH_1′-6′_), 3.31–4.99 (m, 7H, CH_1″-6″_), 3.67 (d, 2H, ^3^J = 6.1 Hz, -OH-CH_CH2″,CH4″-_), 4.23 (t, 1H, ^3^J = 5.6 Hz, -OH-CH_2 CH6″-_), 4.85–5.18 (m, 8H, -OCH_2_), 5.24 (bs, 1H, -OH-CH _cHex5_), 5.34 (d, 1H, ^3^J = 5.6 Hz, -OH-CH_CH2′-CH4′_-), 6.84 (s, 1H, -NH-CH_2 CH6′_-), 7.01 (s, 1H, -NH-CH_2 CH3″_-), 7.17 (s, 1H, -NH-CH_cHex3_-), 7.21–7.42 (m, 20H, 4 × Ph), 7.43 (s, 1H, -NH-CH_cHex1_-).

^13^C NMR (δ, ppm, J/Hz): 34.4, 41.7, 49.7, 50.1, 56.5, 60.2, 65.0, 65.3, 67.2, 70.1, 70.6, 70.7, 72.3, 72.7, 72.9, 74.1, 80.4, 84.3, 97.3, 101.1, 127.7, 128.3, 136.8–137.4, 155.6, 155.9, 156.5, 156.6.

2nd step

The 2,4,6-Triisopropylbenzenesulfonylchloride (1.28 g, 4.23 mmol) was added to the solution of 1,3,6′,3″-tetra-*N*-Cbz-kanamycin A (1.0 g, 0.98 mmol) and 4-(*N*,*N*-dimethylamino)pyridine (517 mg, 4.23 mmol) in dry pyridine (15 mL). The reaction mixture was stirred 6 h at room temperature (rt), then an additional amount of 2,4,6-triisopropylbenzenesulfonylchloride (1.28 g, 4.23 mmol) was added and the reaction mixture was stirred at rt for 20 h. An aqueous solution of HCl (1N, 70 mL) and water (100 mL) was added to the reaction mixture. The product was extracted with ethyl acetate (3 × 60 mL) and organic fractions were combined, dried over Na_2_SO_4_, filtered off and evaporated to dryness. The target compound was purified by the column chromatography on silica gel. The elution was carried out by CHCl_3_ (200 mL), followed by the mixture CHCl_3_-CH_3_OH (100:3). Fractions which contained the target compound were combined and evaporated to dryness, resulting in **2** (1.12 g, 89% yield) as a light-yellow solid.

HRMS (ESI): calculated for [C_65_H_86_N_5_O_21_S]^+^ = 1304.5536, found [M+NH_4_]^+^ = 1304.5655, R_f_ (CHCl_3_:MeOH 100:15) = 0.57, R_t_ (system A3) = 8.31 min.

^1^H NMR (δ, ppm, J/Hz): 1.19–1.21 (m, 18H, 6×-CH_3_), 1.41–3.55 (m, 7H, cHex), 3.06–4.89 (m, 7H, CH_1′-6′_), 3.29–4.97 (m, 7H, CH_1″-6″_), 3.67 (d, 2H, ^3^J = 6.1 Hz, -OH-CH_CH2″,CH4″_-), 3.99–4.06 (m, 3H, -CH(CH_3_)_3_), 4.86–5.11 (m, 8H, -OCH_2_), 5.24 (bs, 1H, -OH-CH_cHex5_), 5.34 (d, 1H, ^3^J = 5.6 Hz, -OH-CH_CH2′-CH4′-_), 6.83 (s, 1H, -NH-CH_2 CH6′_-), 7.06 (s, 1H, -NH-CH_2 CH3″_-), 7.18 (s, 1H, -NH-CH_cHex3_-), 7.25–7.39 (m, 20H, 4 × Ph), 7.34 (s, 1H, -NH-CH_cHex1_-).

^13^C NMR (δ, ppm, J/Hz): 23.2, 24.37, 24.45, 29.0, 34.5, 41.6, 49.6, 50.0, 56.6, 65.0, 65.3, 66.4, 67.5, 69.6, 69.7, 70.4, 70.6, 72.2, 72.6, 74.2, 80.0, 84.6, 97.4, 101.2, 123.7, 127.6, 128.2, 137.0, 137.1, 137.2, 150.2, 153.6, 155.5, 155.8, 156.5, 156.6.

1,3, 6′,3″-Tetra-*N*-Boc-6″-*O*-(2,4,6-triisopropylbenzeneosulfonyl)kanamycin A (**3**)

1st step

The solution of di-*tert*-butyldicarbonate (1.5 g, 6.86 mmol) and Et_3_N (0.5 mL, 3.43 mmol) in DMSO (12 mL) was added to a suspension of kanamycin A monosulfate (500 mg, 0.86 mmol) and NaOH (34 mg, 0.858 mmol) in H_2_O (2 mL). The reaction mixture was stirred at rt for 12 h, then NH_4_OH (conc, 5 mL) was added. The obtained solid was filtered over celite, and a cake was washed with H_2_O (2 × 20 mL) and ethyl acetate (20 mL). The target compound was eluted from celite with methanol (50 mL) and the obtained solution was evaporated to dryness, resulting in 1,3, 6′,3″-tetra-*N*-Boc-kanamycin A (0.47 g, 62% yield) as white solid which was used without the additional purification at the next stage.

HRMS (ESI): calculated for [C_38_H_68_N_4_O_19_Na]^+^ = 907.4376, found [M+Na]^+^ = 907.4533, R_f_ (MeOH:NH_3_ 7:10) = 0.36.

^1^H NMR (δ, ppm, J/Hz): 1.36 (s, 9H, 3×-CH_3_), 1.37 (s, 9H, 3×-CH_3_), 1.38 (s, 18H, 6×-CH_3_) 1.38–3.42 (m, 7H, cHex), 3.04–4.88 (m, 7H, CH_1′-6′_), 2.9–4.89 (m, 7H, CH_1″-6″_), 3.69 (d, 2H, ^3^J = 6.1 Hz, -OH-CH_CH2″,CH4″_-), 4.21 (t, 1H, ^3^J = 5.6 Hz, -OH-CH_2 CH6‴_-), 5.25 (bs, 1H, -OH-CH _cHex5_), 5.35 (d, 1H, ^3^J = 5.6 Hz, -OH-CH_CH2′-CH4′_-), 6.35 (s, 1H, -NH-CH_2CH6′_-), 6.5 (s, 1H, -NH-CH_2 CH3″_-), 6.58 (s, 1H, -NH-CH_cHex3_-), 6.96 (s, 1H, -NH-CH_cHex1_-).

^13^C NMR (δ, ppm, J/Hz): 28.1, 28.2, 28.3, 34.7, 41.4, 49.1, 50.0, 55.9, 60.3, 67.5, 70.1, 70.3, 70.5, 72.1, 72.7, 72.9, 75.0, 77.8, 80.4, 83.9, 97.8, 101.1, 154.9, 155.3, 156.1, 156.3.

2nd step

The 2,4,6-Triisopropylbenzenesulfonylchloride (1.74 g, 5.74 mmol) was added to the mixture of 1,3,6′,3″-tetra-*N*-Boc-kanamycin A (1.18 g, 1.33 mmol) and 4-(*N*,*N*-dimethylanimo)pyridine (702 mg, 5.74 mmol) in dry pyridine (30 mL). The reaction mixture was stirred at rt for 6 h, then an additional portion of 2,4,6-triisopropylbenzenesulfonylchloride (1.74 g, 5.74 mmol) was added and the reaction mixture was stirred at rt for 20 h. An aqueous solution of HCl (1N, 70 mL) and H_2_O (100 mL) was added to the reaction mixture. The target compound was extracted with ethyl acetate (3 × 60 mL) and the organic fraction was dried over Na_2_SO_4_, filtered off and concentrated in a vacuum. The product was purified by column chromatography in silica gel and the column was eluted by CHCl_3_ (150 mL), followed by the mixture of CHCl_3_-CH_3_OH (100:2). Fractions which contained the target compound were combined and evaporated to dryness, resulting in **3** (0.59 g, 39% yield) as a light-yellow solid.

HRMS (ESI): calculated for [C_53_H_91_N_4_O_21_S]^+^ = 1151.5896, found [M+H]^+^ = 1151.5847, R_f_ (CHCl_3_:MeOH 10:1) = 0.31, R_t_ (system A1) = 9.44 min.

6″-(Piperidin-1-yl)-6″-deoxykanamycin A acetate (**4**)

1st step

Compound **2** (200 mg, 0.156 mmol) was dissolved in dry pyridine (5 mL) and NaI (2 mg, 0.016 mmol) and iPr_2_EtN (0.1 mL, 0.77 mmol) were added to the reaction mixture. The reaction mixture was refluxed for 1 h, then cooled to rt and diluted with H_2_O (50 mL). The product was extracted with ethyl acetate (3 × 30 mL) and the organic fractions were combined, dried over Na_2_SO_4_ and evaporated to dryness. The residue was purified by flash-chromatography on silica gel. The elution was carried out by CHCl_3_ (50 mL), followed by the mixture CHCl_3_-CH_3_OH-NH_4_OH (10:3:1). Fractions which contained the target compound were combined and evaporated to dryness, resulting in 1,3,6′,3″-tetra-*N*-Cbz-6″-(pyridin-1-ium)-6″-deoxykanamycin A (167 mg, 98% yield) as a white solid.

HRMS (ESI): calculated for [C_55_H_64_N_5_O_18_]^+^ = 1082.4246, found [M]^+^ = 1082.4249, R_f_ (CHCl_3_:MeOH 100:15) = 0.17, R_t_ (system A2) = 11.09 min.

^1^H NMR (δ, ppm, J/Hz): 1.43–3.46 (m, 7H, cHex), 3.1–4.56 (m, 7H, CH_1′-6′_), 3.15–4.95 (m, 7H, CH_1″-6″_), 4.86–5.11 (m, 8H, -OCH_2_), 6.78 (t, 1H, ^3^J = 4.9 Hz, -NH-CH_2 CH6′_-), 7.1 (s, 1H, -NH-CH_2 CH3″_-), 7.14 (s, 1H, -NH-CH_cHex3_-), 7.25–7.39 (m, 20H, 4×Ph), 7.39 (s, 1H, -NH-CH_cHex1_-) 8.12 (s, 2H, Py_3′_), 8.58 (s, 1H, Py_4′_), 8.81 (s, 2H, Py_2′_).

^13^C NMR (δ, ppm, J/Hz): 33.7, 41.2, 49.1, 50.3, 55.9, 61.9, 65.0, 65.3, 68.9, 69.1, 70.2, 70.3, 71.5, 72.2, 73.9, 79.1, 84.7, 98.1, 101.0, 127.1, 127.6, 128.2, 145.0, 145.4, 155.5, 155.8, 156.5, 156.6.

2nd step

1,3,6′,3″-Tetra-*N*-Cbz-6″-( pyridin-1-ium)-6″-deoxykanamycin A (54 mg, 0,05 mmol) was dissolved in CH_3_OH (2 mL), then Pd/C (5%, 85 mg) was added and the reaction mixture was acidified to pH 3 by the addition of CH_3_COOH. The reaction mixture was vigorously stirred at rt at H_2_ flow (1 atm) for 3 h. The catalyst was filtered off via celite; the cake was washed with CH_3_OH (5 mL) and H_2_O (5 mL), and the combined filtrate was concentrated in a vacuum. The target compound was precipitated by the addition of acetone (10 mL), filtered off and dried in a vacuum over P_2_O_5,_ resulting in compound **4** (25 mg, 58% yield) as a white solid.

Anal. calculated for [C_23_H_44_N_5_O_10_×5AcOH×5H_2_O]: C, 42.12; H, 7.93; N, 7.44, O 42.51. Found: C, 42.10; H, 7.90; N, 7.43.

HRMS (ESI): calculated for [C_23_H_45_N_5_O_10_]^+^ = 552.3245, found [M+H]^+^ = 552.3216, R_f_ (NH_4_OH:iPrOH 10:7) = 0.31, T_mp_ = 201 °C (decomp.). The assignment of the signals in the ^1^H and ^13^C NMR spectra is presented in the Table S1.

6″-(Pyridin-1-ium)-6″-deoxykanamycin A trifluoroacetate (**5**)

1st step

Compound **3** (60 mg, 0.052 mmol) was dissolved in dry pyridine (2 mL), then NaI (1 mg, 0.005 mmol) and iPr_2_EtN (34 mg, 0.26 mmol) were added to the reaction mixture. The obtained solution was refluxed for 1 h, then cooled to room temperature and diluted with H_2_O (40 mL). The product was extracted with ethyl acetate (3 × 15 mL) and the organic fractions were combined, dried over Na_2_SO_4_, filtered off and evaporated to dryness in a vacuum. The product was purified by flash chromatography on silica gel and the elution was carried by the mixture CHCl_3_-CH_3_OH (10:1, 25 mL) followed by the mixture CHCl_3_-CH_3_OH-NH_4_OH (10:5:3). Fractions which contained the target compound were combined and evaporated to dryness in a vacuum, resulting in 1,3,6′,3″-tetra-*N*-Boc-6″-(pyridin-1-ium)-6″-deoxykanamycin A (35 mg, 71% yield) as a white solid.

HRMS (ESI): calculated for [C_43_H_72_N_5_O_18_]^+^ = 946.4872, found [M]^+^ = 946.4979, R_f_ (CHCl_3_:MeOH 100:15) = 0.15.

2nd step

Trifluoroacetic acid (0.2 mL, 2.752 mmol) was added dropwise to the solution of 1,3,6′,3″-tetra-*N*-Boc-6″-(pyridin-1-ium)-6″-deoxykanamycin A (30 mg, 0.03 mmol) in CH_2_Cl_2_ (1 mL). The reaction mixture was stirred at rt for 4 h and then evaporated to dryness in a vacuum. The addition of Et_2_O (10 mL) to the residue resulted in the formation of the precipitate, which was filtered off, washed with Et_2_O (50 mL) and dried in a vacuum over P_2_O_5_, resulting in the target compound **5** (23 mg, 63% yield) obtained as a white solid.

HRMS (ESI): calculated for [C_23_H_40_N_5_O_10_]^+^ = 546.2775, found [M]^+^ = 546.2752, R_f_ (NH_4_OH:iPrOH 10:7) = 0.3, T_mp_ = 205 °C (decomp.).

The assignment of the signals in the ^1^H and ^13^C spectra is presented in Table S1.

6″-(2-Aminoethylamino)-6″-deoxykanamycin A acetate (**6**)

1st step

6″-(2-Aminoethylamino)-1,3,6′,3″-tetra-*N*-Cbz-6″-deoxykanamycin A (**6a**)

A mixture of compound **2** (805 mg, 0.626 mmol) and ethylenediamine (3.5 mL) was stirred at rt for 24 h, then the reaction mixture was diluted with H_2_O (150 mL). The forming precipitate was filtered off, washed with H_2_O and dried in a vacuum at 45 °C and then in a vacuum over P_2_O_5_. The target 6″-(2-aminoethylamino)-1,3,6′,3″-tetra-*N*-Cbz-6″-deoxykanamycin A (**6a**) (612 mg, 92%) was obtained as a light-yellow solid and used without additional purification on the next stage.

HRMS (ESI): calculated for [C_52_H_67_N_6_O_18_]^+^ = 1063.4511 [C_52_H_66_N_6_O_18_Na]^+^ = 1085.1150 [C_52_H_66_N_6_O_18_K]^+^ = 1101.4071, found [M+H]^+^ = 1063.4506 [M+Na]^+^ = 1085.1326 [M+K]^+^ = 1101.4065, R_f_ (CHCl_3_:MeOH:HCOOH 13:5:0.1) = 0.45, R_t_ (System A4) = 17.5 min.

2nd step

6″-(2-Aminoethylamino)-1,3,6′,3″-tetra-*N*-Cbz-6″-deoxykanamycin A (**6a**) (61 mg, 0.057 mmol) was dissolved in CH_3_OH (2 mL) and 5% Pd/C (95 mg) was added to the reaction mixture. The CH_3_COOH was added to the reaction mixture until pH 3 was reached, and the mixture was vigorously stirred at H_2_ flow (1 atm.) for 4 h. The catalyst was filtered off via a celite layer; the cake was washed with CH_3_OH (5 mL) and H_2_O (5 mL), and the combined filtrate was concentrated in a vacuum. The target compound was precipitated by the addition of acetone (10 mL), filtered off and dried in a vacuum over P_2_O_5_, resulting in the target compound **6** (32.8 mg, 69%) as a white solid.

Anal. calculated for [C_20_H_42_N_6_O_10_×5AcOH×3H_2_O]: C, 40.91; H, 7.78; N, 9.54, O 41.77. Found: C, 40.91; H, 7.63; N, 9.50.

HRMS (ESI): calculated for [C_20_H_43_N_6_O_10_]^+^ = 527.3040, found [M+H]^+^ = 527.3032, R_f_ (NH_4_OH:iPrOH 10:7) = 0.32, T_mp_ = 181 °C (decomp.).

The assignment of the signals in the ^1^H and ^13^C spectra is presented in Table S1.

6″-(3-Aminopropyl-1-amino)-6″-deoxykanamycin A acetate (**7**)

1st step

6″-(3-Aminopropyl-1-amino)-1,3,6′,3″-tetra-*N*-Cbz-6″-deoxykanamycin A (**7a**)

A mixture of compound **2** (200 mg, 0.156 mmol) and propan-1,3-diamine (3.5 mL) was stirred at rt for 24 h, then the reaction mixture was diluted with H_2_O (100 mL). The forming precipitate was filtered off, washed with H_2_O and dried in a vacuum at 45 °C and then in a vacuum over P_2_O_5_ at rt. The target 6″-(3-aminopropyl-1-amino)-1,3,6′,3″-tetra-*N*-Cbz-6″-deoxykanamycin A (**7a**) (136 mg, 81%) was obtained as a white solid and used without additional purification on the next stage.

HRMS (ESI): calculated for [C_53_H_69_N_6_O_18_]^+^ = 1077.4668, found [M+H]^+^ = 1077.4667, Rf (CHCl_3_:MeOH:HCOOH 13:5:0.1) = 0.42, R_t_ (System A4) = 16.5 min.

2nd step

6″-(3-Aminopropyl-1-amino)-1,3,6′,3″-tetra-*N*-Cbz-6″-deoxykanamycin A (**7a**) (138 mg, 0.129 mmol) was dissolved in CH_3_OH (2.5 mL) and 5% Pd/C (180 mg) was added to the reaction mixture. The CH_3_COOH was added to the reaction mixture until pH 3 was reached, and the mixture was vigorously stirred at H_2_ flow (1 atm.) for 4 h. The catalyst was filtered off via a celite layer; the cake was washed with CH_3_OH (5 mL) and H_2_O (5 mL), and the combined filtrate was concentrated in a vacuum. The target compound was precipitated by the addition of acetone (10 mL), filtered off and dried in a vacuum over P_2_O_5_, resulting in the target compound **7** (103 mg, 95%) as a white solid.

Anal. calculated for [C_21_H_44_N_6_O_10_×5AcOH×1H_2_O]: C, 43.35; H, 7.75; N, 9.78, O 39.12. Found: C, 43.35; H, 7.75; N, 9.78.

HRMS (ESI): calculated for [C_21_H_45_N_6_O_10_]^+^ = 541.3197, found [M+H]^+^ = 541.3122, R_f_ (NH_4_OH:iPrOH 1:1) = 0.15, T_mp_ = 176 °C (decomp).

The assignment of the signals in the ^1^H and ^13^C spectra is presented in Table S1.

6″-(2-Hydroxyethyl-1-amino)-6″-deoxykanamycin A trifluoroacetate (**8**)

1st step

1,3,6′,3″-Tetra-*N*-Boc-6″-(2-hydroxyethyl-1-amino)-6″-deoxykanamycin A (**8a**)

A mixture of compound 3 (100 mg, 0.087 mmol) and 2-aminoethanol (1.5 mL) was stirred at rt for 24 h, then the reaction mixture was diluted with H_2_O (100 mL). The forming precipitate was filtered off, washed with H_2_O and dried in a vacuum at 45 °C and then in a vacuum over P_2_O_5_ at rt. The target 1,3,6′,3″-tetra-*N*-Boc-6″-(2-hydroxyethyl-1-amino)-6″-deoxykanamycin A (**8a**) (35 mg, 45%) was obtained as a white solid and used without additional purification on the next stage.

HRMS (ESI): calculated for [C_40_H_74_N_5_O_19_]^+^ = 928.4978, found [M+H]^+^ = 928.4988, R_f_ (CHCl_3_:MeOH:HCOOH 7:1:0.3) = 0.28.

^1^H NMR (δ, ppm, J/Hz): 1.36 (s, 9H, 3×-CH_3_), 1.37 (s, 9H, 3×-CH_3_), 1.38 (s, 18H, 6×-CH_3_), 1.47–3.47 (m, 7H, cHex), 2.90–4.91 (m, 7H, -CH_1″-6″_), 3.0 (s, 2H, -NH-CH_2_-), 3.1–4.92 (m, 7H, -CH_1′-6′_), 3.63 (s, 2H, -CH_2_-OH), 6.35 (bt, 1H, ^3^J = 4.5 Hz, -NH-CH_2 CH6′_-), 6.57 (d, 1H, ^3^J = 9.3 Hz, -NH-CH_2 CH3″_-), 6.64 (bd, 1H, ^3^J = 7.0 Hz, -NH-CH_cHex3_-), 6.97 (bs, 1H, -NH-CH_cHex1_-).

^13^C NMR (δ, ppm, J/Hz): 28.1–28.3, 34.5, 41.3, 48.5, 48.9, 49.8, 50.4, 55.6, 56.8, 68.4, 69.5, 69.9, 70.0, 70.5, 72.6, 75.4, 77.4–77.9, 78.0, 80.2, 84.3, 98.6, 101.1, 154.9–156.2.

2nd step

Trifluoroacetic acid (0.2 mL, 2.752 mmol) was added dropwise to a solution of 1,3,6′,3″-tetra-*N*-Boc-6″-(2-hydroxyethyl-1-amino)-6″-deoxykanamycin A (**8a**) (30 mg, 0.032 mmol) in CH_2_Cl_2_ (2 mL). The reaction mixture was stirred at rt for 24 h and then evaporated to dryness in a vacuum. The addition of acetone (10 mL) to the residue resulted in the formation of a white solid which was filtered off, washed with acetone (3 × 10 mL) and dried in a vacuum over P_2_O_5_. Finally, 6″-(2-Hydroxyethylamino)-6″-deoxykanamycin A (**8**) (10 mg, 27%) was obtained as a white solid in the form of trifluoroacetate.

Anal. calculated for [C_20_H_41_N_6_O_10_×5TFA×1H_2_O]: C, 32.35; H, 4.34; N, 7.55, F 25.59, O 30.17. Found: C, 32.35; H, 4.60; N, 7.50.

HRMS (ESI): calculated for [C_20_H_42_N_5_O_11_]^+^ = 528.2881, found [M+H]^+^ = 528.2870, R_f_ (NH_4_OH:iPrOH 1:1) = 0.32, T_mp_ = 186 °C (decomp.).

The assignment of the signals in the ^1^H and ^13^C spectra is presented in Table S1.

6″-((*S*)-7-((2-(4-Amino-2-hydroxybutanamido)ethyl)amino))-6″-deoxykanamycin A acetate (**9**)

1st step

Benzotriazol-1-yloxytripirrolidonophosphonium hexafluorophosphate (PyBoP) (196 mg, 0.39 mmol) was added portionwise to the solution of 6″-(2-aminoethylamino)-1,3,6′,3″-tetra-*N*-Cbz-6″-deoxykanamycin A (**6a**) (200 mg, 0.188 mmol) and (S)-4-(((benzyloxy)carbonyl)amino)-2-hydroxybutanoic acid (143 mg, 0.57 mmol) in DMSO (2 mL). The pH of the reaction mixture was kept ~8 by the addition of Et_3_N (~200 µL). The reaction mixture was stirred at rt for 48 h, then H_2_O (100 mL) was added. The forming precipitate was filtered off, washed with Et_2_O (50 mL), dried and purified by the column chromatography on silica gel. The elution was carried out by the mixture CHCl_3_:CH_3_OH:HCOOH (5:1:0.3). Fractions which contained the target compound were combined and evaporated to dryness, resulting in the target intermediate 6″-((*S*)-7-((2-(4-(benzyloxycarbonyl)amino-2-hydroxybutanamido)ethyl)amino))-1,3,6′,3″-tetra-*N*-Cbz-6″-deoxykanamycin A (164 mg, 67%) as a white solid.

HRMS (ESI): calculated for [C_64_H_80_N_7_O_22_]^+^ = 1298.53, found [M+H]^+^ = 1298.5398, R_f_ (CHCl_3_:MeOH:HCOOH 5:1:0.3) = 0.41, R_t_ (System A4) = 20.8 min.

^1^H NMR (δ, ppm, J/Hz): 1.51–3.56 (m, 7H, cHex), 1.57 (s, 1H, -CH_1′_- CH_2′_-), 1.83 (s, 1H, -CH_1′_- CH_2′_-), 2.65 (s, 2H, -CH_2_-NHCO), 2.66–5.00 (m, 7H, CH_1″-6″_), 3.08–4.98 (m, 7H, CH_1′-6′_), 3.1 (s, 2H, -CH_2′_- CH_3′_-), 3.18 (s, 2H, -NH-CH_2_-), 3.88 (s, 1H, -NHCO-CH_1′_-), 4.87–5.09 (m, 10H, -OCH_2_), 6.83 (t, 1H, ^3^J = 4.7 Hz, -NH-CH_2 CH6′_-), 7.02 (d, 1H, ^3^J = 9.0 Hz, -NH-CH_2 CH3″_-), 7.18 (d, 1H, ^3^J = 8.6 Hz, -NH-CH_cHex3_-), 7.22 (t, 1H, ^3^J = 5.5 Hz, -CH_3′_-NH-), 7.26–7.37 (m, 20H, 4×Ph), 7.41 (s, 1H, -NH-CH_cHex1_-), 7.81 (t, 1H, ^3^J = 5.3 Hz, -CH_2_-NH-CO).

^13^C NMR (δ, ppm, J/Hz): 34.3, 34.5, 37.1, 37.6, 41.6, 48.5, 49.6, 49.6, 50.2, 56.4, 64.9, 65.0, 65.1, 69.1, 69.4, 69.9, 70.5, 70.5, 70.7, 72.3, 72.7, 74.3, 80.4, 84.5, 97.4, 101.2, 127.5, 127.6, 128.2, 155.5, 155.9, 156.0, 156.4, 156.5, 164.5.

2nd step

The 6″-((*S*)-7-((2-(4-(Benzyloxycarbonyl)amino-2-hydroxybutanamido)ethyl)amino))-1,3,6′,3″-tetra-*N*-Cbz-6″-deoxykanamycin (150 mg, 0.116 mmol) was dissolved in CH_3_OH (3 mL), then 5% Pd/C (200 mg) was added and the reaction mixture was acidified to pH 3 by the addition of CH_3_COOH. The reaction mixture was vigorously stirred at rt at H_2_ flow (1 atm) for 2 h. The catalyst was filtered off via a celite layer; the cake was washed with CH_3_OH (5 mL) and H_2_O (5 mL), and the combined filtrate was concentrated in a vacuum. The target compound was precipitated by the addition of acetone (10 mL), filtered off and dried in a vacuum over P_2_O_5_, resulting in compound **9** in the form of acetate (97 mg, 91% yield) as a white solid.

Anal. calculated for [C_24_H_49_N_7_O_12_×5AcOH×1H_2_O]: C, 43.17; H, 7.57; N, 10.36, O 38.9. Found: C, 43.17; H, 7.57; N, 10.36.

HRMS (ESI): calculated for [C_24_H_50_N_7_O_12_]^+^ = 628.3517, found [M+H]^+^ = 628.3479, R_f_ (NH_4_OH:iPrOH 1:1) = 0.27, T_mp_ = 179 °C (decomp.).

The assignment of the signals in the ^1^H and ^13^C spectra is presented in Table S1.

6″-(2-(3-(1-Hydroxy-1,3-dihydrobenzo[c][1,2]oxaborol-7-yl)-*N*-(ethyl-1-amino)propanamide)-6″-deoxykanamycin acetate (**10**)

1st step

1,3,6′,3″-Tetra-*N*-Cbz-6″-(2-(3-(1-hydroxy-1,3-dihydrobenzo[c][1,2]oxaborol-7-yl)-*N*-(ethyl-1-amino)propanamide)-6″-deoxykanamycin A

PyBoP (98 mg, 0.188 mmol) was added portionwise to the solution of 6″-(2-aminoethylamino)-1,3,6′,3″-tetra-N-Cbz-6″-deoxykanamycin A (**6a**) (100 mg, 0.094 mmol) and 3-(1-hydroxy-1,3-dihydrobenzo[c][1,2]oxaborol-7-yl)propanoic acid (58 mg, 0.282 mmol) in DMSO (2 mL). The pH of the reaction mixture was kept ~8 by the addition of Et_3_N (~50 µL). The reaction mixture was stirred at rt for 24 h, then Et_2_O (50 mL) was added. The forming precipitate was filtered off, washed with Et_2_O (5 × 10 mL) and dried over P_2_O_5_, and the obtained product was used without additional purification on the next stage (70 mg, 60%).

HRMS (ESI): calculated for [C_62_H_76_BN_6_O_21_]^+^ = 1251.5156, found [M+H]^+^ = 1251.5124, R_f_ (CHCl_3_:MeOH:HCOOH 11.5:3:1.5) = 0.37, R_t_ (System A5) = 16.0 min

2nd step

The 1,3,6′,3″-Tetra-*N*-Cbz-6″-(2-(3-(1-hydroxy-1,3-dihydrobenzo[c][1,2]oxaborol-7-yl)-*N*-(ethyl-1-amino)propanamide)-6″-deoxykanamycin A (60 mg, 0.048 mmol) was dissolved in CH_3_OH (3.5 mL), then 5% Pd/C (85 mg) was added and the reaction mixture was acidified to pH 3 by the addition of CH_3_COOH. The reaction mixture was vigorously stirred at rt at H_2_ flow (1 atm) for 2 h. The catalyst was filtered off via celite layer; the cake was washed with CH_3_OH (5 mL) and H_2_O (5 mL), and the combined filtrate was concentrated in a vacuum. The target compound was precipitated by the addition of acetone (10 mL) and was filtered off and dried in a vacuum over P_2_O_5_, resulting in compound **10** (22 mg, 45% yield) as a white solid.

HRMS (ESI): calculated for [C_30_H_51_BN_6_O_13_] = 714.3607, found [M+H]^2+^ = 357.1795, R_f_ (NH_4_OH:iPrOH 10:7) = 0.3, R_t_ (System A6) = 20.2 min, T_mp_ = 245 °C (decomp.).

The assignment of the signals in the ^1^H and ^13^C spectra is presented in Table S1.

6″-(2-Guanidinoethylamino)-6″-deoxykanamycin A acetate (**11**)

1st step

1,3,6′,3″-Tetra-*N*-Cbz-6″-(2-guanidinoethylamino)-6″-deoxykanamycin A

Ethyldiisopropylamine (EDIA, 80 µL, 0.452 mmol) was added to mixture of 6″-(2-aminoethylamino)-1,3,6′,3″-tetra-*N*-Cbz-6″-deoxykanamycin A (**6a**) (160 mg, 0.151 mmol) and 1*H*-pyrazole-1-carboximidamide hydrochloride (33 mg, 0.25 mmol) in DMF (2 mL). The reaction mixture was stirred at rt for 24 h, then Et_2_O (50 mL) was added. The forming precipitate was filtered off, washed with Et_2_O (5 × 10 mL) and dried over P_2_O_5_, and the obtained product was used without additional purification on the next stage (125 mg, 75%).

HRMS (ESI): calculated for [C_53_H_69_N_8_O_18_]^+^ = 1105.4729, found [M+H]^+^ = 1105.4456, R_f_ (CHCl_3_:MeOH:HCOOH 5:1:0.3) = 0.4, R_t_ (System A4) = 16.9 min.

2nd step

The 1,3,6′,3″-Tetra-*N*-Cbz-6″-(2-guanidinoethylamino)-6″-deoxykanamycin A (125 mg, 0.113 mmol) was dissolved in CH_3_OH (4 mL), then 5% Pd/C (190 mg) was added and the reaction mixture was acidified to pH 3 by the addition of CH_3_COOH. The reaction mixture was vigorously stirred at rt at H_2_ flow (1 atm) for 4 h. The catalyst was filtered off via a celite layer; the cake was washed with CH_3_OH (5 mL) and H_2_O (5 mL), and the combined filtrate was concentrated in a vacuum. The target compound was precipitated by the addition of acetone (10 mL), filtered off and dried in a vacuum over P_2_O_5_, resulting in compound **11** (83 mg, 85% yield) as a white solid.

Anal. calculated for [C_21_H_44_N_8_O_10_×5AcOH×6H_2_O]: C, 38.11; H, 7.84; N, 11.47, O 42.58. Found: C, 38.11; H, 7.84; N, 11.47.

HRMS (ESI): calculated for [C_21_H_45_N_8_O_10_]^+^ = 569.3259, found [M+H]^+^ = 569.3467, R_f_ (NH_4_OH:iPrOH 1:1) = 0.28, T_mp_ = 202 °C (decomp.).

The assignment of the signals in the ^1^H and ^13^C spectra is presented in Table S1.

6″-(2-Amino-4,5-dihydro-1H-imidazol-1-yl)-1,3,6′,3″-tetra-N-Cbz-6″-deoxykanamycin A trifluoroacetate (**12**)

EDIA (120 µL, 0.66 mmol) was added to a mixture of 6″-(2-aminoethylamino)-1,3,6′,3″-tetra-*N*-Cbz-6″-deoxykanamycin A (**6a**) (234 mg, 0.221 mmol) and *tert*-butyl-(((*tert*-butyloxycarbonyl)amino)(1*H*-pyrazol-1-yl)methylen) carbamate (137 mg, 0.441 mmol) in DMF (4.5 mL). The reaction mixture was stirred at 50 °C for 24 h, then Et_2_O (150 mL) was added. The forming precipitate was filtered off, washed with Et_2_O (5 × 10 mL) and dried in a vacuum over P_2_O_5_. The resulting intermediate was further dissolved in CH_2_Cl_2_ (2 mL) and TFA (0.2 mL, 2.75 mmol) was added dropwise. The reaction mixture was stirred at rt for 24 h and then evaporated to dryness in a vacuum. The target compounds were purified by column chromatography on silica gel. The elution was carried out by the mixture CHCl_3_:MeOH:HCOOH (10:1.5:0.3). The fractions which contained the individual compounds were combined and evaporated to dryness, resulting in trifluoroacetate of 1,3,6′,3″-tetra-*N*-Cbz-6″-(2-guanidinoethylamino)-6″-deoxykanamycin A (white solid, 25 mg, 25%) and compound **12** in the form of trifluoroacetate (white solid, 20 mg, 23%).

Derivative **12**. HRMS (ESI): calculated for [C_53_H_66_N_7_O_18_]^+^ = 1088.4386, found [M+H]^+^ = 1088.4459, R_f_ (CHCl_3_:MeOH:HCOOH 5:1:0.5) = 0.41, R_t_ (System A4)= 19.1 min.

^1^H NMR (δ, ppm, J/Hz): 1.50–3.59 (m, 7H, cHex), 3.06–4.98 (m, 7H, CH_1″-6″_), 3.09–4.86 (m, 7H, CH_1′-6′_), 3.47 (s, 2H, -NH-CH_2_-CH_2_-), 3.65 (s, 1H, -NH-CH_2_-CH_2_-), 3.72 (s, 1H), (s, 1H, -NH-CH_2_-CH_2_-), 4.45–5.1 (m, 8H, -OCH_2_), 6.80 (t, 1H, 3J = 4.8 Hz, -NH-CH_2 CH6′_-), 7.07 (d, 1H, ^3^J = 9.2 Hz, -NH-CH_2 CH3″_-), 7.17 (d, 1H, ^3^J = 8.2 Hz, -NH-CH_cHex1_-), 7.25–7.40 (m, 20H, 4×Ph), 7.36 (s, 1H, -NH-CH_cHex3_-), 7.55 (s, 1H, -NH-CH_2_-CH_2_-), 7.71 (s, 1H, -C-NH_2_).

^13^C NMR (δ, ppm, J/Hz): 34.3, 40.6, 41.5, 45.8, 48.5, 49.6, 50.4, 56.2, 65.1, 65.3, 69.3, 69.7, 70.0, 70.5, 70.7, 72.6, 73.0, 74.4, 79.8, 85.1, 97.9, 101.5, 127.6, 128.2, 136.9, 137.1, 137.2, 156.5, 156.6, 157.6, 157.9, 158.8.

### 2.2. Biology

#### 2.2.1. Microorganisms

The microbial strains were obtained from the working collection of Gause Institute of New Antibiotics (GINA). Control strains of microorganisms included in the study: *S. aureus* ATCC 29213, *E. coli* ATCC 25922, *P. aeruginosa* ATCC 27853 and *M. smegmatis* ATCC 607. Microbial cultures were preserved at a low temperature (−75 °C) in trypticase-soy broth (Becton, Dickinson, France) with 10–15% glycerol added. Storage under these conditions was performed according to CLSI recommendations [27]. Before the experiment, bacterial strains were activated after cryopreservation by seeding on trypticase-soy agar medium (Beckton, Dickinson, France) and incubated at (36 ± 1) °C for 16–24 h. Individual morphologically homogeneous colonies were suspended in sterile physiological solution, and the turbidity of the suspension was set to 0.5 units according to the McFarland standard on the DEN-1 device (Biosan, Riga, Latvia), which corresponds to 1.5 × 10^8^ CFU/mL for bacterial cultures.

#### 2.2.2. Sample Preparation

Samples were dissolved in sterile distilled water or dimethyl sulfoxide (DMSO), according to the physicochemical characteristic of the samples studied, to a concentration of 10.000 µg/mL. To obtain working solutions, dilutions ranging from 64–0.5 μg/mL were performed.

#### 2.2.3. Assay Setting

Activity was assessed using minimum inhibitory concentration (MIC) values by the microdilution method in Mueller–Hinton Broth (Beckton and Dickinson, France), according to the procedure recommended for determining the sensitivities of microorganisms to antimicrobial agents [27].

The analysis was performed in 96-well plates (Medpolymer, Moscow, Russia). The inoculum was introduced no later than 15 min after preparation. MIC values were analyzed after 15–18 h of incubation at (36 ± 1) °C. The growth of microorganisms in the presence of the tested samples was compared with the growth control without exposure. MIC was determined by the lowest concentration suppressing the visible growth of microorganisms.

The criterion for the accuracy of the obtained results was the control antibiotic kanamycin A and standard strains: *S. aureus* ATCC 29213, *E. coli* ATCC 25922, for which the MIC values were determined: *S. aureus* ATCC 29213—1–4 µg/mL; *E. coli* ATCC 25922–1–4 µg/mL. The MICs of the reference strains must not exceed the confidence limits given in [27], provided that the assay conditions are standard.

#### 2.2.4. Determination of Antibiotic Activity by the Agar-Diffusion Method

On Petri dishes with solid LB-agar medium (1.5%), an overnight culture of the *E. coli* strain JW5503 [28], and the same strain with a deleted kanamycin resistance cassette removed according to the method described in the publication [29], was applied. After drying, 1 µL of antibiotic at a concentration of 50 mg/mL was applied to Petri dishes and incubated at 37 °C for 18 h. Then, Petri dishes were scanned on a ChemiDoc device (Bio-Rad, Hercules, CA, USA) in channels Cy2, Cy3, Cy5. Antibiotic activity was qualitatively determined by the size of the zone of inhibition.

#### 2.2.5. Determination of the Minimum Inhibitory Concentration

Testing was carried out on 2 strains: *E. coli* JW5503 [28] with a removed kanamycin resistance cassette using the method described in the publication [29] and a strain selected from the above by in vivo selection on kanamycin with substitution P610T. Totals of 200 μL (in rows 2–11) and 400 μL (in the first row) of LB liquid medium containing an overnight culture of 3 strains: JW5503 [28] with a deleted kanamycin resistance cassette, diluted 1000-fold, were added to each well of a 96-well 2 mL plate. Then, 2 μL of 50 μg/mL antibiotic was added to each well of the first row. After that, successive dilutions were carried out within one horizontal row, with the antibiotic concentration decreasing by a factor of 2 at each step. The last 2 rows served as controls: row 12 was the original LB medium without cells and antibiotic, row 11 was the cells without antibiotic. After culturing for 18 h at 37 °C and constant agitation, cells were transferred to a low transparent 96-well plate and optical density A590 was measured using a Victor X5 2030 spectrofluorimeter (Perkin Elmer, Waltham, MA, USA). The concentration of the antibiotic in the first well in the dilution series in which the cells did not grow was the minimum inhibitory concentration. The experiment was repeated three times for each antibiotic.

#### 2.2.6. Determination of Translation Accuracy Using Reporters

To qualitatively determine the accuracy of translation in the presence of antibiotics, the *E. coli* strain JW5503 [28], with a kanamycin resistance cassette removed according to the method described in the publication [29], transformed with pJC27 plasmids, on which the β-galactosidase gene encoded, containing an error in the enzyme active center (E537) [30]: GAA→GAC, and a control without replacement, were used. Thus, active β-galactosidase could be synthesized only in the case of translation error.

Petri dishes (10 cm) with solid medium (20 mL) LB-agar (1.5%) containing chloramphenicol at concentrations of 11.3 μg/mL and 80 μg/mL X-Gal were prepared. A mixture of 3.5 mL of cooled LB-agar medium (0.6%) containing 11.3 μg/mL chloramphenicol and 0.5 mL of liquid cell culture A600~1.0 was applied to these Petri dishes, then the dishes were left until solidified. From 1 to 2 μL of antibiotics at concentrations of 50 mg/mL were applied to the resulting two-layer cell culture mediums and incubated at 37 °C for 18 h. The results were documented using a Samsung Galaxy Tab A71 phone camera. The occurrence of blue staining of indigo, a product of the degradation of the X-Gal substrate by the enzyme, along the edge of the inhibition zone indicates that the antibiotic causes translation errors.

## 3. Results

### 3.1. Chemistry

For the synthesis of a series of new 6″-modified KANA derivatives, we employed the common strategy which includes two synthetic steps: (1) the protection of all amino groups with *tert*-butyloxycarbonyl (Boc) or benzyloxycarbonyl (Cbz) groups, followed by (2) the activation of the primary 6″-hydroxyl group by introducing a good leaving group, i.e., triisopropylbenzenesulfonyl (TIBS).

**Scheme 1:**
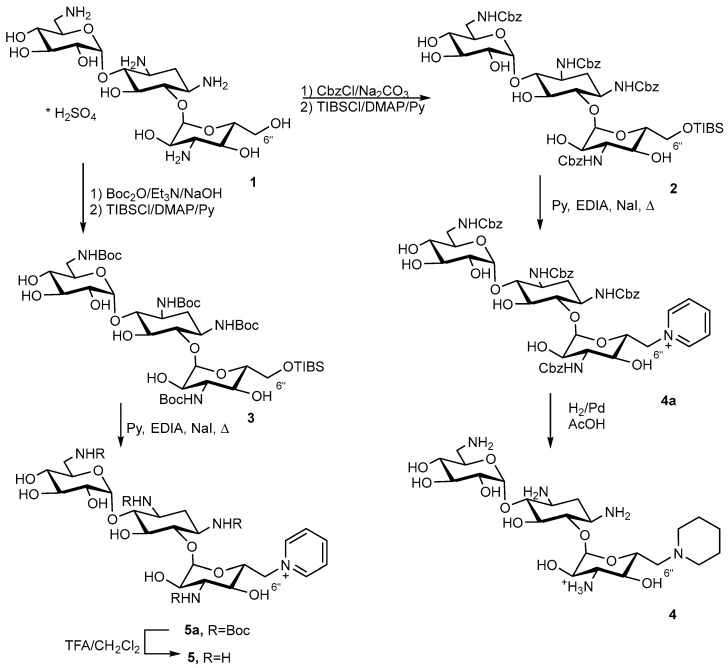
Synthesis of 6″-modified kanamycin A derivatives **4**, **5** (*corresponding salt, Δ reflux).

**Scheme 2:**
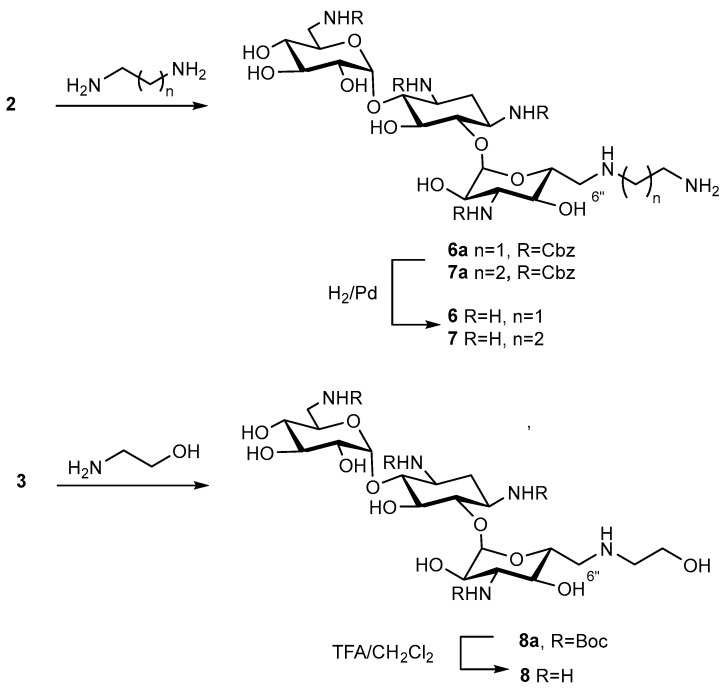
Synthesis of 6″-modified KANA derivatives **6**–**8**.

The syntheses of the 6″-substituted kanamycin derivatives **4**–**8** were performed starting from the commercially available monosulfate of KANA (**1**) (Scheme 1 and Scheme 2). Tetra-*N*-Cbz-6″-*O*-(2,4,6-triisopropylbenzenesulfonyl)kanamycin A (**2**) was obtained in two steps [31]. First, the amino groups of kanamycin A were protected by Cbz groups by the reaction with CbzCl in the presence of Na_2_CO_3_ (Scheme 1). Due to the fact that the reaction of tetra-*N*-Cbz-kanamycin A with *p*-toluenesulfonyl chloride was not selective, the sterically hindered sulfonating agent, 2,4,6-triisopropylbenzenesulfonyl chloride (TIBSCl), was used. The reaction was carried out at room temperature in pyridine, resulting in compound **2** in an 87% yield for two steps.

Tetra-*N*-Boc-6″-*O*-(2,4,6-triisopropylbenzenesulfonyl)kanamycin A (**3**) was obtained by a similar procedure: first, the amino groups of KANA were protected by Boc-groups by the reaction with Boc_2_O in the presence of NaOH, allowing for an increase in the yield of the target intermediate up to 62% in comparison with the previously described 50% for the two-stages procedure (via the obtaining of the KANA-free base and an introduction of the protective groups) [32]. The reaction of tetra-*N*-Boc-kanamycin A with TIBSCl was carried out at room temperature in pyridine resulting in compound **3** in a 39% yield after a chromatographic purification [33].

Next, the interaction of tetra-*N*-protected 6″-*O*-(2,4,6-triisopropylbenzenesulfonyl)kanamycin A (**2** or **3**) with different *N*-nucleophiles was evaluated. Initially, pyridine was used as a solvent for this reaction; however, the elucidation of the structures of the obtained products by the NMR and ESI MS methods revealed that the main reaction products corresponded to the 1,3,6′,3″-tetra-*N*-Cbz-6″-(pyridine-1-ium)-6″-deoxykanamycin A (**4a**) and 1,3,6′,3″-tetra-*N*-Boc-6″-(pyridine-1-ium)-6″-deoxykanamycin A (**5a**) which were isolated in 98% and 71% yields, respectively. However, the classic method of deprotection of the Cbz-group by hydrogenolysis resulted in the simultaneous reduction of the pyridine ring, and 6″-(piperidin-1-yl)-6″-deoxykanamycin A (**4**) was isolated in the form of acetate in 58% yield. The removal of the *N*-Boc-protective groups by the treatment with trifluoroacetic acid (TFA) proceeded smoothly and resulted in the target 6″-(pyridine-1-ium)-6″-deoxykanamycin A (**5**) which was obtained as trifluoroacetate in a 63% yield.

The next step of the KANA modification involved the reaction of the Cbz-protected intermediate (**2**) with an excess of diamines (ethane-1,2-diamine or propane-1,3-diamine), followed by the deprotection of Cbz-groups by hydrogenolisys (Scheme 2). Corresponding 6″-(2-aminoethylamino)-6″-deoxykanamycin A (**6**) and 6″-(3-aminopropylamino)-6″-deoxykanamycin A (**7**) were isolated as acetates in 63% and 77% yields, respectively. The 6″-(2-hydroxyethylamino)-6″-deoxykanamycin A (**8**) was obtained analogously by the interaction of tetra-*N*-Boc-6″-*O*-(2,4,6-triisopropylbenzenesulfonyl)kanamycin A (**3**) with ethanolamine, followed by Boc-deprotection by the treatment with TFA.

**Scheme 3:**
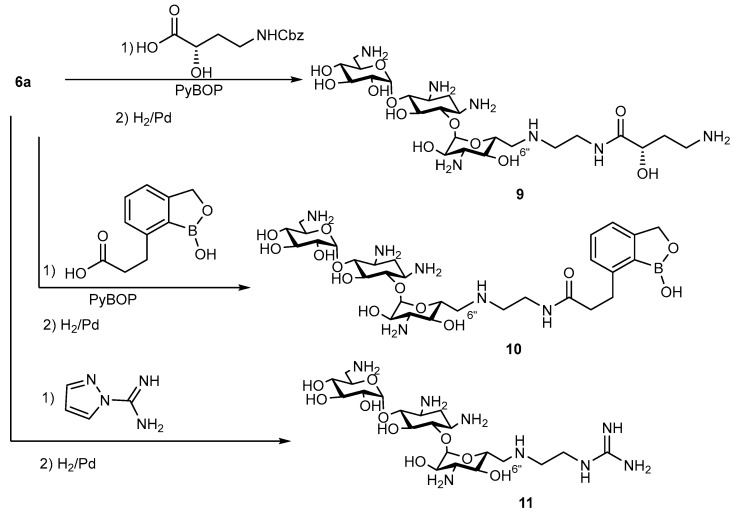
Modification of tetra-N-Cbz-protected 6″-(2-aminoethylamino)-6″-deoxykanamycin A (**6a**).

Tetra-*N*-Cbz-protected 6″-(2-aminoethylamino)-6″-deoxykanamycin A (**6a**) was used for the further modification of the primary amino group of the 2-aminoethyl residue (Scheme 3).

The 6″-((*S*)-7-((2-(4-amino-2-hydroxybutanamido)ethyl)amino))- 6″-deoxykanamycin A (**9**) was obtained by the acylation of the intermediate **6**a with (*S*)-4-(((benzyloxy)carbonyl)amino)-2-hydroxybutanoic acid [34] in the presence of the condensing agent benzotriazol-1-yloxytripyrrolidinophosphonium hexafluorophosphate (PyBop)) followed by the amino groups’ deprotection by hydrogenolysis (Scheme 3). Compound **9** was isolated in a 61% yield for two steps.

The organoboron moiety is one of the promising pharmacophores in medicinal chemistry [35], and we aimed to investigate the effect of this modification on the pharmacological activity of the natural AGs. As such, a congener **10** in which KANA conjugated with benzoxaborole residue was prepared by the acylation of the intermediate **6a** with 3-(1-hydroxy-1,3-dihydrobenzo[c][1,2]oxaborol-7-yl)propionic acid in the presence of PyBop reagent followed by the Cbz-groups cleavage by hydrogenolysis. The target derivative **10** was isolated as an acetate in a 27% yield counting for two stages.

**Scheme 4:**
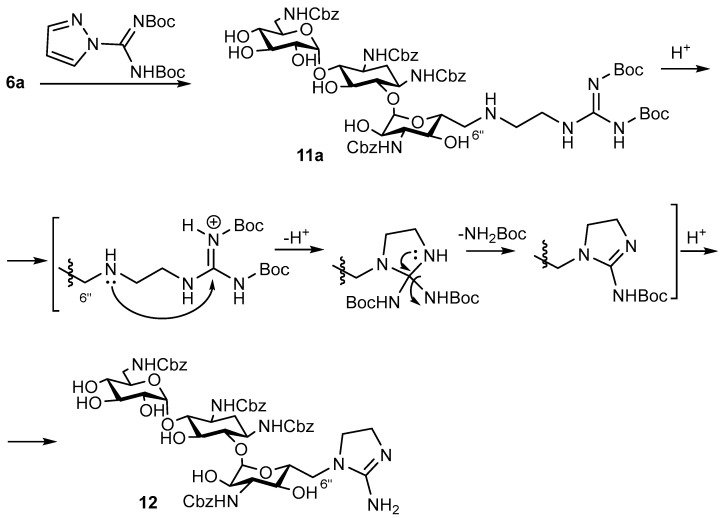
Interaction of tetra-*N*-Cbz-protected 6″-(2-aminoethylamino)-6″-deoxykanamycin A (**6a**) with *tert*-butyl-((Boc)amino)(1*H*-pyrazol-1-yl)methylene)carbamate followed by N-Boc-deprotection.

The 6″-(2-Guanidinoethylamino)-6″-deoxykanamycin A (**11**) was obtained by the guanidation of the intermediate **6a** with 1*H*-pyrazole-1-carboxamide followed by deprotection in 64% yield (Scheme 3). Initially, the di-Boc-derivative of 1*H*-pyrazole-1-carboxamide (*tert*-butyl-((Boc)amino)(1*H*-pyrazol-1-yl)methylene) carbamate [36]) was employed for the synthesis of the target compound **11** (Scheme 4), but the Boc-group cleavage from the intermediate **11a** by the treatment with TFA led to the formation of a mixture of products. The obtained mixture was further separated by the column chromatography method, resulting in the two main compounds which were analyzed by physico-chemical and spectral methods. Along with the target Cbz-protected guanidine derivative **11,** the side imidazoline derivative **12** was isolated from the reaction mixture (Scheme 4). The structure of compound **12** was elucidated using mass spectrometry and NMR spectroscopy data analysis.

The ^1^H NMR spectrum of compound **12** does not reveal the signals of the Boc-group as well as the signals of NH groups of the guanidine residue. A cyclic structure of the side 6″-group in derivative **12** was proposed based on the ^1^H-^13^C HMBC and ^1^H-^15^N correlation experiments on direct spin–spin interaction constants with multiplicity edition (^1^H-^15^N edHSQC) data (Figures S29 and S30 in the Supporting Information File). A correlation between the signals of the H-6 protons of the 3,6-dideoxy-3,6-diaminoglucose residue and the signal of carbon of the guanidine residue at 158.9 ppm was observed in the HMBC spectrum of compound **12** (See Supporting Information File). This interaction is only possible if the carbon atom of guanidine is located equidistant from both CH_2_ groups and this fragment has a cyclic (imidazoline) structure.

Based on these data, the structure of the obtained side product **12** corresponds to 6″-(2-amino-4,5-dihydro-1*H*-imidazol-1-yl)-6″-deoxykanamycin A (Scheme 4). The suggested structure of compound **12** was also supported by ^1^H and ^1^H-^15^N edHSQC NMR spectroscopy data. A broad peak at 7.71 ppm with the integral intensity of two proton units corresponding to the amino group was observed in the ^1^H NMR spectrum of derivative **12**. The ^1^H-^15^N HSQC correlation spectrum also reveals the signal of the NH_2_ group, with a negative polarity at −311.2 ppm (See Supporting Information File) along with the signals of the NH amide protons of the Cbz-protecting groups.

HR ESI mass spectrum has the only monoisotopic peak at 1088.4459 Da, which is in complete agreement with the proposed structure of compound **12**. However, the isolated yield of compound **12** was low (~20%), and we were not able to obtain the corresponding deprotected derivative in the amounts sufficient for further biological studies.

The purity of the intermediate and target compounds **4**–**11** was confirmed by the TLC and/or HPLC methods (for UV-absorbing derivatives) and by the elemental analysis data. Structures were confirmed by the HR ESI mass spectrometry and NMR spectroscopy methods.

### 3.2. In Vitro Antimicrobial Activity Studies

The antibacterial activities of the new KANA derivatives **4**–**11** were evaluated on a panel of G− and G+ bacterial strains in comparison with the parent KANA (**1**) (Table 1).

The results of the screening demonstrated that none of the tested compounds, including KANA (**1**), were active against the *P. aeruginosa* ATCC 27853 strain (MICs > 32 µg/mL). The introduction of the piperidine or pyridinium residues at the 6″-position of KANA (compounds **4** and **5**) resulted in the significant loss of the antibacterial activity against all tested strains, including *Staphylococcus aureus* ATCC 29213 (G+), *E. coli* ATCC 25922 (G−) and *Mycobacterium smegmatis* ATCC 607 (MICs > 64 µg/mL), suggesting that a bulky cyclic residue might disrupt the interaction of the antibiotic with the target. On the contrary, the presence of the diamino group (compounds **6** and **7**) allowed them to retain antibacterial activity against three of the tested strains (MICs~0.5–4 µg/mL vs. MICs~0.125–4 for **1**). However, further modification of the amino group of compound **6** by (*S*)-4-(((benzyloxy)carbonyl)amino)-2-hydroxybutyric acid (compound **9**) or benzoxaborole residue (compound **10**) resulted in a dramatic decrease of the antibacterial activity in comparison with both KANA (**1**) and compound **6** (MICs~8.5–64 µg/mL for **9** and **10** vs. MICs~0.125–4 for KANA and MICs~1–4 for compound **6**).

The most active among the series of KANA analogues was the guanidine derivative **11**, that demonstrated two-times-less activity in comparison with the parent kanamycin A (**1**) against *E. coli* ATCC 25922 strain, similar activity against *M. smegmatis* ATCC 607 strain, and was four-times more active than kanamycin A (**1**) against the *S. aureus* ATCC 29213 strain (MICs values zero-point-five and two for compound **11** and KANA, respectively).

Next, we checked if the new derivatives are “sensitive” to the most common mechanism of resistance to aminoglycoside antibiotics, which is based on the work of the aminoglycoside-modifying enzymes (AMEs) [37]. An in-depth evaluation of the antibacterial activities of compounds **7**, **8** and **11** revealed that they were inactive against the kanamycin A-resistant strains *Proteus mirabilis* ESBL 137, *Klebsiella pneumoniae* 1951, *K. pneumoniae* ESBL 126, *E. coli* ESBL 135, *Methicillin resistant S. aureus* 88 (MRSA) and *Staphylococcus epidermidis* C 2001MR (MICs > 64 µg/mL, data not shown). Inactivity against *P. aeruginosa* ATCC 27853 (Table 1) could be explained by the presence of the aminoglycoside-3′-phosphotransferase gene in the genome [38].

An evaluation of the antibacterial activity of the KANA derivatives **4**, **6**–**11** by the diffusion-in-agar method on *E. coli* BW25113 Δ*tolC* and the same strain bearing the aminoglycoside-3′-phosphotransferase gene on a plasmid revealed that tested compounds **4**, **6**–**11** demonstrated significantly lower potencies against the resistant strain, not surprisingly suggesting that they were substrates for the aminoglycoside-3′-phosphotransferase (Figure S31).

Although the inactivation of the AGs by the modifying enzymes is the most common resistance mechanism, mutations in the gene *fusA* (EF-G) can also be responsible for the resistance to aminoglycosides; P610T EF-G mutations were reported to be distributed in clinically important strains [39,40].

Next, we tested the activity of KANA derivatives **4**, **6**–**11** against the *fusA* mutant strain with the substitution P610T. Although the tested compounds **4, 6**–**11** in general demonstrated lower antibacterial activities (Table 2) in comparison with KANA (**1**), the difference between MICs values against the wild type and the resistant strains was less significant for 6″-modified KANA derivatives than for the parent antibiotic: the MIC/MIC (WT) ratio for the tested compounds **4**, **6**–**11** was **1**–**4** vs. **6**–**12** for KANA, respectively. Thus, one can hypothesize that 6″-modified compounds are less affected by the resistance that occurs as a mutation in the gene of the elongation factor G.

**Figure 3:**
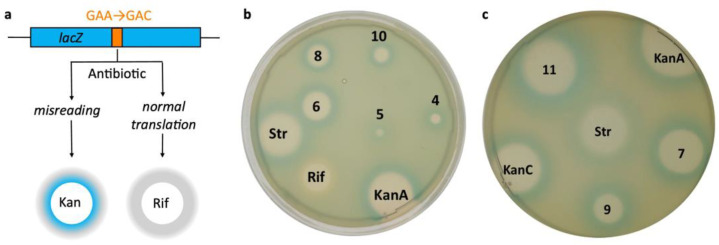
Kanamycin and its derivatives increase the misreading of 537 codon GAC (Asp) of the β-galactosidase gene by GlutRNA. (**a**)—schematic depicting the principle of the method. If an antibiotic causes decoding errors, GAC is decoded as GAA, a β-galactosidase is synthesized which cleaves the X-Gal substrate, causing indigo blue staining. Otherwise, no functional protein is formed. (**b**,**c**)—results of the test for compounds **4**–**11**. KANA—Kanamycin A; KanC—Kanamycin C; Str—Streptomycin; Rif—Rifampicin. Concentration of **4**–**11**, KANA (**c**), KanC, Rif—50 mg/mL; Str, KANA (**b**)—5 mg/mL. Volume of each antibiotic 1 µL. Streptomycin and kanamycins are miscoding inductors, rifampicin affects RNA synthesis and does not induce ribosomal errors. An active β-galactosidase is synthesized only in the case of a translation error, and if the mechanism of action of the antibiotic is associated with the miscoding, we observe indigo blue staining along the edge of the inhibition zone due to the presence of a degradation product of the X-Gal substrate. Figure 3 shows that all tested analogs **4**, **6**–**11** caused a blue coloring of the bacterial lawn, which specifies translation errors in the targeted cells. Thus, according to this data, modifications of the 6″ position of kanamycin A do not prevent the induction of translation errors.

As the mechanism of the antibacterial action of AG is based on the misreading of mRNA and the incorporation of the incorrect amino acids during protein synthesis, our next step included an evaluation of the action of new KANA derivatives on the *E. coli* BW25113 reporter strain which contains the β-galactosidase gene with a mutation in the enzyme active (catalytic) site E537 [30] (Figure 3).

## 4. Discussion

Aminoglycoside antibiotics, cidal inhibitors of bacterial protein synthesis, have experienced a renaissance during the last two decades. The development of plazomicin, the first new AG approved by the FDA in nearly 40 years, is an example and confirmation of the renewed interest in this “old” class of antibiotics. According to the recent scoping review, plazomicin seems to be a powerful, practical and safe tool for the control and resolution of infections that would not respond with favorable outcomes to the use of classic broad-spectrum antibiotics, but this new antimicrobial drug, to be properly exploited and have its effectiveness maintained over time, should be administered only when necessary, and sparingly [41].

The expansion of the aminoglycoside antibiotic class might help to fill an urgent unmet need in the new antimicrobial drugs effective against the ever-expanding spread of multidrug-resistant pathogens, especially G-negative bacteria [42].

Aiming at finding new active AG derivatives, we studied the modification of the 6″-position of KANA resulting in a series of 6″-deoxykanamycin A analogues bearing an additional basic group that could be ionized at physiological pH (amino-, guanidino or pyridinium).

For the first time, we demonstrated the ability of tetra-*N*-(Boc or Cbz-protected)-6″-*O*-(2,4,6-triisopropylbenzenesulfonyl)kanamycin A to interact with a weak nucleophile such as pyridine, resulting in the formation of the corresponding pyridinium derivative. However, such a modification turned out to be unfavorable for the antibacterial activity of the target compound obtained after the amino group’s deprotection. The introduction of the small diamino residues at the 6″-position of KANA did not significantly alter the antibacterial activity of the parent antibiotic; nevertheless, further modification by the acylation reaction resulted in a complete loss of potency, while the introduction of the guanidine residue led to the compound **11** with an improved activity against the *S. aureus* ATCC 29213 strain. Unsurprisingly, the modification of KANA at the 6″-position does not help overcome resistance caused by aminoglycoside-3′-phosphotransferase, i.e., studied transformations of the molecule most likely do not prevent the antibiotic from being phosphorylated at 3′-hydroxy group [38].

Interestingly, we observed that the difference in susceptibility between the *E. coli* ATCC 25922 strain and the BW25113 *E. coli* strain with the Δ*tolC* knockout is less pronounced for KANA (~2 times) and increased significantly for its new analogs **4**, **6**–**11** (up to 15 times in the case of compound **6**). While the ribosomes and translation factors of both strains are almost identical, the discovered difference could appear because of the difference in the lipopolysaccharides (LPS) and cell wall structures of the outer membranes of the *E. coli* BW25113 ΔtolC and *E. coli* ATCC 25922 strains [43]. Despite their frequent use as therapeutic agents, the mechanisms of aminoglycoside outer-membrane (OM) translocation remain incompletely understood. The self-promoted pathway is a proposed uptake mechanism. Here, divalent cations between LPS molecules are displaced by AGs which leads to brief OM destabilization, thereby enabling OM translocation. LPS—being the major component of the outer leaflet of the OM—plays a central role for the integrity and the selective permeability of the OM [43]. Hence, the difference in activity between the ATCC 25922 and BW25113 *E. coli* strains suggests that the decline might be due to penetration issues, as these strains have different LPS and cell walls, which could provide different sensitivities to antibiotics.

However, the obtained 6″-modified KANA derivatives **4, 6**–**11** were less influenced by the resistant mechanism associated with mutations of the elongation factor G than the parent KANA (**1**), suggesting that this direction of the modification is still prospective for the further investigations. AGs inhibit translocation, and recently it has been demonstrated that they trap ribosomes in the conformation, which is not preferable for EF-G binding [44]. The EF-G mutants (P610T) that possess resistance to aminoglycosides probably could bind to ribosome even in the presence of the aminoglycoside. Synthesized 6″-modified KANA derivatives could trap ribosomes in the conformation, which is not preferable for wild-type and mutant EF-G. Further research on the molecular basis of EF-G mutations in aminoglycoside resistance could shed light on these observations.

## Figures and Tables

**Table 1:** Antibacterial activity of the new obtained kanamycin derivatives **4**–**11** in comparison with the KANA (**1**).

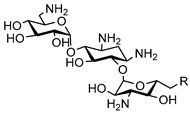					
Compound	R	MIC *, µg/mL			
		*S. aureus* ATCC 29213	*E. coli* ATCC 25922	*P. aeruginosa* ATCC 27853	*M. smegmatis* ATCC 607
**1**	OH	2	2	>64	0.125–0.25
**4**	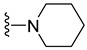	>64	>64	>64	64
**5**	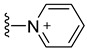	>64	>64	>64	32
**6**	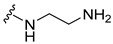	2	4	32	1
**7**	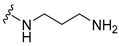	2	8	>64	0.5
**8**	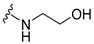	8	32	>64	4
**9**	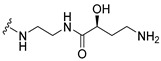	8	32	>64	1
**10**	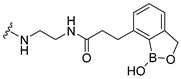	>64	>64	>64	8
**11**	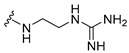	0.5	4	64	0.25

* MIC—minimal inhibitory concentration, MICs are measured as the lowest concentration of agents that prevent any visible growth. The results of the experiments were definitely reproducible. In cases of full coincidence of the data obtained, the MIC is represented as a single number.

**Table 2:** Antibacterial activity of the KANA derivatives **4**, **6**–**11** against *E. coli* BW25113 Δ*tolC* strain and its derivative with mutated *fusA* (P610T).

Compound	*E. coli* BW25113 Δ*tolC*, MIC, µg/mL	*E. coli* BW25113 Δ*tolC* P610T EF-G (P610T), MIC, µg/mL	MIC(P610T)/MIC
KANA (**1**)	5	31	6
Fusidic acid	2	2	1
Erythromycin	2	2	1
**4**	>250	>250	-
**6**	63	125	2
**7**	125	125	1
**8**	125	500	4
**9**	125	250	2
**10**	250	500	2
**11**	31	63	2

## Data Availability

Data are contained within the article and in the Supporting Information File.

## Supplementary Materials

The following supporting information can be downloaded at: https://www.mdpi.com/article/10.3390/pharmaceutics15041177/s1, Table S1: Assignments of the signals in 1H and 13C NMR spectra of compounds **4**–**11**; Figures S1 and S2: 1H and 13C NMR spectra of 1,3,6′,3″-tetra-N-Cbz-kanamycin A; Figures S3 and S4: 1H and 13C NMR spectra of 1,3,6′,3″-tetra-N-Boc-kanamycin A; Figures S5 and S6: 1H and 13C NMR spectra of 1,3,6′,3″-tetra-N-Cbz-6″-O-(2,4,6-triisopropylbenzenesulfonyl)kanamycin A (**2**); Figures S7–S28: 1H and 13C NMR spectra of the kanamycin A derivatives **4a**, **4**–**7**, **8a**, **8**, **9a**, **9**–**11**; Figure S29: Fragment of the HMBC 1H-13C spectrum of compound **12**; Figure S30: 1H-15N edHSQC spectrum of compound **12**; Figure S31: The antibacterial activity of the kanamycin A derivatives **4**, **6**–**11** and reference antibiotics.

## Abbreviations

Boc, *tert*-butyloxycarbonyl; Cbz, benzyloxycarbonyl; d, doublet; dd, doublet of doublets; DMF, dimethylformamide; DMSO, dimethylsulfoxide; HPLC, high performance liquid chromatography; HRMS (ESI), high-resolution mass spectrometry with electrospray ionization; IC_50_, is the amount of a drug which causes the inhibition of growth of the 50% of cells; MIC, minimum inhibitory concentration; NMR, nuclear magnetic resonance; PyBop, benzotriazol-1-yloxypyrrolodinophosphnium hexafluorophosphate; rt, room temperature; s, singlet; TFA, trifluoroacetic acid; TIBS, 2,4,6-triisopropylbenzenesulfonyl; TLC, thin layer chromatography; t, triplet.
