# Supplementary material for: Synthesis of 6″-Modified Kanamycin A Derivatives and Evaluation of Their Antibacterial Properties
Source: Pharmaceutics. 2023 Apr 7;15(4):1177. doi: 10.3390/pharmaceutics15041177 (PMC10141514; doi:10.3390/pharmaceutics15041177)
Supplement: Supplementary file 1 [file pharmaceutics-15-01177-s001.zip › pharmaceutics-2289117-supplementary.pdf]

SUPPORTING INFORMATION

Synthesis of 6”-modified kanamycin A derivatives and evaluation of their antibacterial properties

Shapovalova K.S.[1], Zatonsky G.V.[1], Grammatikova N.E.[1], Osterman I.A.[2, 3], Razumova E.A.[4], Shchekotikhin A.E.[1], Tevyashova A.N.[1*]

[1]Gause Institute of New Antibiotics, 11 B. Pirogovskaya, 119021, Moscow, Russia

[2]Center of Life Sciences, Skolkovo Institute of Science and Technology, Bolshoy Boulevard 30, bld. 1, 121205, Moscow, Russia

[3]Center for Translational Medicine, Sirius University of Science and Technology, Olympic Avenue 1, 354340 Sochi, Russia.

[4]Department of Chemistry, Lomonosov Moscow State University, Leninskie Gory 1, 119991, Moscow, Russia

LIST OF CONTENTS:

Table S1. Assignments of the signals in ^{1}H and ^{13}C NMR spectra of compounds **4–11** S2-S3

Figures S1-S2. ^{1}H and ^{13}C NMR spectra of 1,3,6’,3”-tetra-N-Cbz-kanamycin A S4-S5

Figures S3-S4. ^{1}H and ^{13}C NMR spectra of 1,3,6’,3”-tetra-N-Boc-kanamycin A S6-S7

Figure S5-S6. ^{1}H and ^{13}C NMR spectra of 1,3,6’,3”-tetra-N-Cbz-6”-*O*-(2,4,6-triisopropylbenzenesulfonyl)kanamycin A (**2**) S8-S9

Figures S7-S28. ^{1}H and ^{13}C NMR spectra of the kanamycin A derivatives **4a**, **4–7**, **8a**, **8**, **9a**, **9–11** S10-S31

Figure S29. Fragment of the HMBC ^{1}H-^{13}C spectrum of compound **12** S32

Figure S30. ^{1}H-^{15}N edHSQC spectrum of compound **12** S33

Figure S31. The antibacterial activity of the kanamycin A derivatives **4**, **6–11** and reference antibiotics S34

Table S1. Assignments of the signals in ^{1}H and ^{13}C NMR spectra of compounds **4–11.**

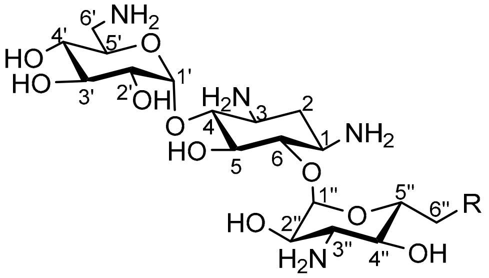

Atom	Compound, $^{13}C/^{1}H$							
	**4**	**5**	**6**	**7**	**8**	**9**	**10**	**11**
1'	101.7	103.5	101.5	102.3	101.7	101.9	101.8	102.0
	5.70	5.09	5.61	5.47	5.66	5.51	5.51	5.55
2'	74.0	71.0	74.0	74.2	73.9	74.2	74.4	74.1
	3.67	3.96	3.65	3.68	3.73	3.66	3.64	3.68
3'	74.9	74.8	75.0	75.1	74.9	75.1	75.4	74.9
	3.80	3.75	3.83	3.78	3.84	3.77	3.78	3.78
4'	73.9	74.1	73.5	73.8	73.6	73.8	73.9	73.7
	3.39	3.37	3.45	3.41	3.46	3.39	3.93	3.40
5'	71.7	71.8	71.5	71.6	71.6	71.6	72.6	71.6
	4.00	3.91	4.08	4.04	4.07	4.03	4.01	4.02
6'	43.4	43.5	43.2	43.2	43.2	43.2	43.6	43.1
	3.48/3.22	3.44/3.19	3.50/3.28	3.43/3.23	3.49/3.30	3.42/3.21	3.34/3.12	3.44/3.24
1	51.6	51.5	51.4	51.7	51.5	51.6	51.8	51.4
	3.37	3.42	3.31	3.12	3.40	3.12	3.02	3.32
2	32.0	30.5	33.3	34.7	32.4	34.8	37.1	32.6
	2.44/1.92	2.47/1.96	2.39/1.74	2.25/1.56	2.46/1.85	2.22/1.54	2.10/1.39	2.40/1.75
3	53.1	53.1	53.2	53.5	53.2	53.5	57.3	53.2
	3.51	3.50	3.50	3.34	3.55	3.31	3.14	3.49
4	86.8	81.4	87.5	87.9	87.0	88.0	89.5	87.2
	3.78	3.79	3.71	3.61	3.83	3.58	3.42	3.73
5	77.7	76.8	76.9	77.2	77.1	77.1	77.2	74.4
	3.84	3.52	3.88	3.78	3.89	3.78	3.74	3.51
6	83.2	86.2	85.0	87.3	84.4	86.9	88.3	85.0
	3.89	3.70	3.72	3.54	3.83	3.53	3.44	3.72
1''	103.2	101.2	103.2	103.1	103.1	103.1	102.9	103.2
	5.21	5.41	5.16	5.11	5.22	5.10	5.11	5.13
2''	71.1	69.9	71.4	72.1	71.1	72.2	73.9	71.1
	3.99	3.63	3.97	3.84	4.03	3.82	3.66	3.95
3''	57.4	57.7	57.8	57.5	57.6	57.5	57.6	57.6
	3.55	3.56	3.51	3.36	3.57	3.33	3.57	3.50
4''	70.7	73.9	70.5	71.4	70.4	71.6	73.6	70.3
	3.60	3.66	3.65	3.50	3.68	3.47	3.32	3.61
5''	69.9	73.9	73.7	71.9	71.0	72.2	72.6	71.9
	4.34	4.36	4.08	4.13	4.29	4.10	4.04	4.15
6''	60.3	64.4	51.3	51.3	50.8	51.3	51.7	51.3
	3.53/3.32	5.04/4.77	3.13/2.95	3.33/3.12	3.60/3.35	3.27/3.06	3.09/2.87	3.39/3.17

	Introduced moiety							
	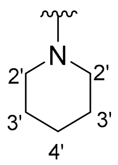	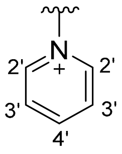	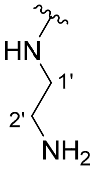	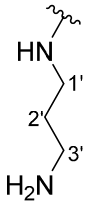	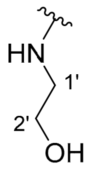	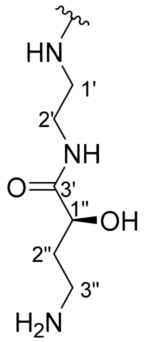	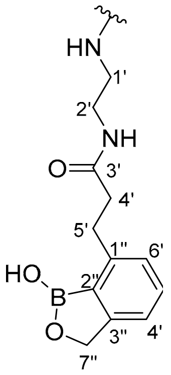	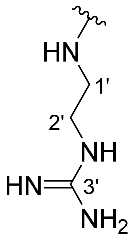
1'			48.6<br>3.07	48.3<br>3.07	52.6<br>3.34	50.3<br>3.08	40.3<br>3.36	49.5<br>3.24
2'	57.0<br>3.30/3.60	148.4<br>8.86	40.8<br>3.21	27.4<br>2.05	59.4<br>3.93	39.6<br>3.55/3.48	50.4<br>2.82	41.1<br>3.56
3'	25.5<br>1.86	131.0<br>8.10		39.8<br>3.10		179.0<br>(C=O)	179.9<br>(C=O)	159.9<br>(C=NH)
4'	23.9<br>1.67	149.3<br>8.61					33.3<br>3.10	
5'							40.5<br>2.70	
1"						72.4<br>4.32	151.5	
2"						33.7<br>2.18/1.98	130.5	
3"						39.6<br>3.16	144.6	
4"							121.8<br>7.13,<br>d, J=7.4	
5"							130.4<br>7.28,<br>t, J=7.3	
6"							128.8<br>7.10,<br>bd, J=7.0	
7"							70.2<br>4.83<br>9.75 (^{11}B)	

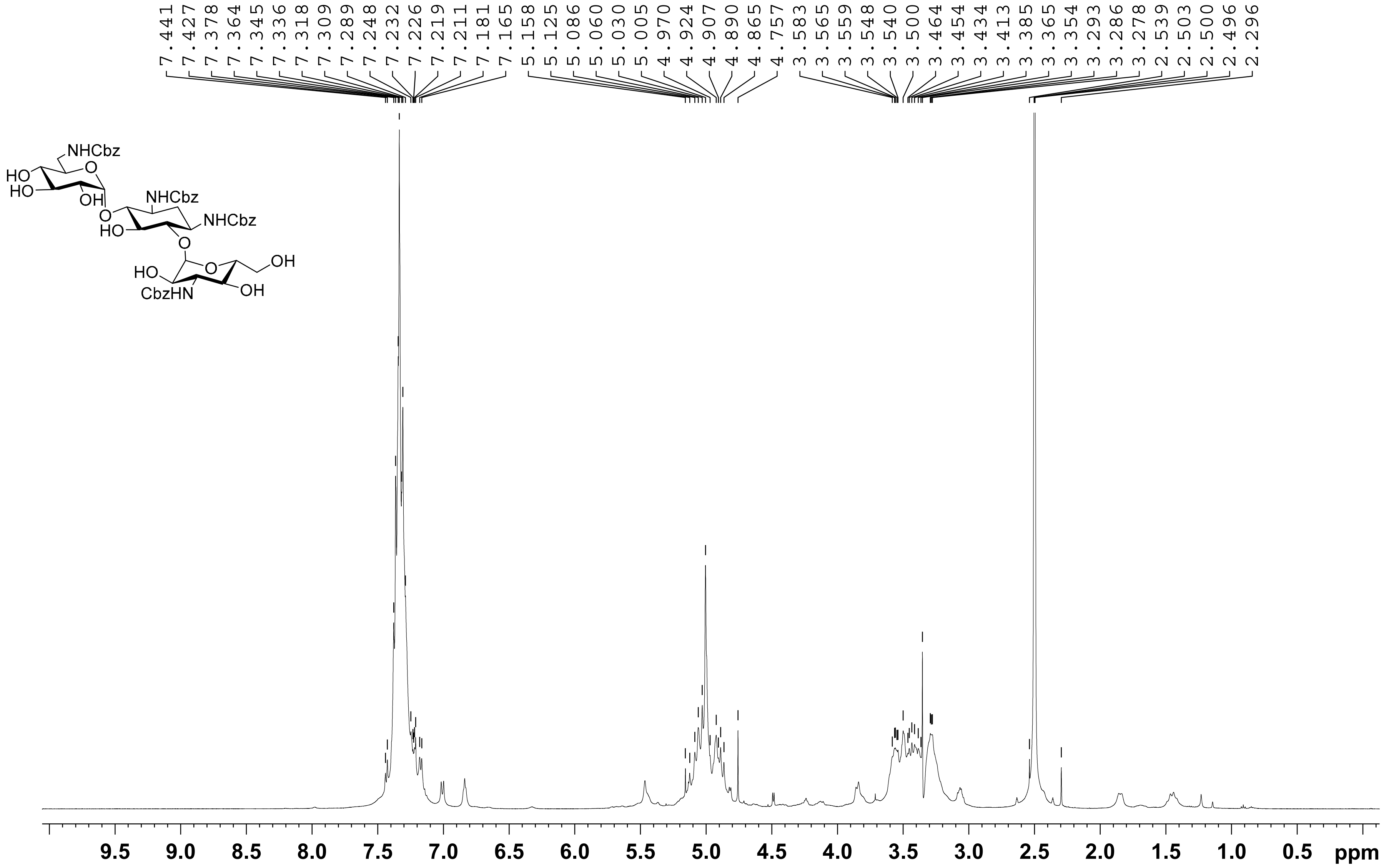

Figure S1. ^{1}H NMR (500.2 MHz, d6-DMSO) spectrum of 1,3,6',3''-tetra-N-Cbz-kanamycin A

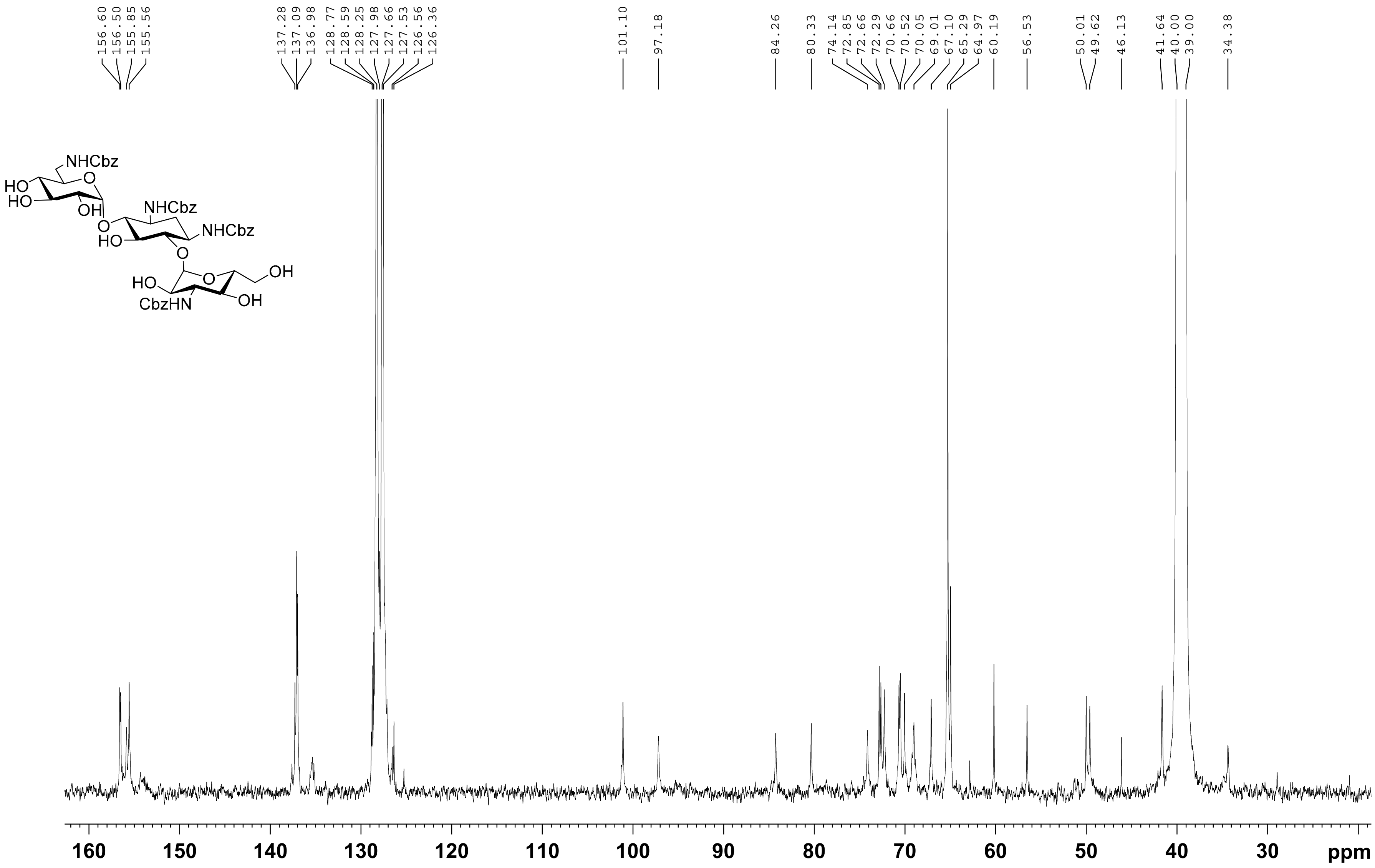

Figure S2. ^{13}C NMR (125.8 MHz, d6-DMSO) spectrum of 1,3,6',3''-tetra-N-Cbz-kanamycin A

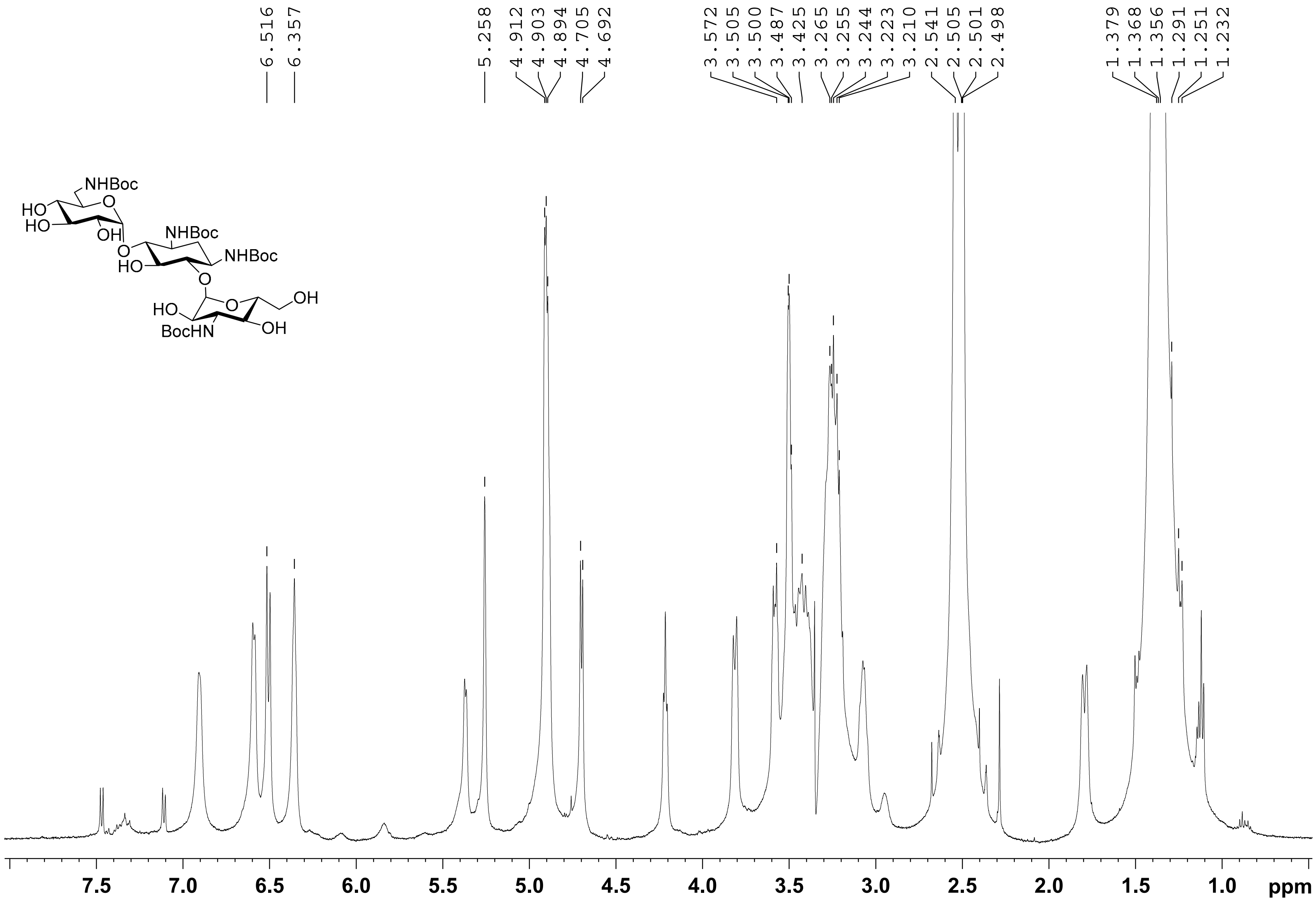

Figure S3. ^{1}H NMR (500.2 MHz, d6-DMSO) spectrum of 1,3,6',3''-tetra-N-Boc-kanamycin A

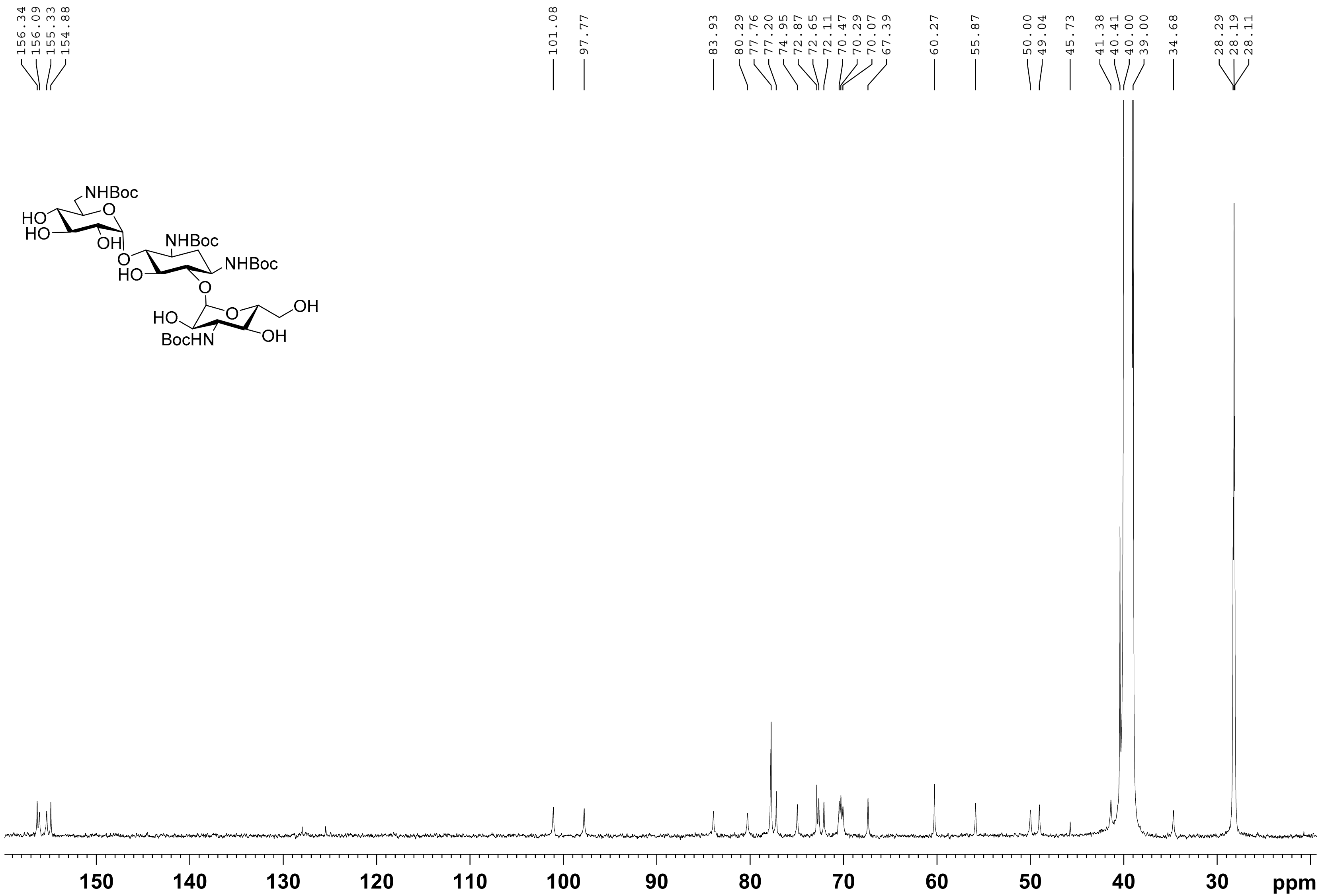

Figure S4. ^{13}C NMR (125.8 MHz, d6-DMSO) spectrum of 1,3,6',3''-tetra-N- Boc-kanamycin A

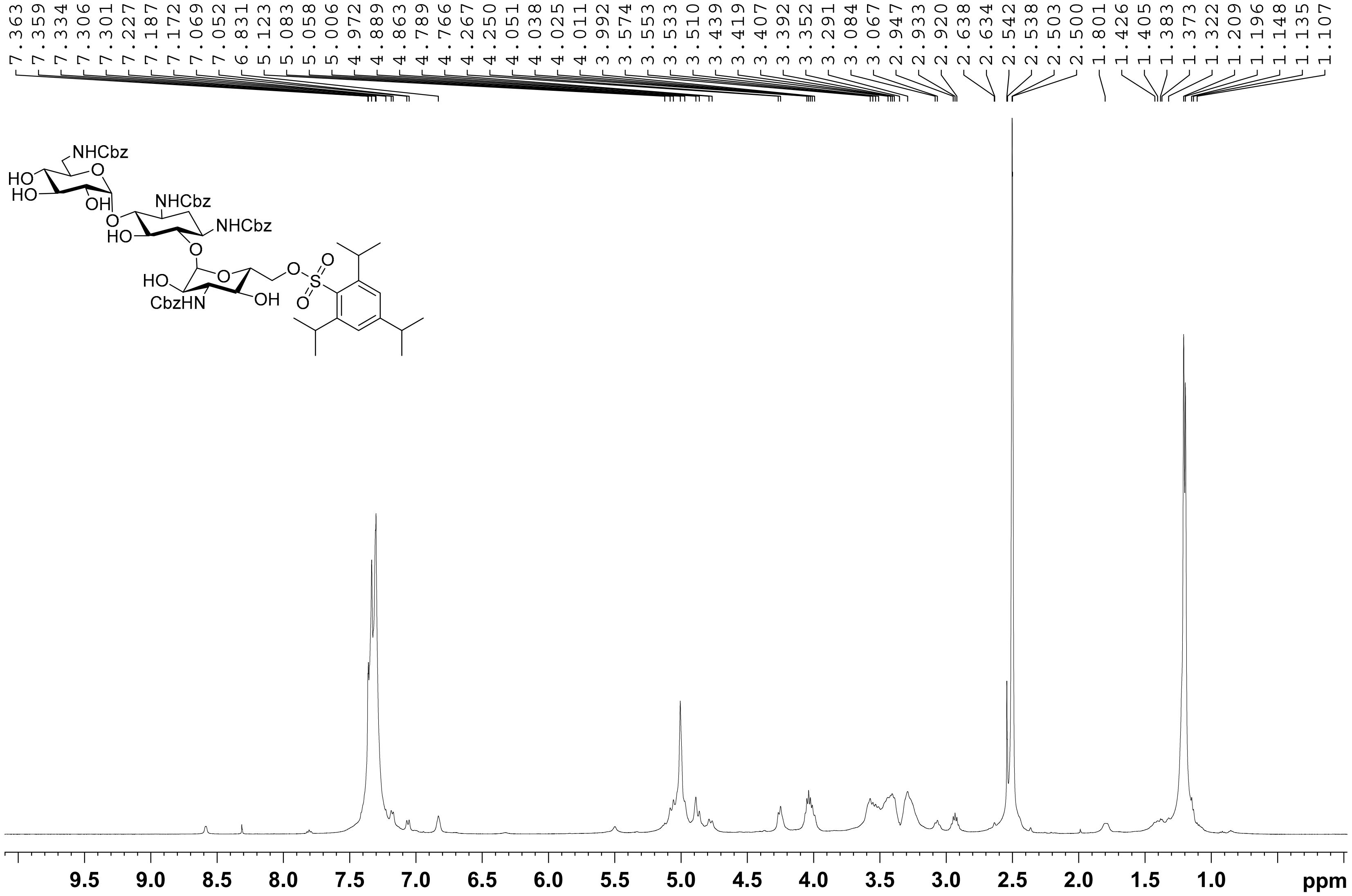

Figure S5. ^{1}H NMR (500.2 MHz, d6-DMSO) spectrum of 1,3,6’,3”-tetra-N-Cbz-6”-*O*-(2,4,6-triisopropylbenzenesulfonyl)kanamycin A (**2**)

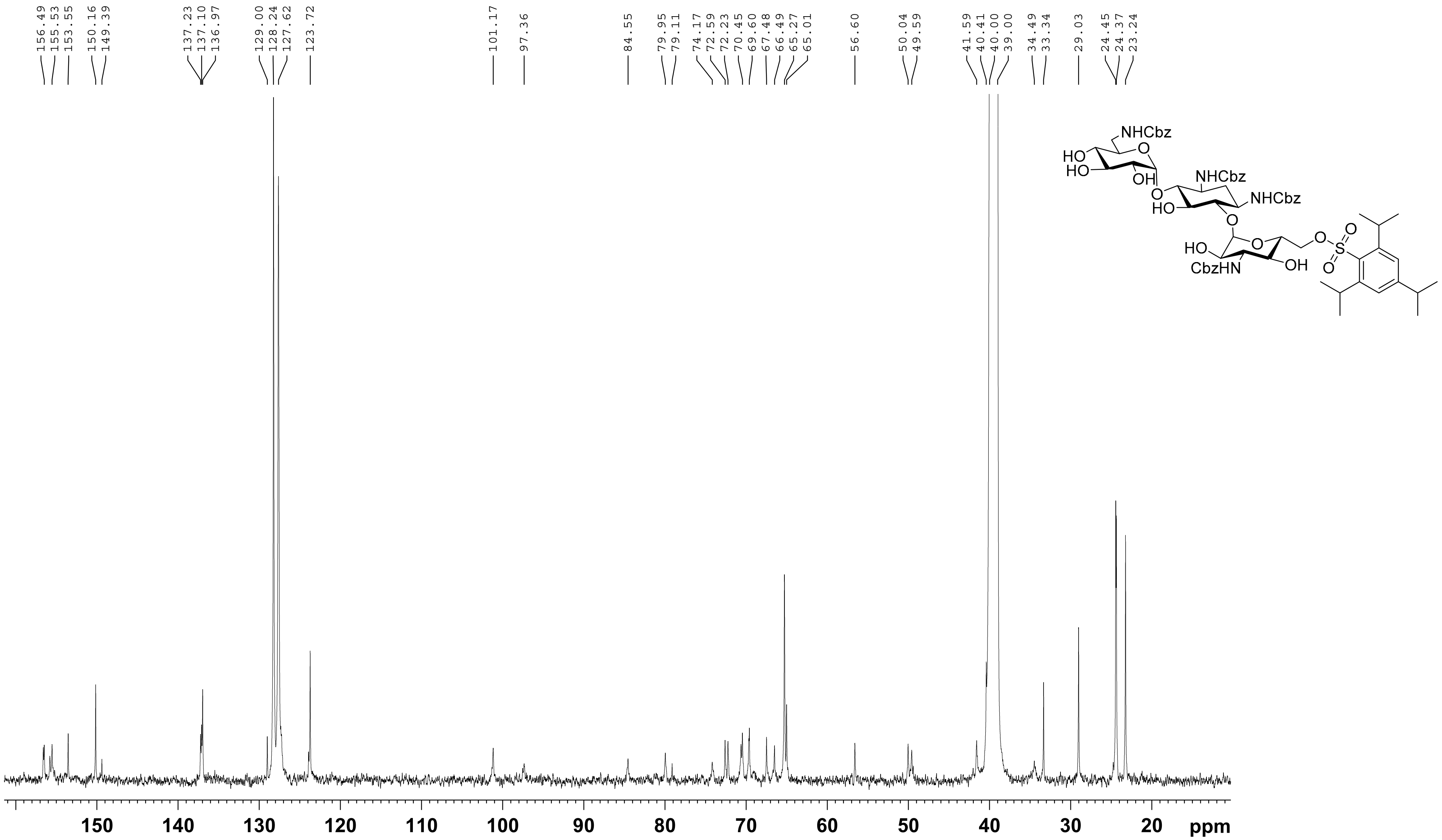

Figure S6. ^{13}C NMR (125.8 MHz, d6-DMSO) spectrum of 1,3,6',3"-tetra-N-Cbz-6"-O-(2,4,6-triisopropylbenzenesulfonyl)kanamycin A (**2**)

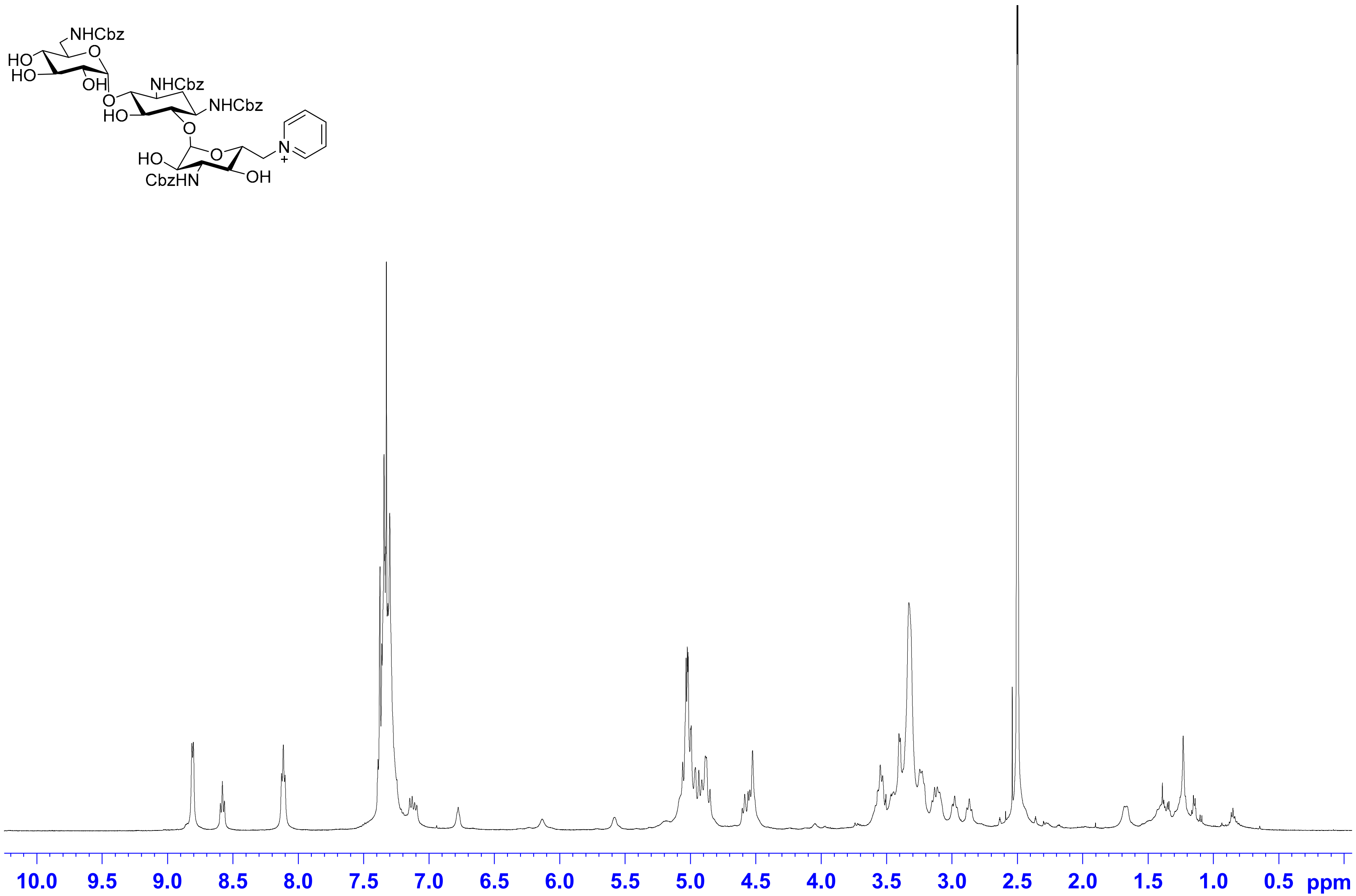

Figure S7. ^{1}H NMR (500.2 MHz, d6-DMSO) spectrum of 6”-(pyridine-1-ium)-1,3,6’,3”-tetra-N-Cbz-6”-deoxykanamycin A (**4a**)

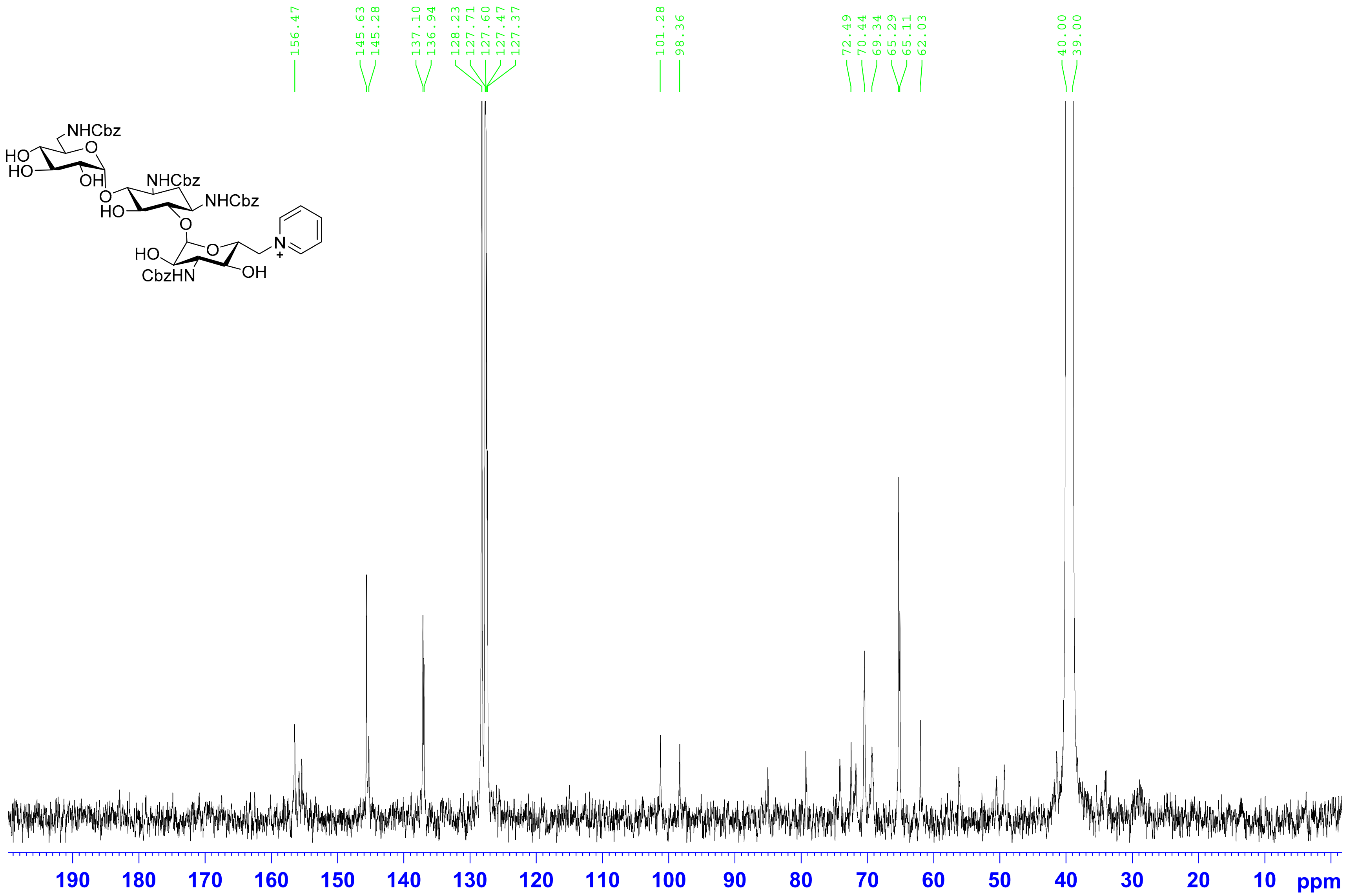

Figure S8. ^{13}C NMR (125.8 MHz, d6-DMSO) spectrum of 6”-(pyridine-1-ium)-1,3,6’,3”-tetra-N-Cbz-6”-deoxykanamycin A (**4a**)

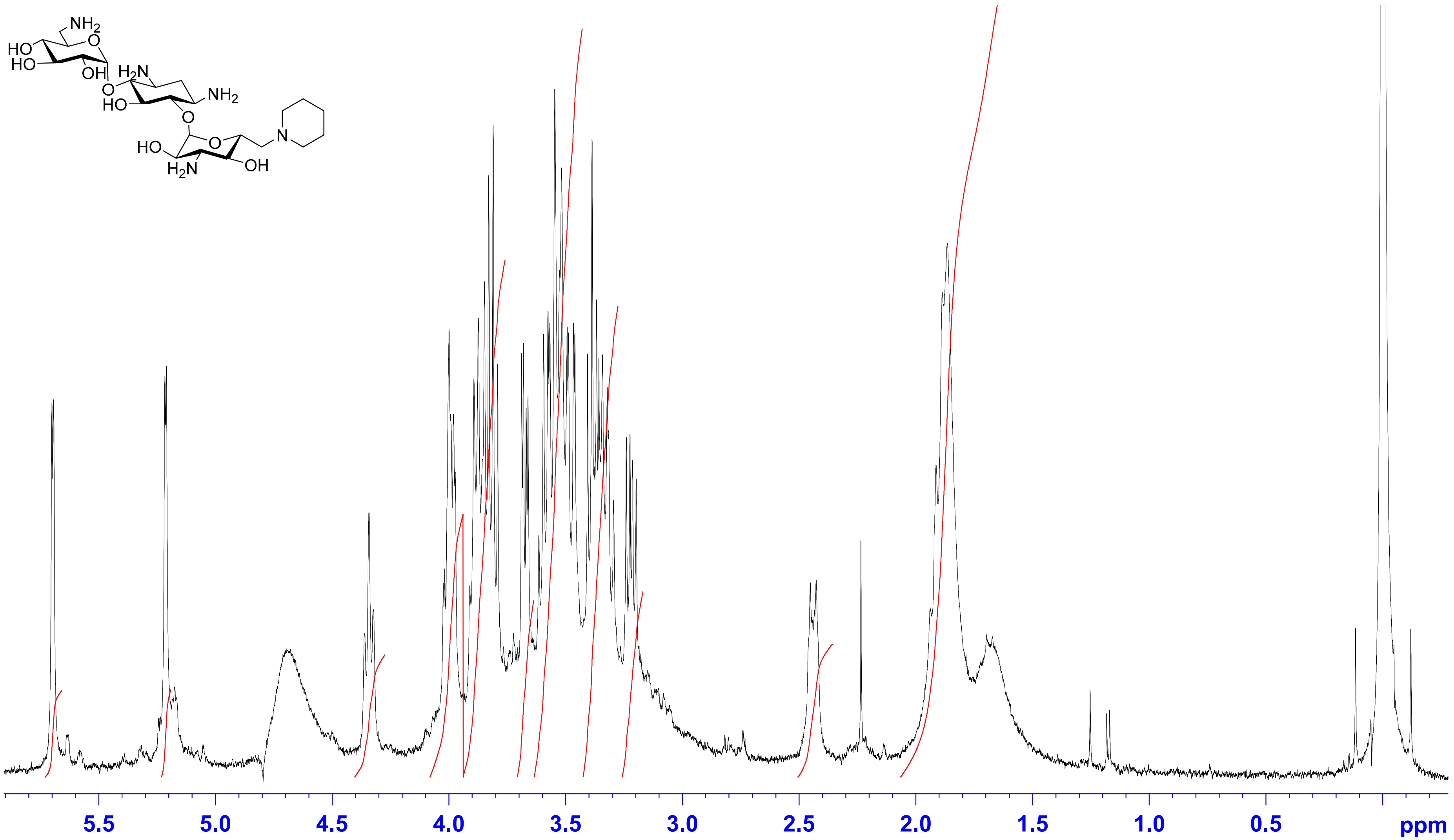

Figure S9. ^{1}H NMR (500.2 MHz, D_2O) spectrum of 6"-(piperidine-1-ium)-6"-deoxykanamycin A (**4**)

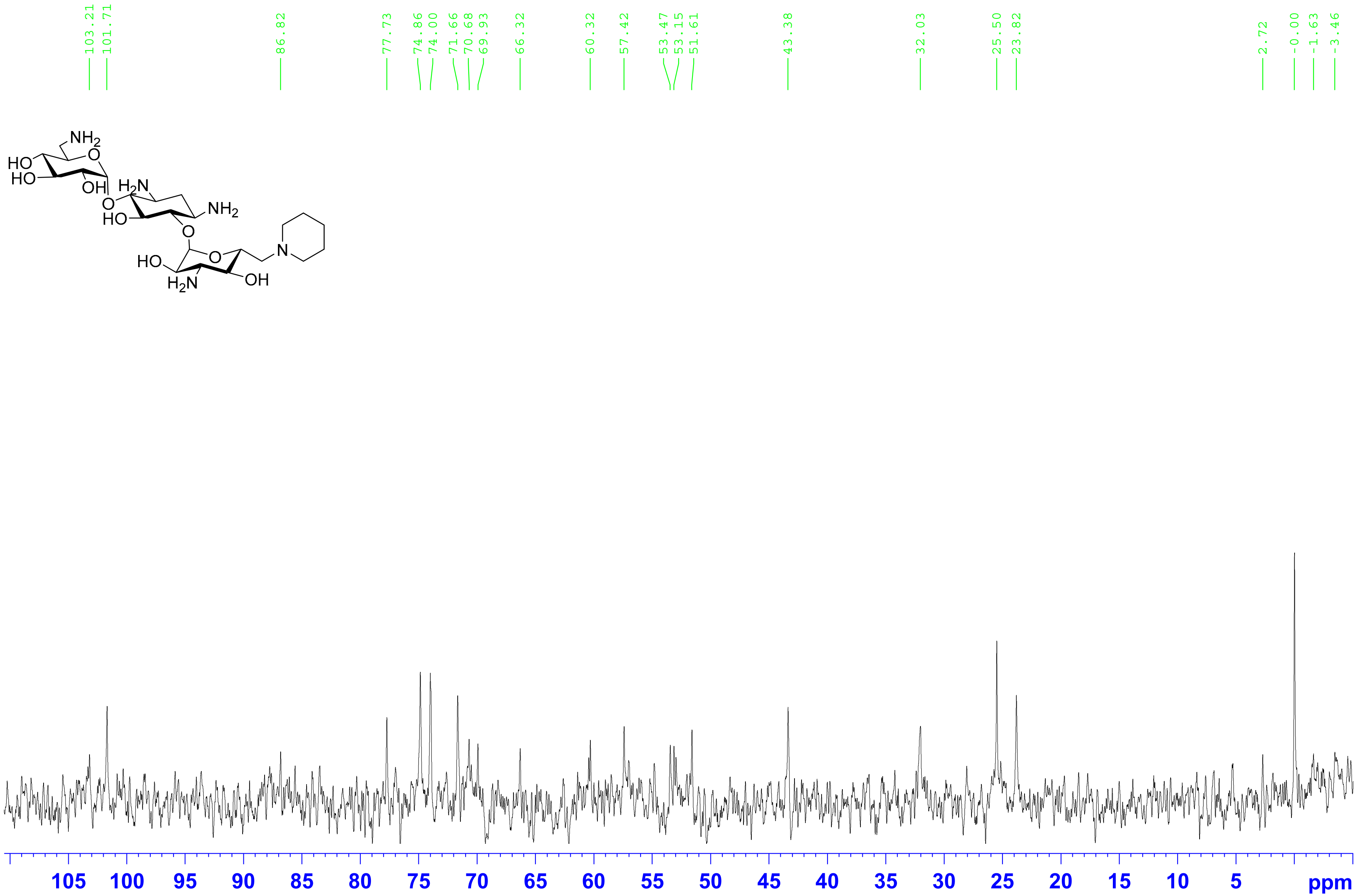

Figure S10. ^{13}C NMR (125.8 MHz, D_2O) spectrum of 6"-(piperidine-1-ium)-6"-deoxykanamycin A (**4**)

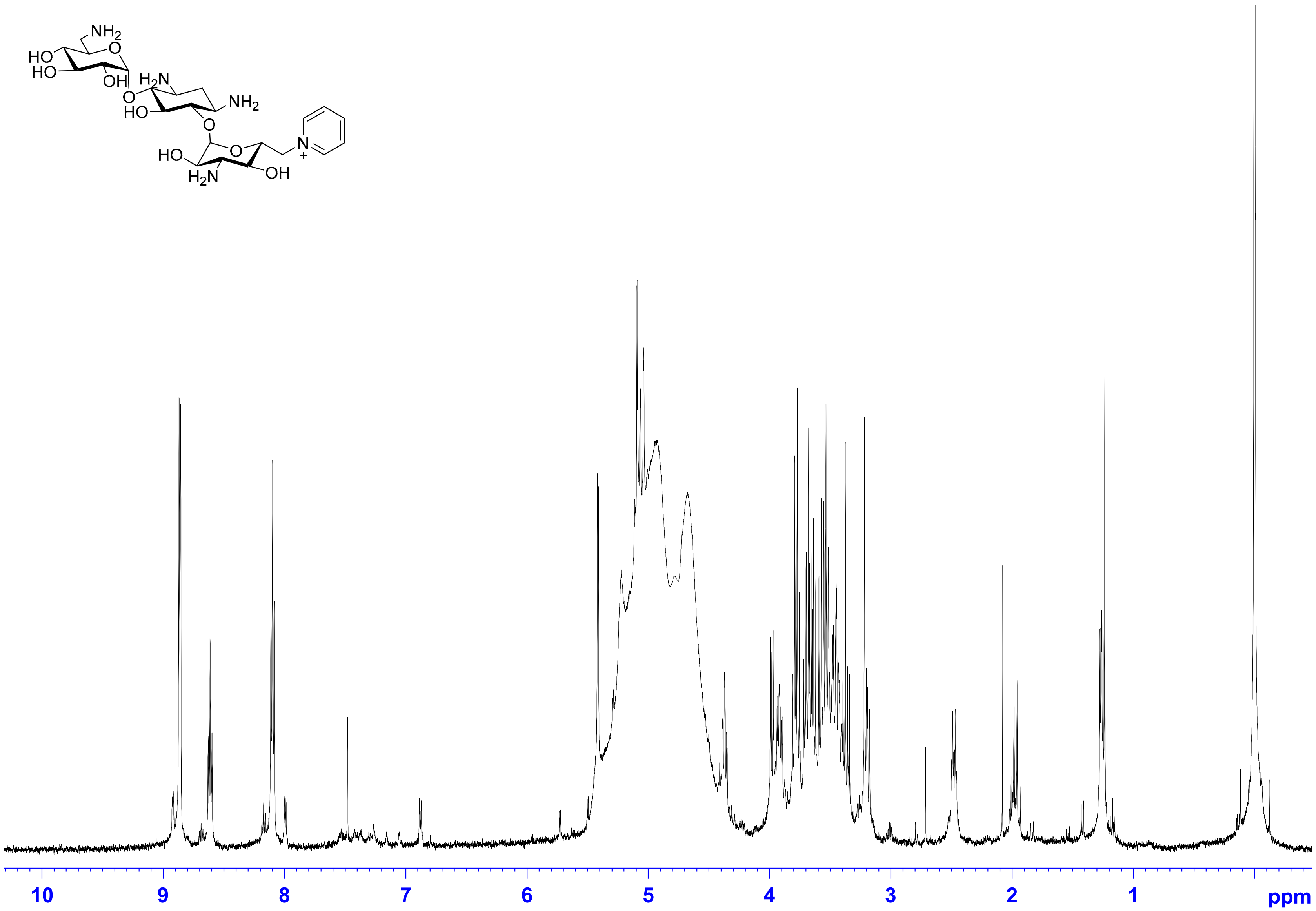

Figure S11. ^{1}H NMR (500.2 MHz, D_2O) spectrum of 6”-(pyridine-1-ium)-6”-deoxykanamycin A (**5**)

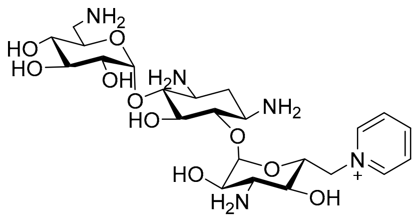

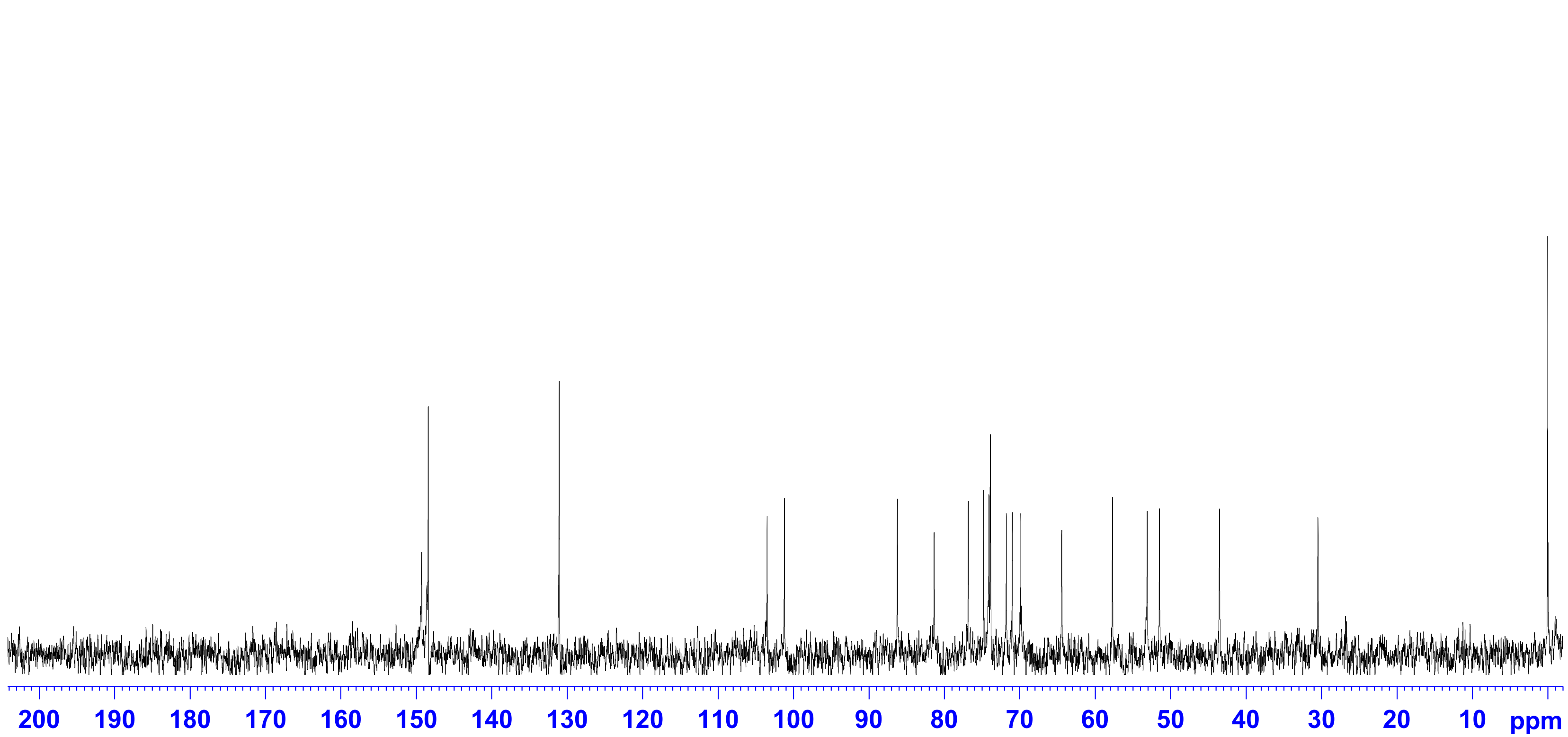

Figure S12. ^{13}C NMR (125.8 MHz, D_2O) spectrum of 6”-(pyridine-1-ium)-6”-deoxykanamycin A (**5**)

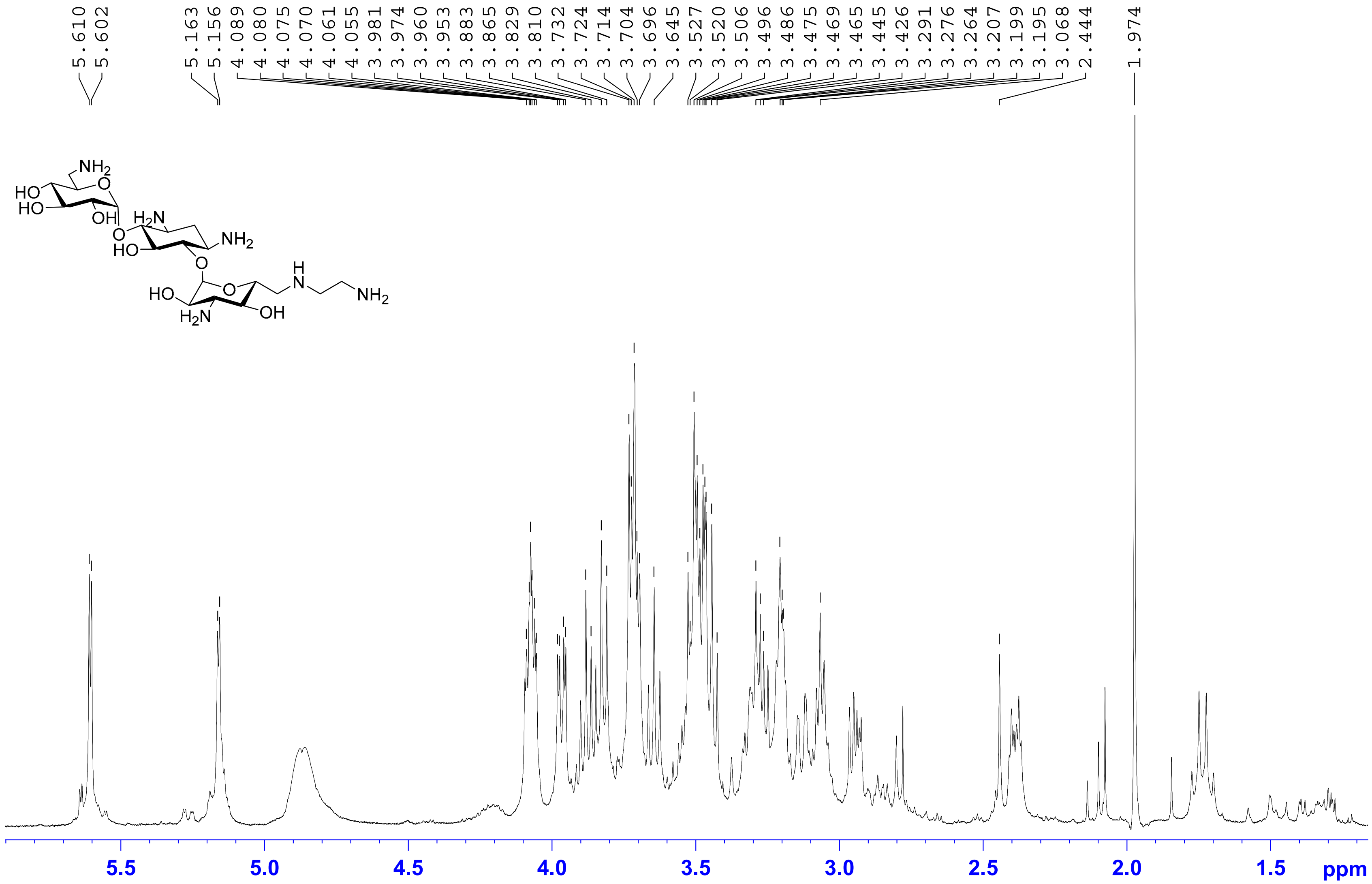

Figure S13. ^{1}H NMR (500.2 MHz, D_2O) spectrum of 6"-(2-aminoethyl-1-amino)-6"-deoxykanamycin A (**6**)

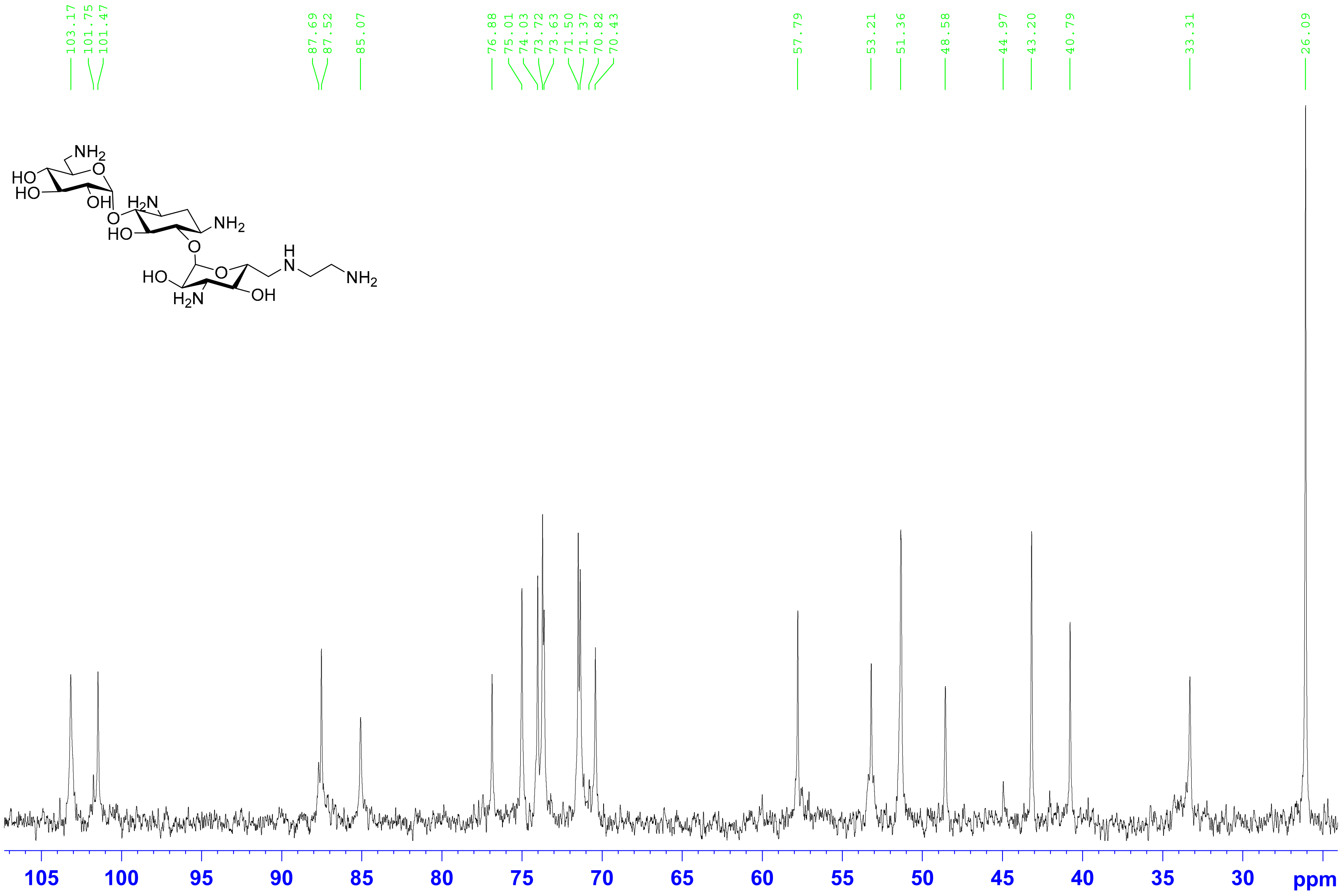

Figure S14. ^{13}C NMR (125.8 MHz, D_2O) spectrum of 6"-(2-aminoethyl-1-amino)-6"-deoxykanamycin A (**6**)

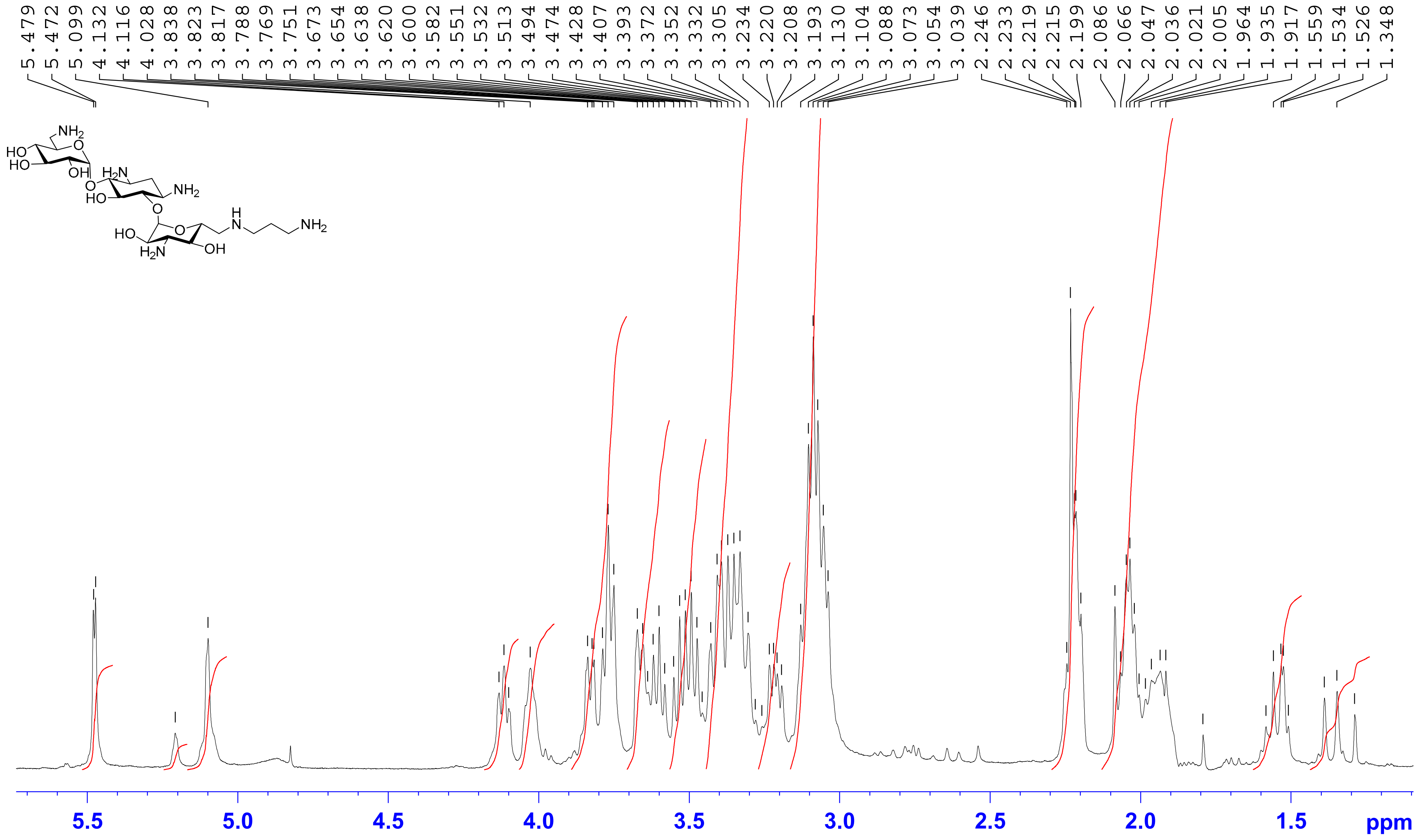

Figure S15. ^{1}H NMR (500.2 MHz, D_2O) spectrum of 6''-(3-aminopropyl-1-amino)-6''-deoxykanamycin A (**7**)

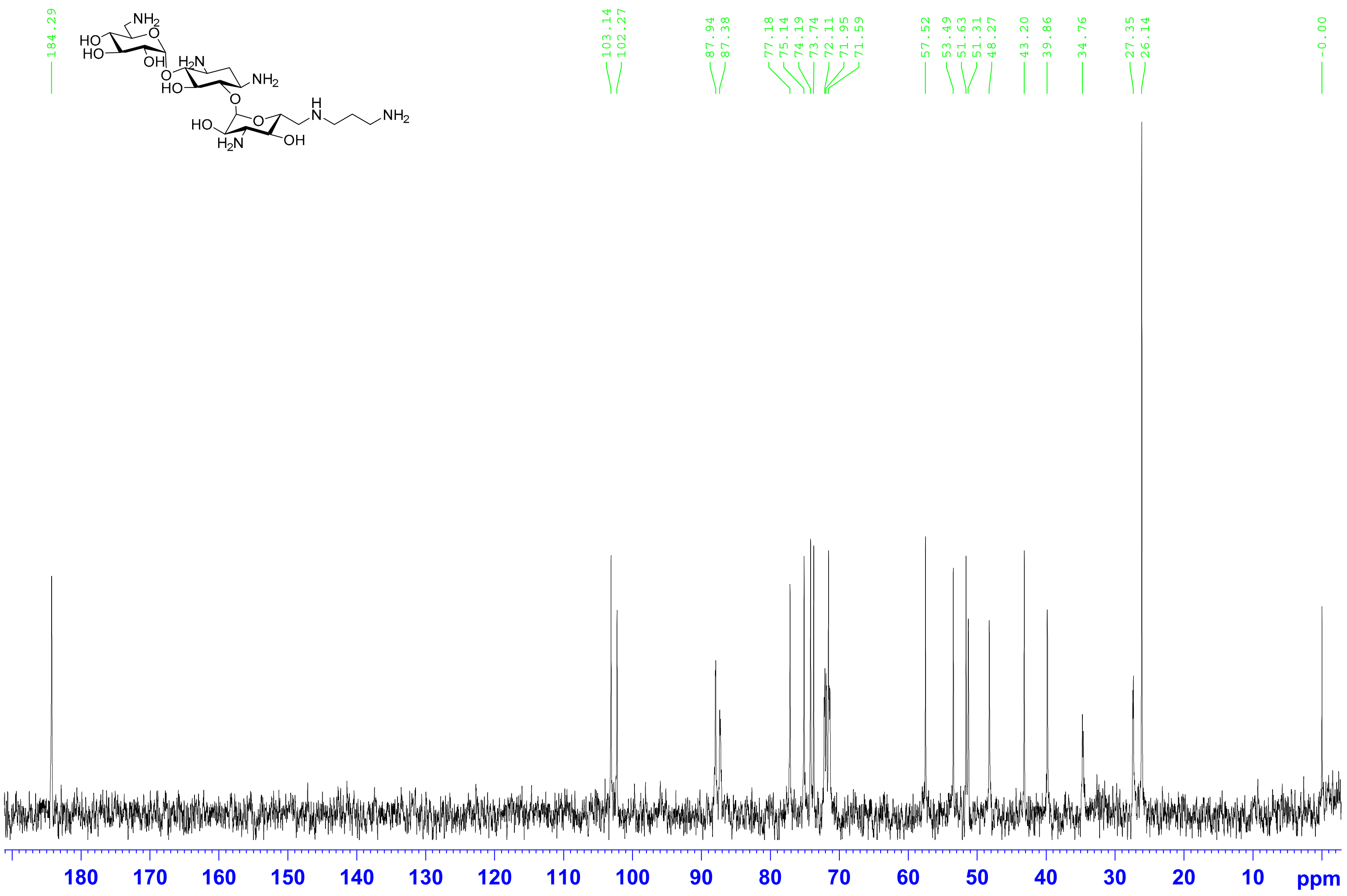

Figure S16. ^{13}C NMR (125.8 MHz, D_2O) spectrum of 6"-(3-aminopropyl-1-amino)-6"-deoxykanamycin A (**7**)

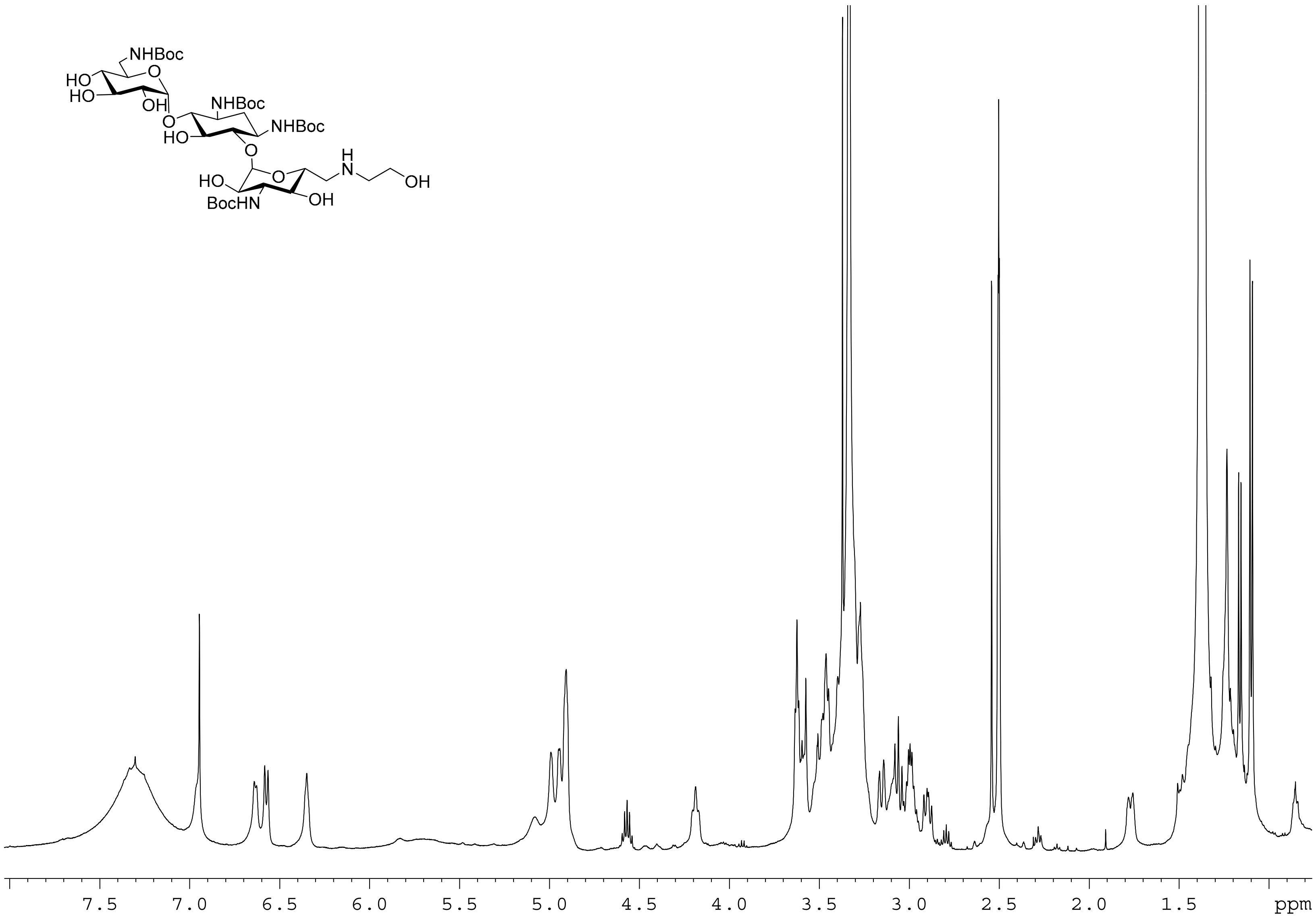

Figure S17. ^{1}H NMR (500.2 MHz, d6-DMSO) spectrum of 6”-(2-hydroxyethyl-1-amino)-1,3,6’,3”-tetra-N-Boc-6”-deoxykanamycin A (**8a**)

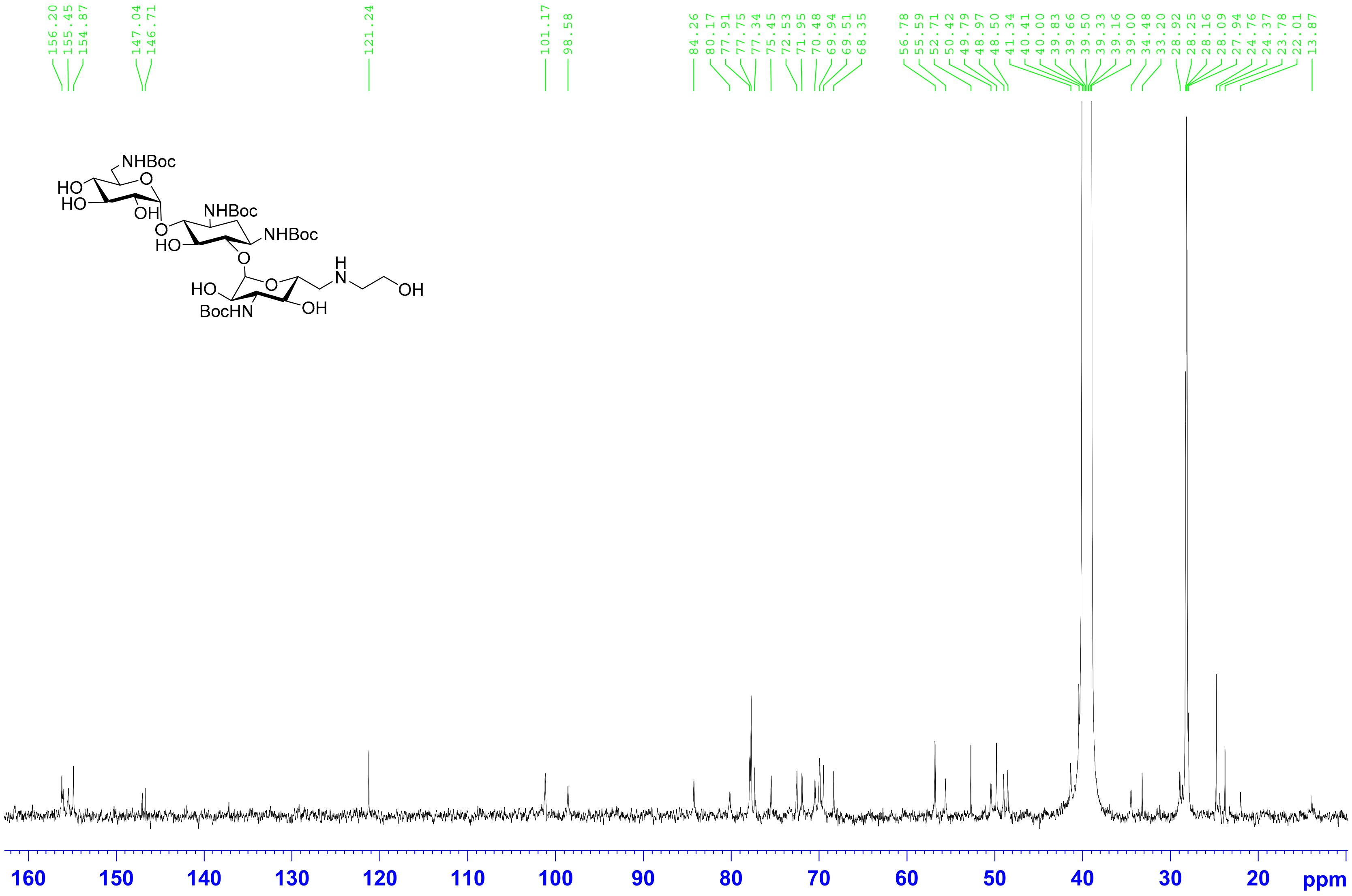

Figure S18. ^{13}C NMR (125.8 MHz, d6-DMSO) spectrum of 6”-(2-hydroxyethyl-1-amino)-1,3,6’,3”-tetra-N-Boc-6”-deoxykanamycin A (**8a**)

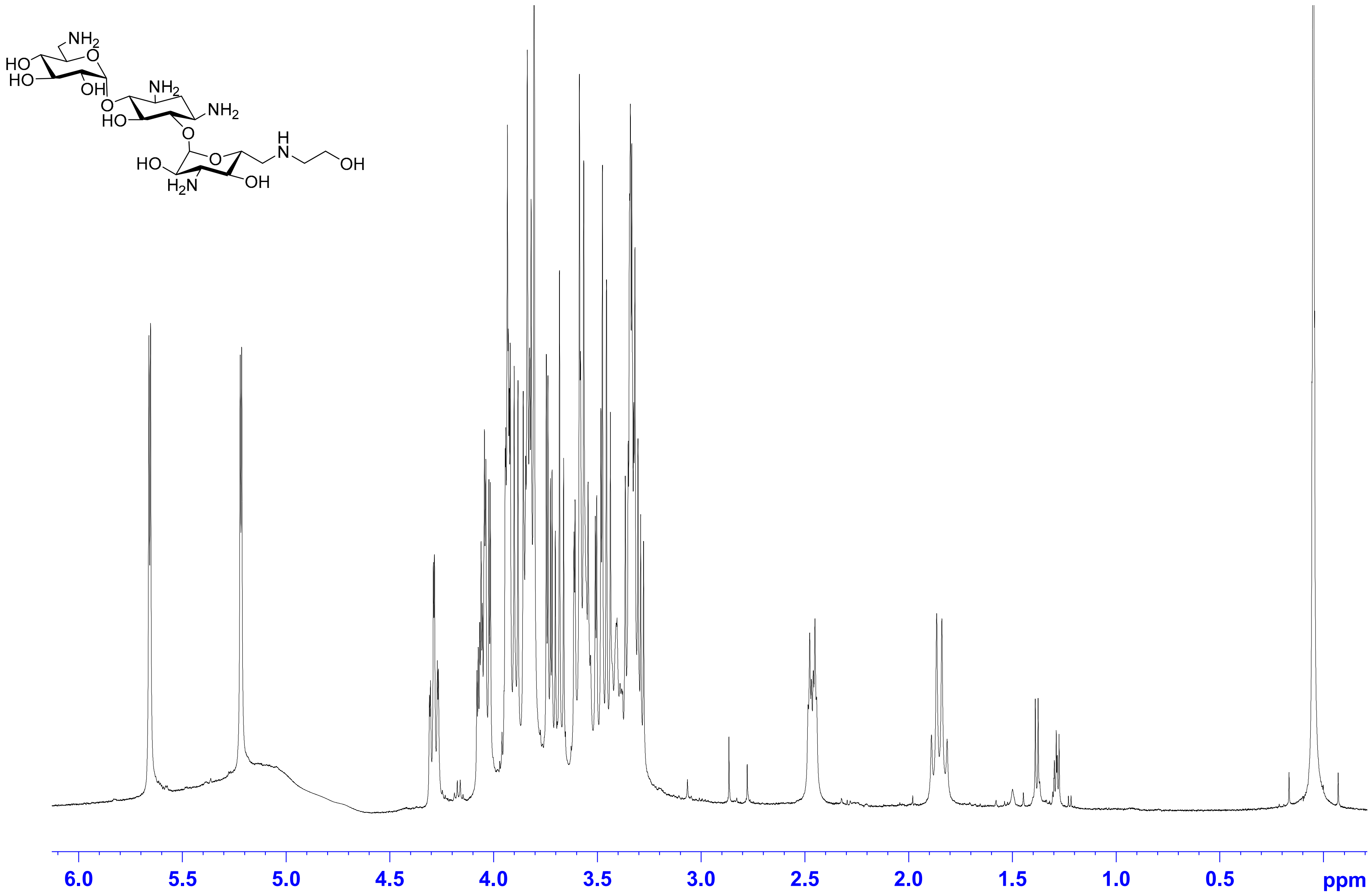

Figure S19. ^{1}H NMR (500.2 MHz, D_2O) spectrum of 6”-(2-hydroxyethyl-1-amino)-6”-deoxykanamycin A (**8**)

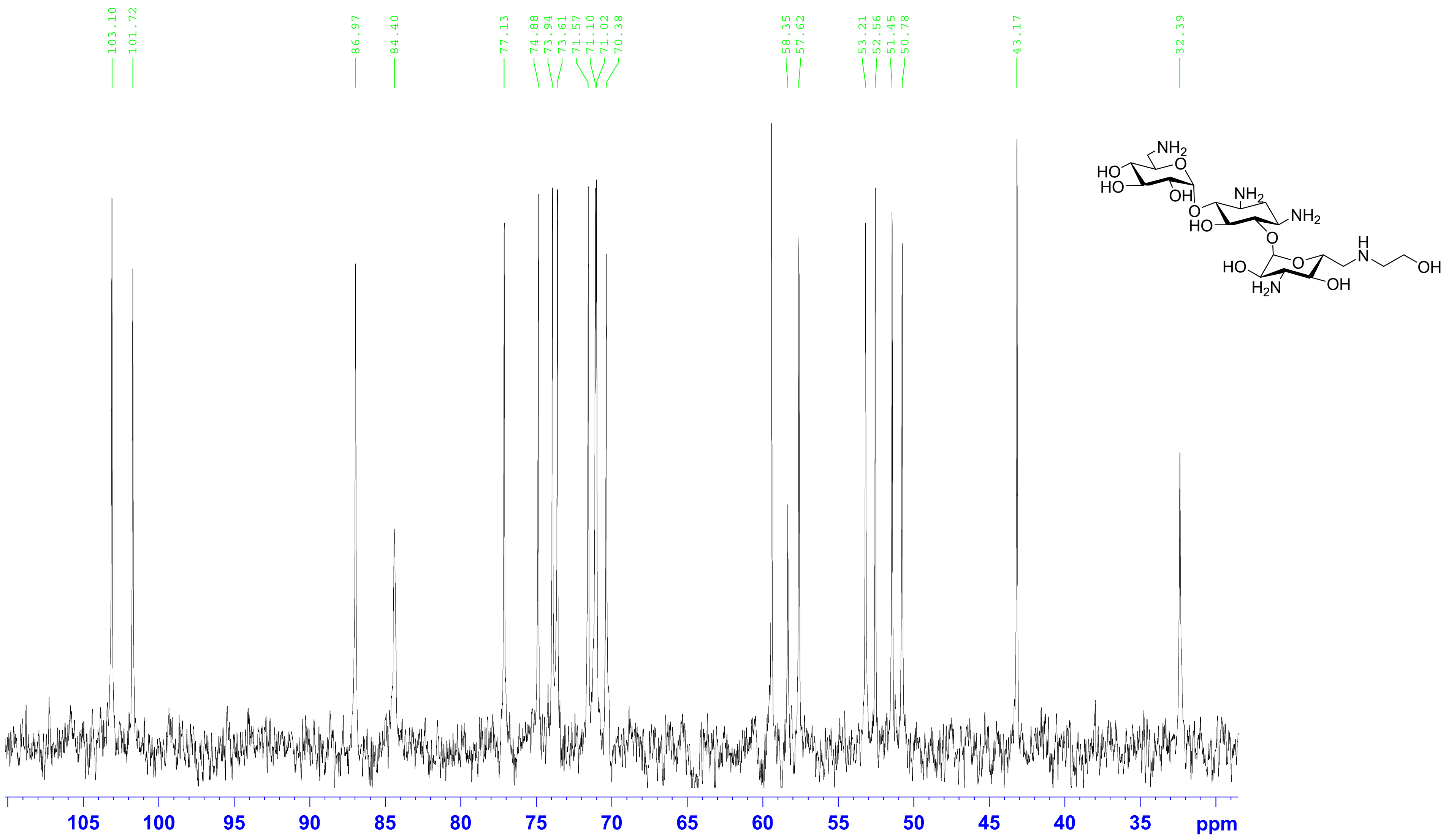

Figure S20. ^{13}C NMR (125.8 MHz, D_2O) spectrum of 6”-(2-hydroxyethyl-1-amino)-6”-deoxykanamycin A (**8**)

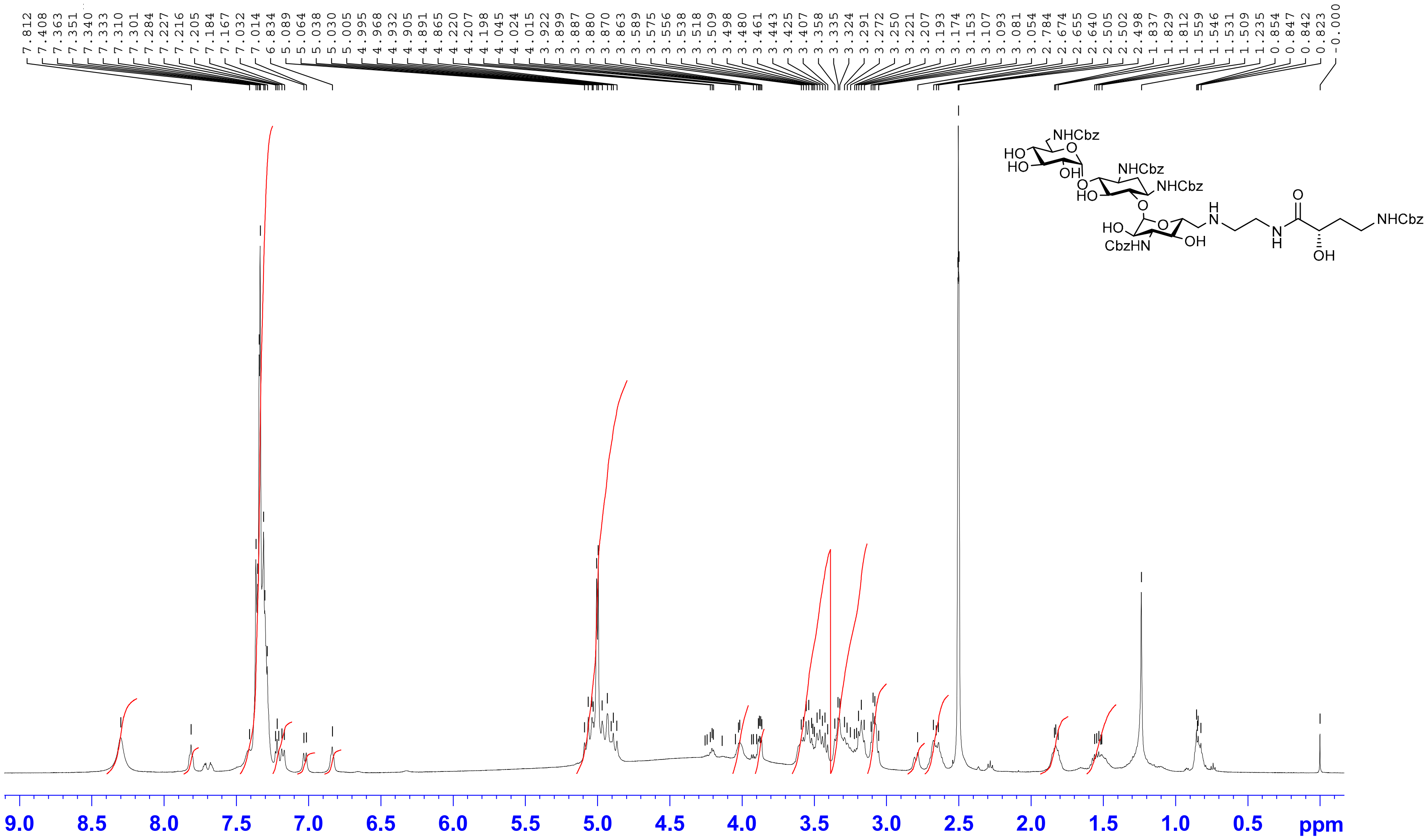

Figure S21. ^{1}H NMR (500.2 MHz, d6-DMSO) spectrum of 6”-(2-((*S*)-4-(((benzyloxy)carbonyl)amino)-2-hydroxy)-N-(ethyl-1-amino)butanamide))-1,3,6',3” -tetra-N-Cbz-6”-deoxykanamycin A (**9a**)

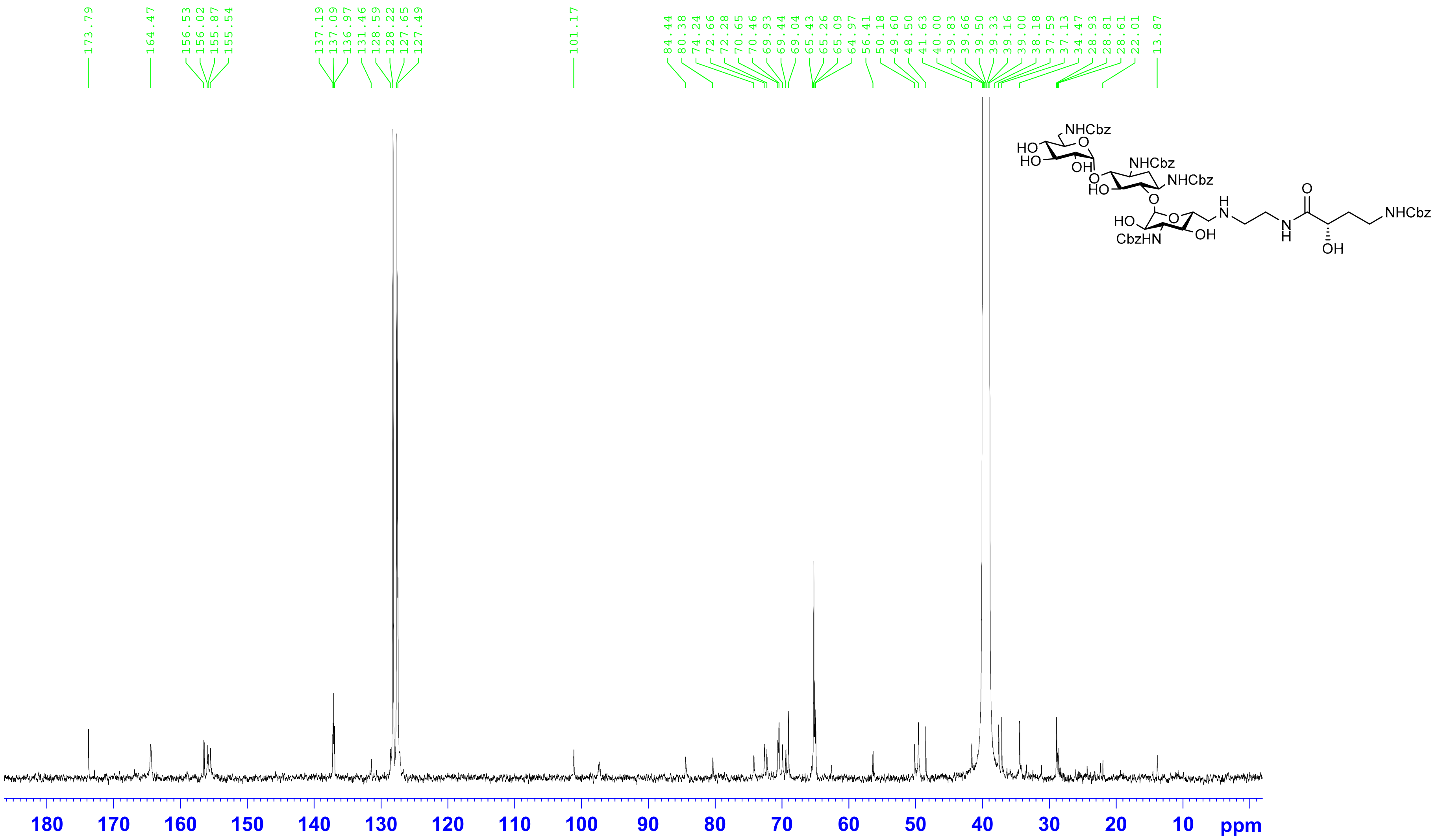

Figure S22. ^{13}C NMR (125.8 MHz, d6-DMSO) spectrum of 6”-(2-((*S*)-4-(((benzyloxy)carbonyl)amino)-2-hydroxy)-N-(ethyl-1-amino)butanamide))-1,3,6',3” -tetra-N-Cbz-6”-deoxykanamycin A (**9a**)

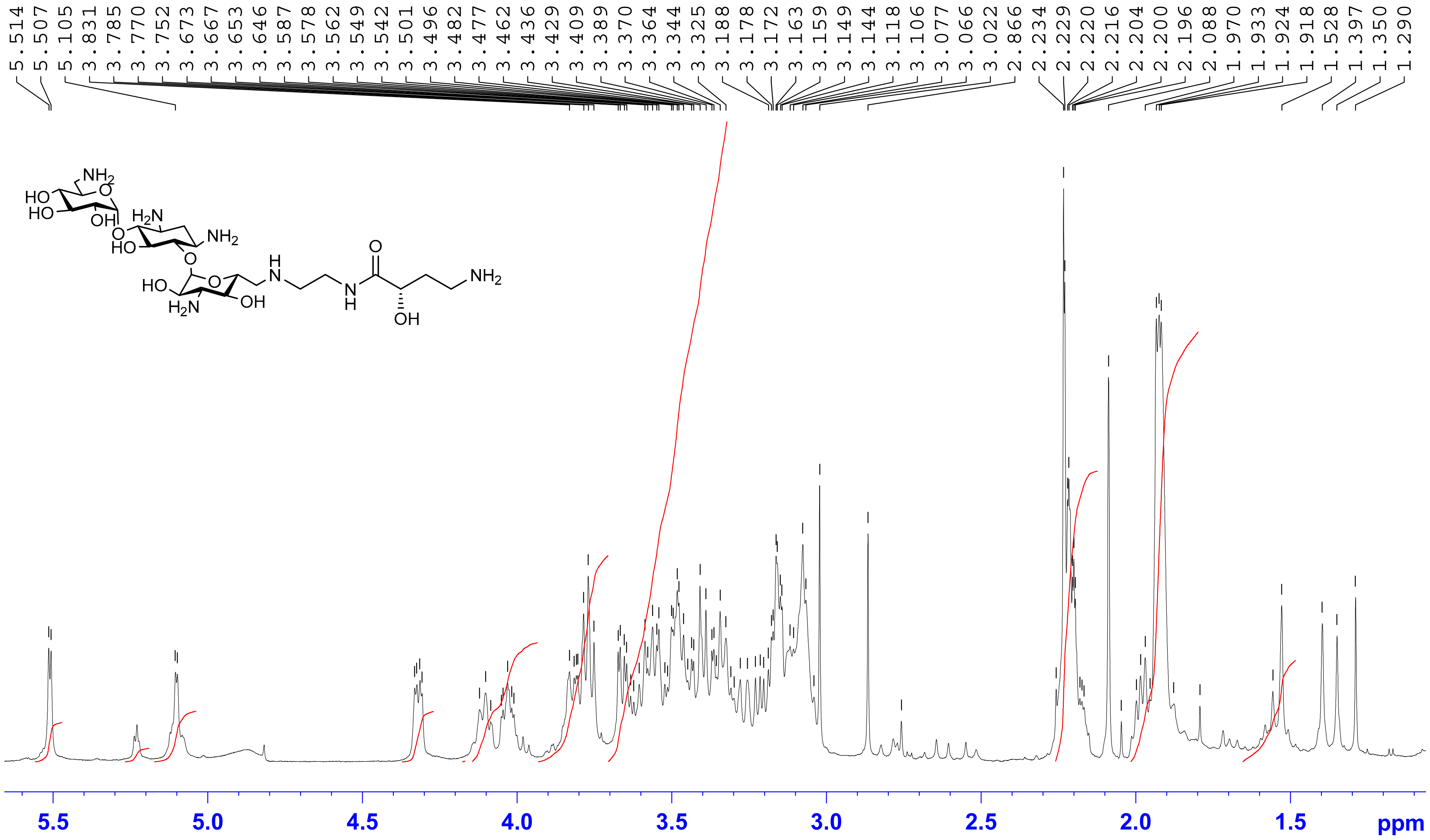

Figure S23. ¹H NMR (500.2 MHz, D_2O) spectrum of 6”-(2-((*S*)-4-amino-2-hydroxy)-N-(ethyl-1-amino)butanamide))-6”-deoxykanamycin A (**9**)

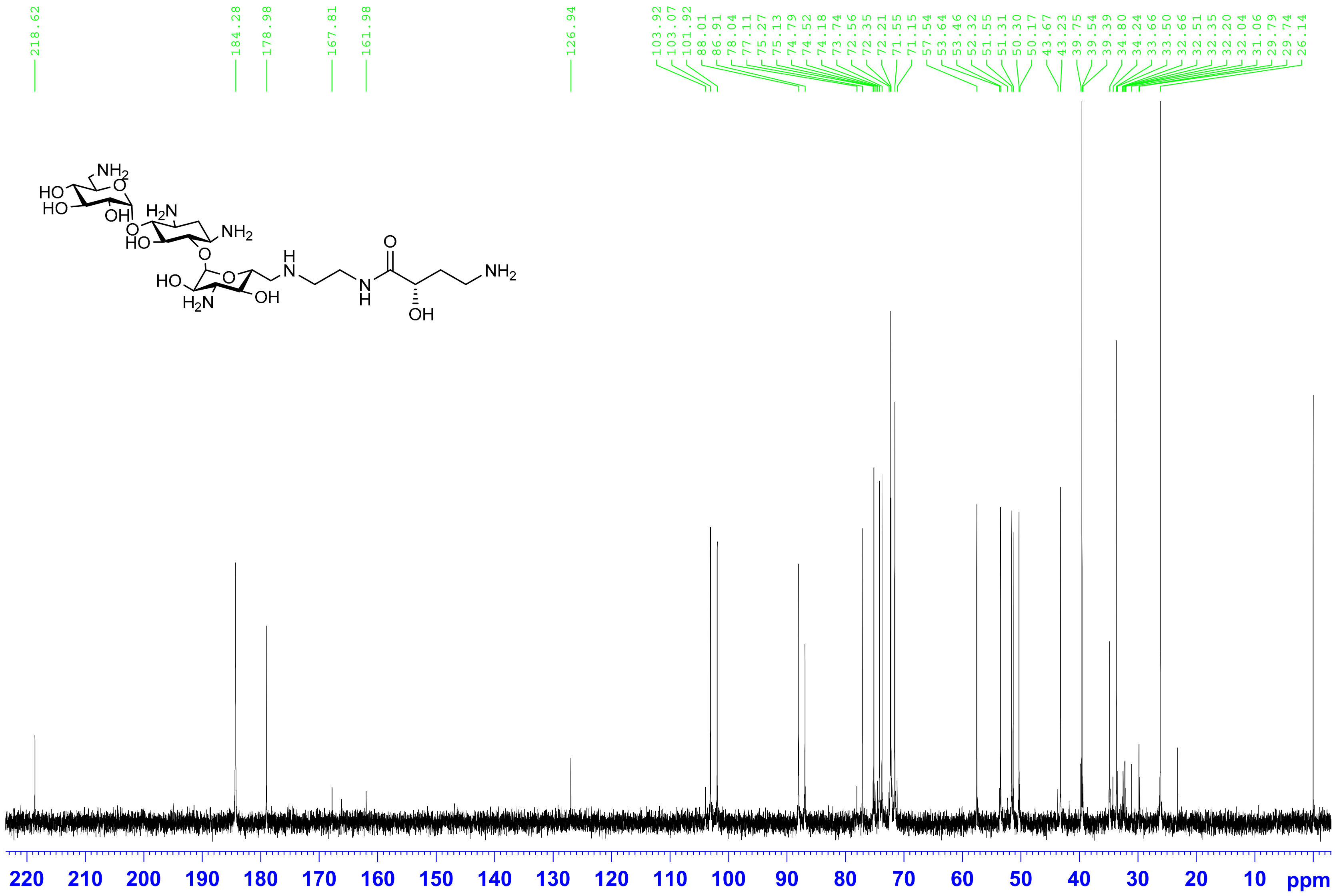

Figure S24. ^{13}C NMR (125.8 MHz, D_2O) spectrum of 6”-(2-((*S*)-4-amino-2-hydroxy)-N-(ethyl-1-amino)butanamide))-6”-deoxykanamycin A (**9**)

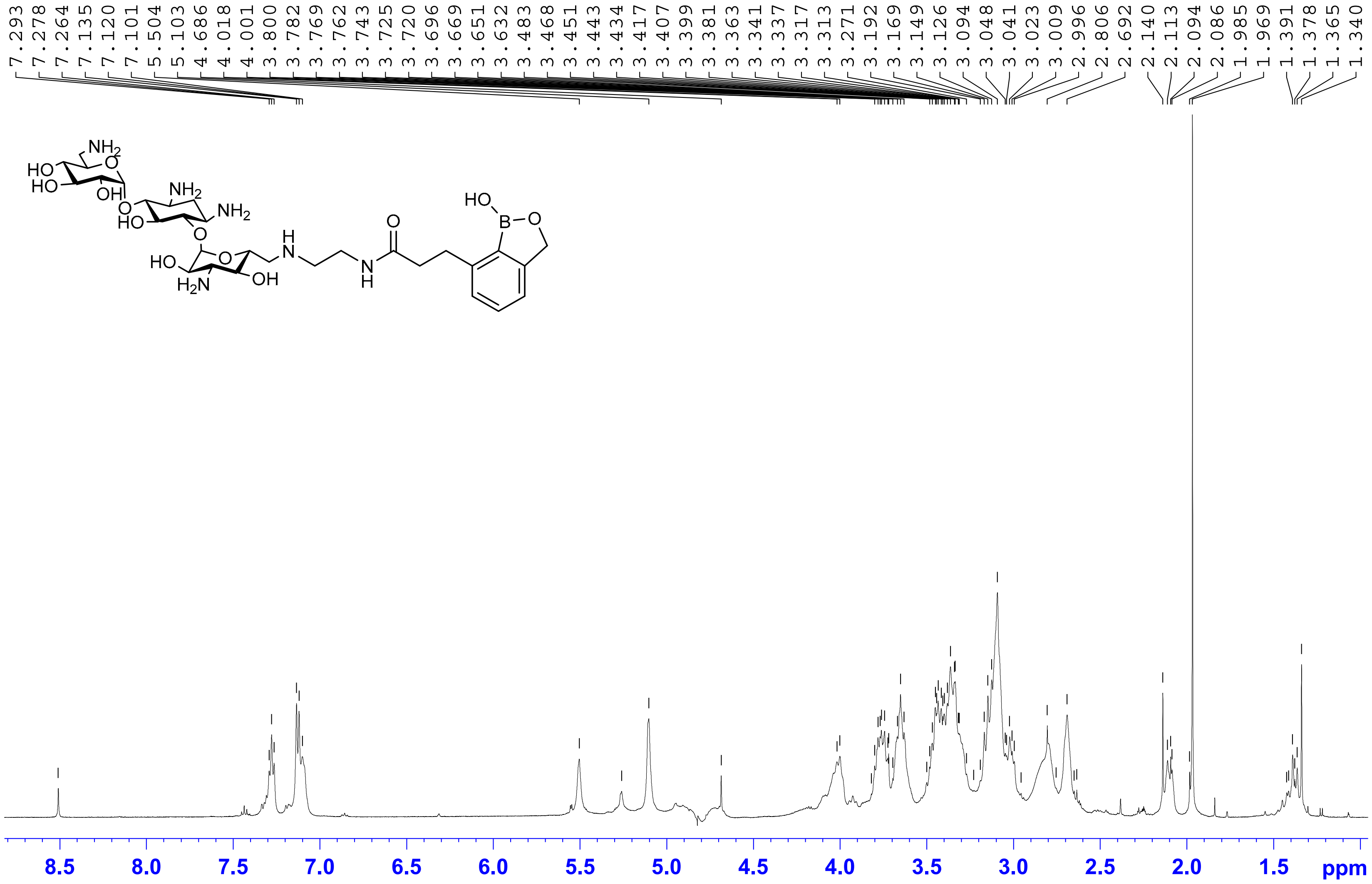

Figure S25. ^{1}H NMR (500.2 MHz, D_2O) spectrum of 6”-(2-(3-(1-hydroxy-1,3-dihydrobenzo[c][1,2]oxaborol-7-yl)-N-(ethyl-1-amino)propanamide)-6”-deoxykanamycin A (**10**)

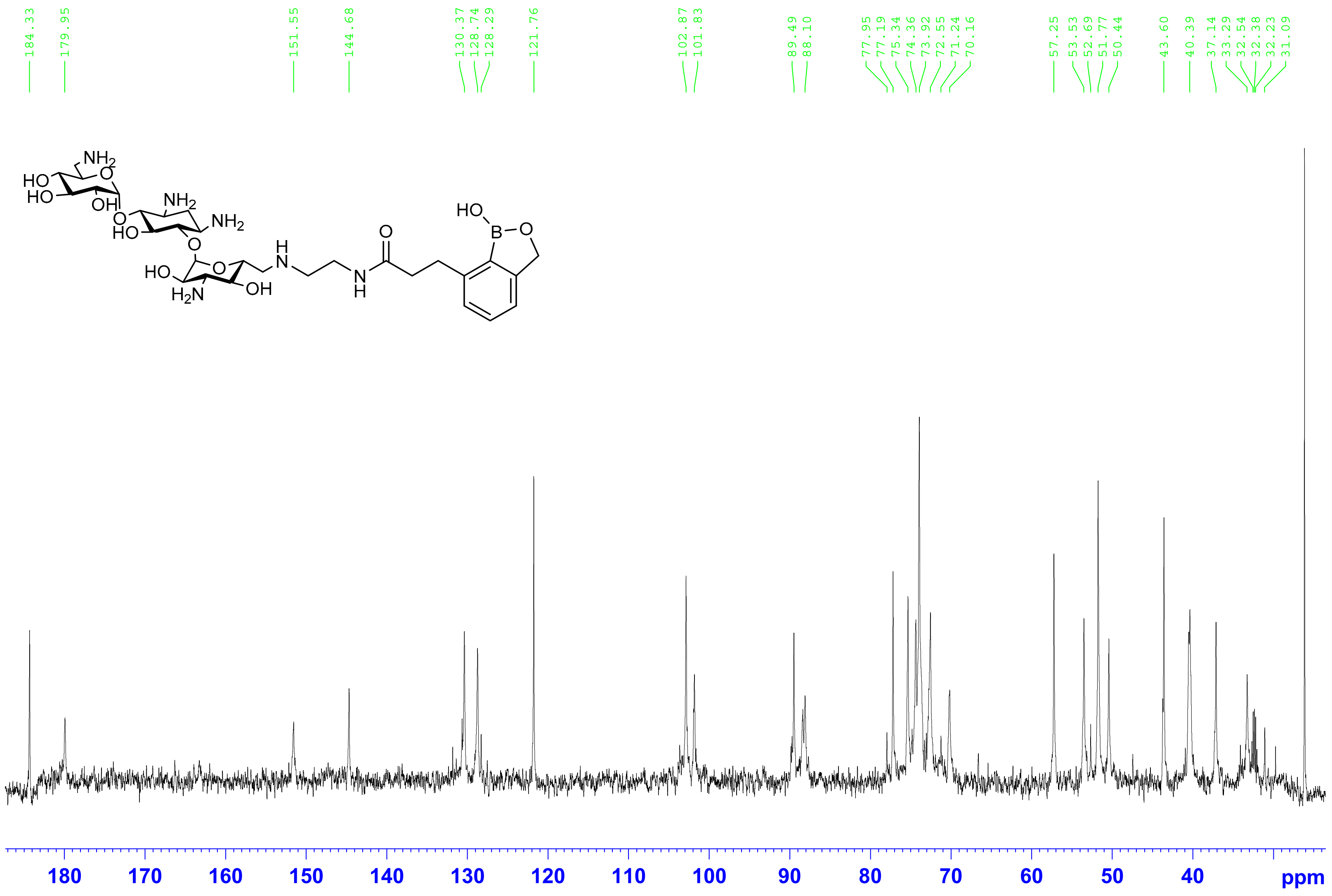

Figure S26. ^{13}C NMR (125.8 MHz, D2O) spectrum of 6"-(2-(3-(1-hydroxy-1,3-dihydrobenzo[c][1,2]oxaborol-7-yl)-N-(ethyl-1-amino)propanamide)-6"-deoxykanamycin A (**10**)

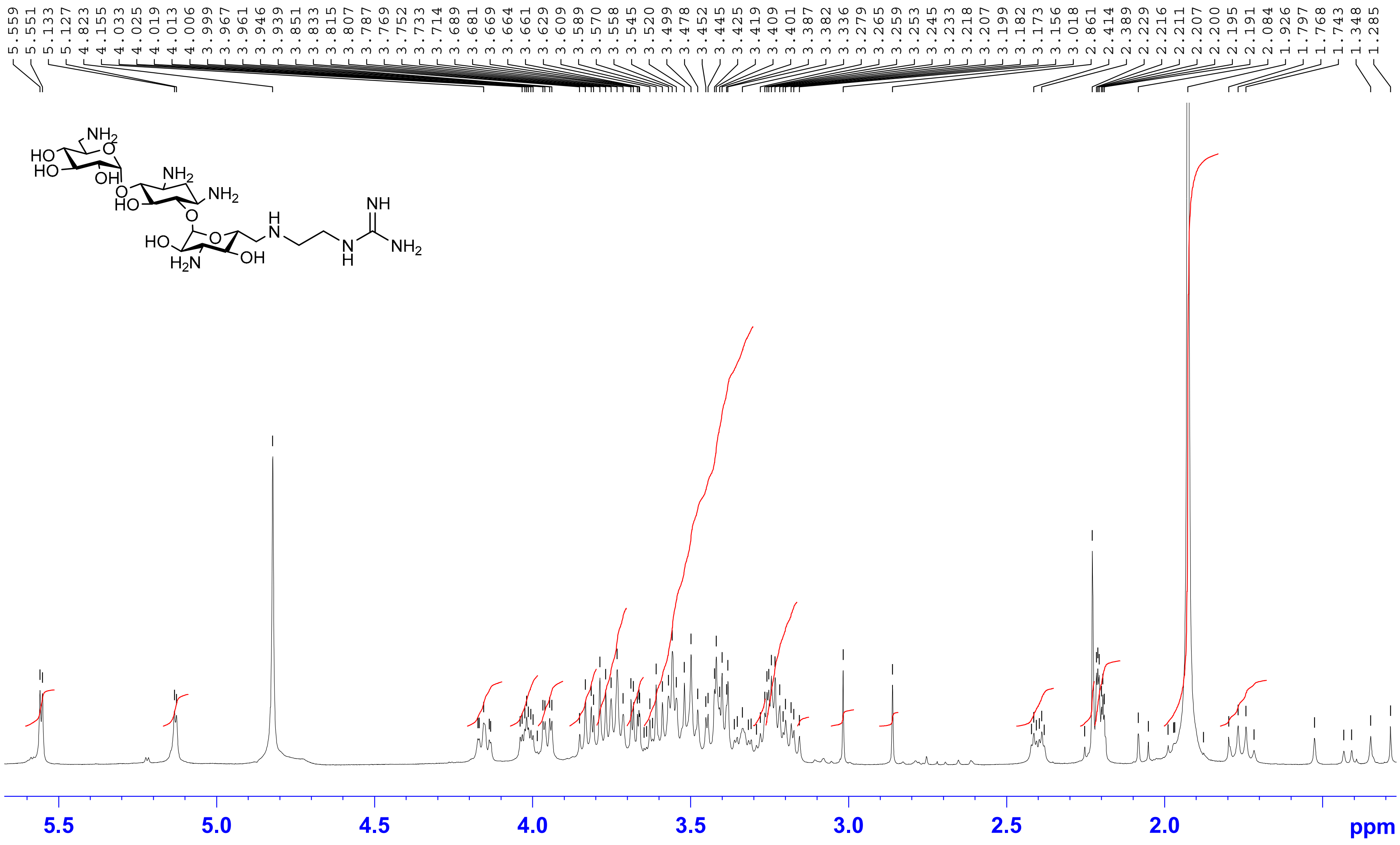

Figure S27. ^{1}H NMR (500.2 MHz, D_2O) spectrum of 6”-(2-aminoguanidinethyl-1-amino)-6”-deoxykanamycin A (**11**)

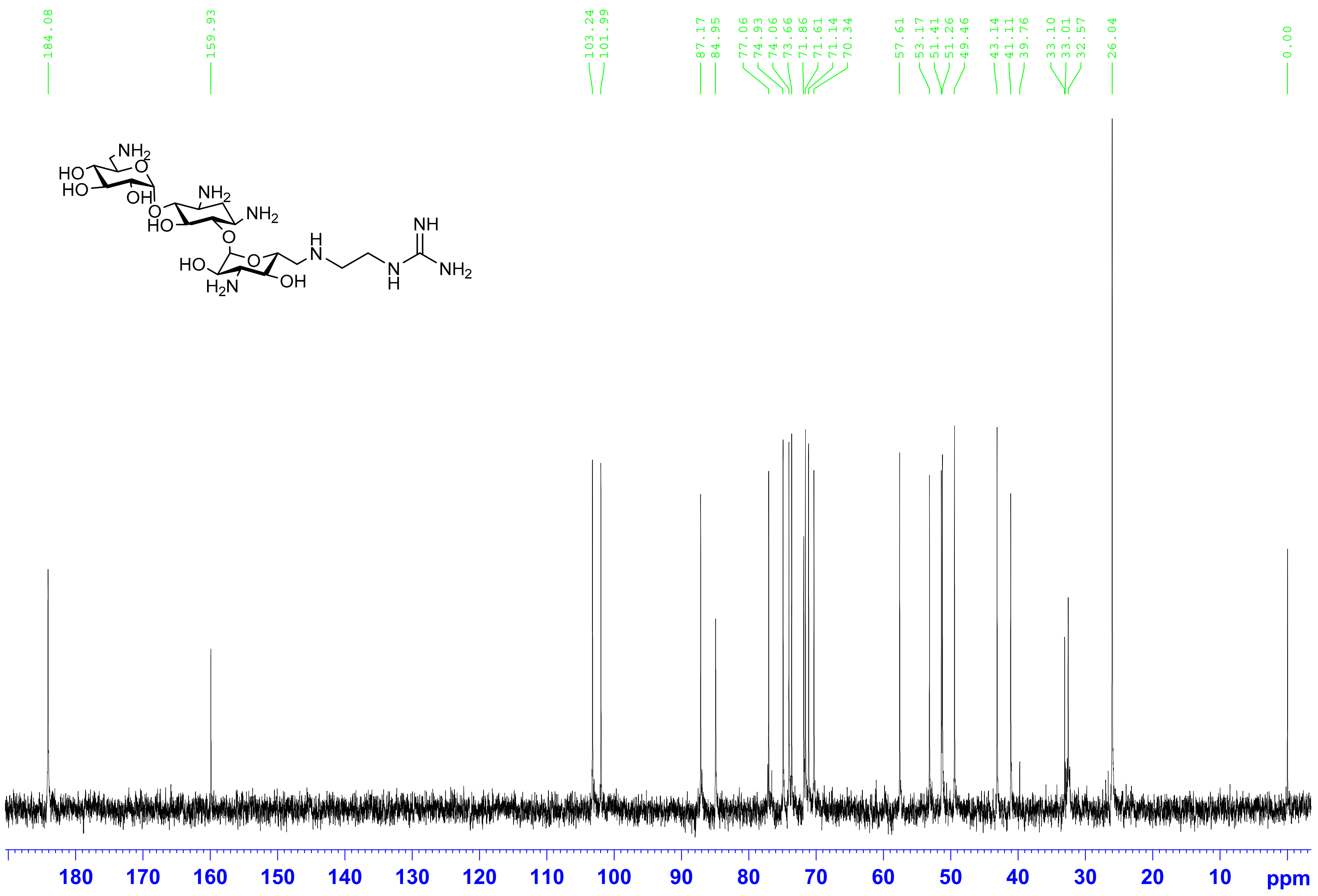

Figure S28. ^{13}C NMR (125.8 MHz, D_2O) spectrum of 6”-(2-aminoguanidinethyl-1-amino)-6”-deoxykanamycin A (**11**)

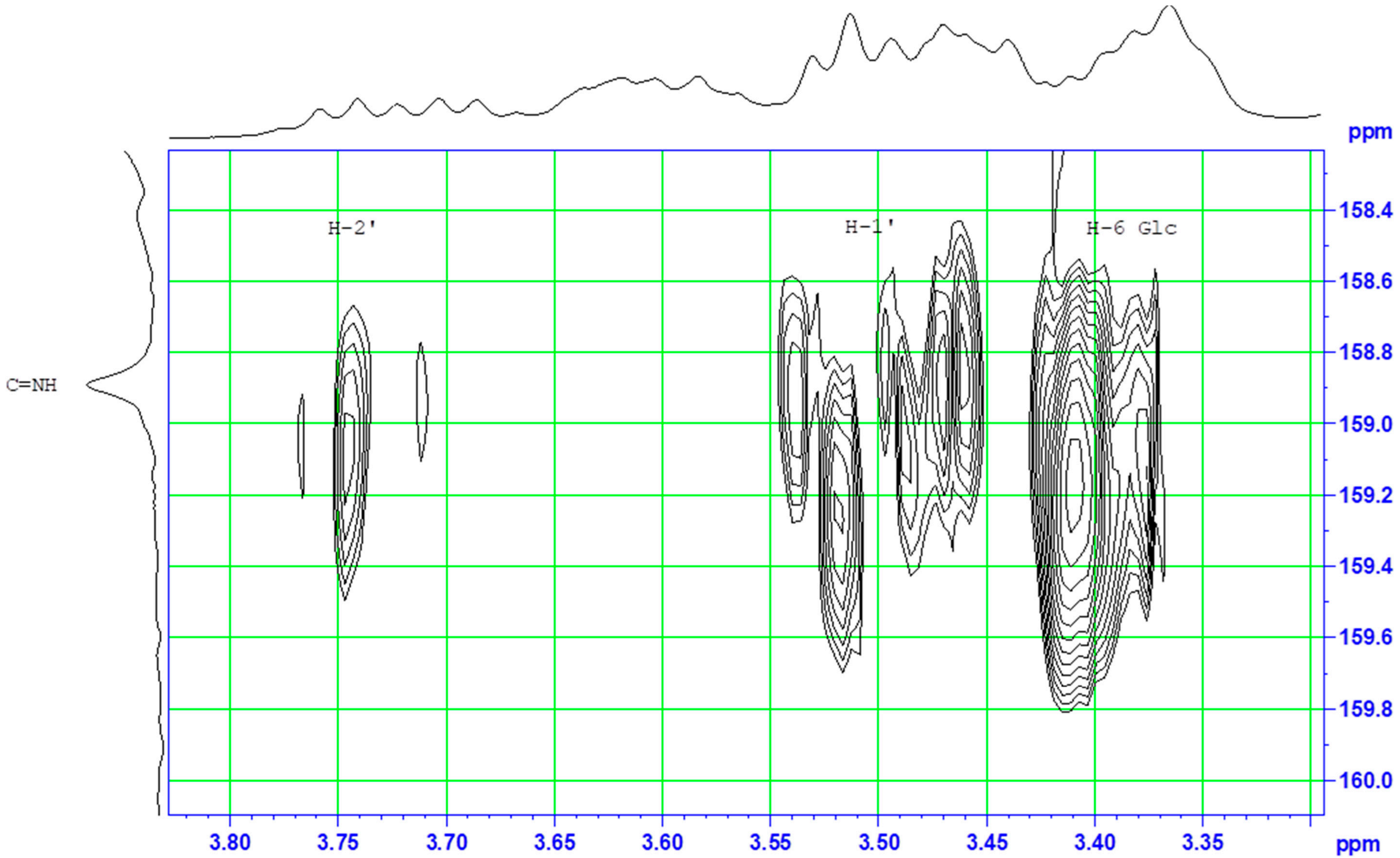

Figure S29. Fragment of the HMBC ^{1}H-^{13}C spectrum of compound **12**.

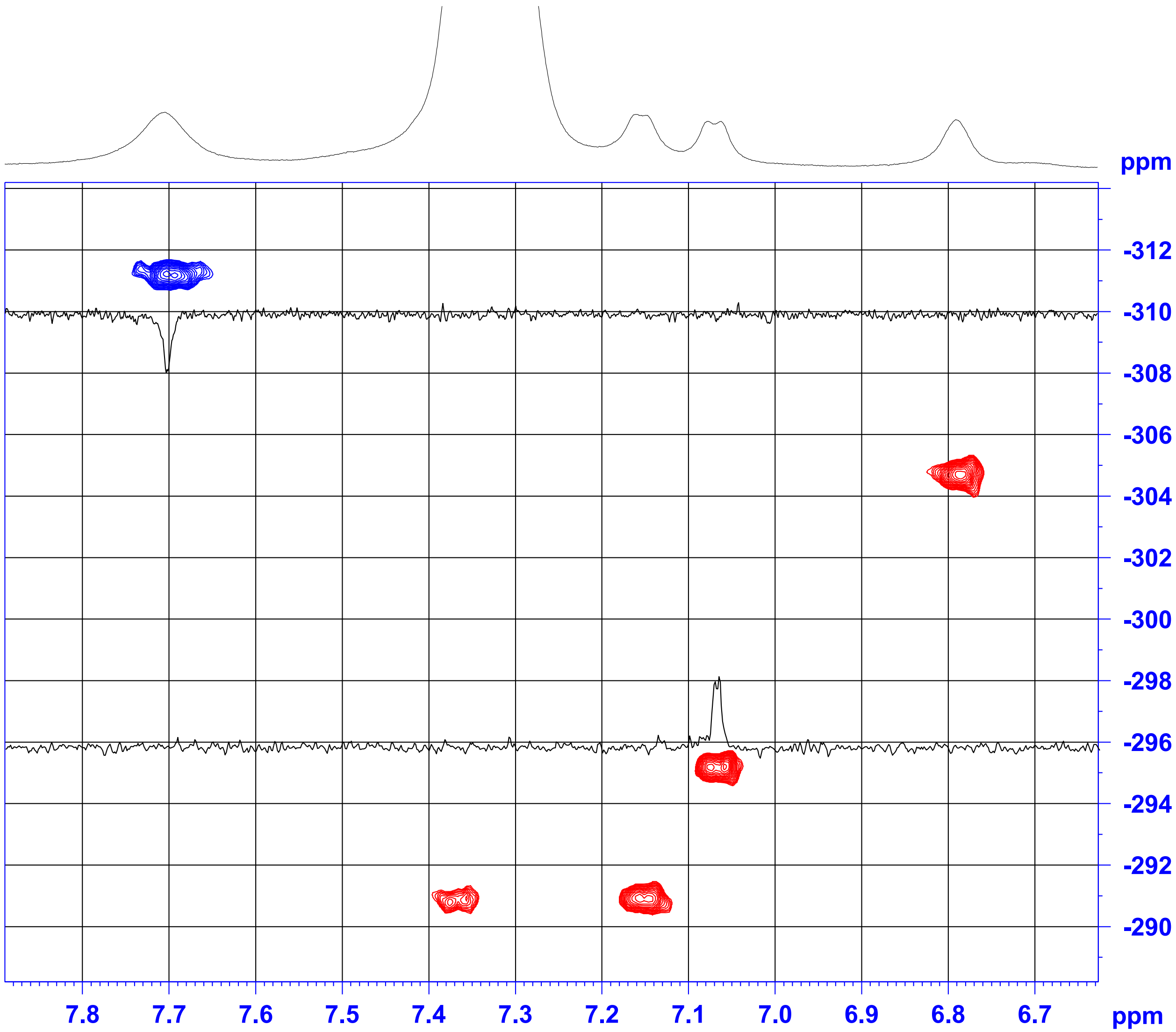

Figure S30. ^{1}H-^{15}N edHSQC spectrum of compound **12**. Insets show cross sections of the two-dimensional spectrum through the corresponding signals. The signal of the NH_2 group at -311.2 ppm has negative polarity.

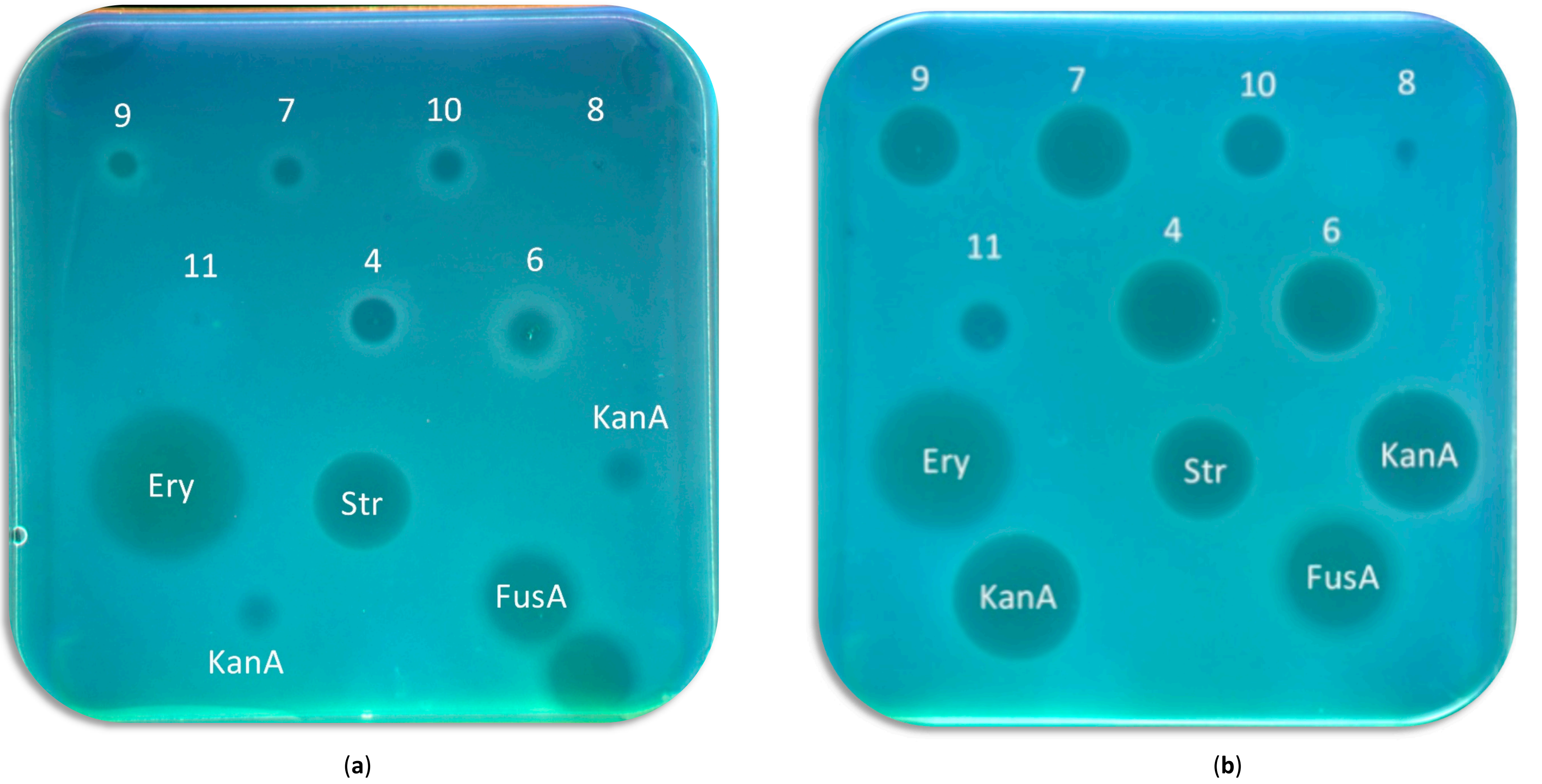

Figure S31. The antibacterial activity of the kanamycin A derivatives **4**, **6–11** and reference antibiotics (KanA – Kanamycin A; Ery – Erythromycin; Str – Streptomycin; FusA – Fusidic acid) against *E. coli* BW25113 (the diffusion in agar method): **a**) resistant strain bearing aminoglycoside-3'-phosphotransferase gene; **b**) wild type strain *E. coli*. Concentration of each antibiotic 50 mg/ml, volume of droplet – 1 µl.
